# Cyclic peptides can engage a single binding pocket through highly divergent modes

**DOI:** 10.1073/pnas.2003086117

**Published:** 2020-10-12

**Authors:** Karishma Patel, Louise J. Walport, James L. Walshe, Paul D. Solomon, Jason K. K. Low, Daniel H. Tran, Kevork S. Mouradian, Ana P. G. Silva, Lorna Wilkinson-White, Alexander Norman, Charlotte Franck, Jacqueline M. Matthews, J. Mitchell Guss, Richard J. Payne, Toby Passioura, Hiroaki Suga, Joel P. Mackay

**Affiliations:** ^a^School of Life and Environmental Sciences, The University of Sydney, Sydney, NSW 2006, Australia;; ^b^Protein–Protein Interaction Laboratory, The Francis Crick Institute, London NW1 1AT, United Kingdom;; ^c^Department of Chemistry, Molecular Sciences Research Hub, Imperial College London, London W12 0BZ, United Kingdom;; ^d^Department of Chemistry, Graduate School of Science, The University of Tokyo, Tokyo 113-0033, Japan;; ^e^School of Chemistry, The University of Sydney, Sydney, NSW 2006, Australia;; ^f^Sydney Analytical Core Research Facility, The University of Sydney, NSW 2006, Australia

**Keywords:** de novo cyclic peptides, BET bromodomain inhibition, structural biology, BRD3, BRD4

## Abstract

Cyclic peptide library screening technologies show immense promise for identifying drug leads and chemical probes for challenging targets. However, the structural and functional diversity encoded within such libraries is largely undefined. We have systematically profiled the affinity, selectivity, and structural features of library-derived cyclic peptides selected to recognize three closely related targets: the acetyllysine-binding bromodomain proteins BRD2, -3, and -4. We report affinities as low as 100 pM and specificities of up to 10^6^-fold. Crystal structures of 13 peptide–bromodomain complexes reveal remarkable diversity in both structure and binding mode, including both α-helical and β-sheet structures as well as bivalent binding modes. The peptides can also exhibit a high degree of structural preorganization. Our data demonstrate the enormous potential within these libraries to provide diverse binding modes against a single target, which underpins their capacity to yield highly potent and selective ligands.

## Significance

Large DNA-encoded libraries of cyclic peptides are emerging as powerful sources of molecules to tackle challenging drug targets. The structural and functional diversity contained within these libraries is, however, little explored. Here we demonstrate that one such library contains members that use unexpectedly diverse mechanisms to recognize the same surface on the same target proteins with high affinity and specificity. This range of binding modes is much larger than observed in natural ligands of the same proteins, demonstrating the power and versatility of the technology. Our data also reveal opportunities for the development of more sophisticated approaches to achieving specificity when trying to selectively target one member of a family of closely related proteins.

Cyclic peptides are emerging as a promising class of ligands for challenging biological targets, including those involving both protein–protein and protein–nucleic acid interactions (1). This promise stems, in major part, from their size, chirality, and dense functionality, which allow development of molecules with exquisite potency and selectivity. A further benefit of cyclic peptides as ligands for protein targets is that these can be rapidly discovered through library technologies (e.g., phage display and messenger RNA [mRNA] display) (2). Indeed, such display platforms have been used to isolate macrocyclic peptide ligands against diverse protein targets, including enzymes, cellular receptors, and growth factors (3–8). Almost universally, these de novo peptides (i.e., not derived from known natural peptides) exhibit significantly greater potency and selectivity for their target than other known ligands. However, while the number of examples of successful de novo cyclic peptide screens is growing, our understanding of the molecular basis for these interactions—and therefore our appreciation of the “binding space” that such peptides operate in—is relatively poor (9). Although several cocrystal structures have been reported, there has been no systematic analysis of the structural diversity available in typical peptide libraries that would permit discrimination between closely related paralogous protein targets.

The human proteome contains numerous highly conserved protein paralogs. Despite significant structural similarity, each may perform distinct functions within a cell. Achieving selectivity between such proteins is therefore an important and challenging goal when developing new chemical tools and therapeutics. A well-studied example of this challenge is the bromodomain family, which consists in humans of 40 different proteins, each containing a highly conserved bromodomain that binds acetylated lysine residues. Within this class of proteins, the bromodomain and extraterminal domain (BET) family contains four members that are transcriptional coregulators and that recognize acetyllysines (AcKs) using a tandem pair of highly homologous bromodomains (BDs) (*SI Appendix*, Fig. S1) (10, 11). Inhibition of the BET family of BDs has shown considerable promise for a range of different pathologies (12–16), including acute myeloid leukemia, lymphoma, and estrogen receptor-positive breast cancer (17–19). Despite heavy academic and industry investment in this area, the generation of paralog-selective inhibitors has proved elusive because of the extremely high sequence similarity between the bromodomains of family members (90 to 95% within the acetyllysine-binding pocket residues).

Here we set out to explore the structural diversity encoded in de novo peptide libraries, with the idea that this diversity might encode members that can distinguish proteins with highly related sequences. Using the highly conserved BET BDs as a model system, we conducted three mRNA display-based RaPID (random nonstandard peptides integrated discovery) screens using a single library (which contains >10^12^ unique members; *SI Appendix*, Fig. S2*A*). We identified numerous very high affinity ligands to three individual BET-family BDs (20). These ligands, a number of which have subnanomolar affinities, exhibited greater selectivity than known inhibitors. Crystal structures of 13 distinct peptide–bromodomain complexes revealed highly diverse structures and binding modes among the cyclic peptide ligands. These data demonstrate not only that de novo cyclic peptide libraries contain a wide range of structural motifs but also that such ligands are capable of binding through multiple, entirely divergent modes to a single pocket.

## Results

### Identification of High-Affinity Cyclic Peptide Ligands to BRD3-BD1.

To identify de novo cyclic peptide ligands to BET-family bromodomains, we initially carried out an mRNA display-based RaPID screen against the N-terminal BD of BRD3 (BRD3-BD1). The peptide library for this screen was biased toward substrate-competitive peptides by genetic code reprogramming to replace methionine with acetyllysine at the AUG codon (21). To ensure that at least one AcK was included in each peptide, we designed an “AcK-focused” library with three to seven degenerate codons flanking each side of a fixed AcK position (*SI Appendix*, Fig. S2*B*). The start codon was reprogrammed to replace *N*-formylmethionine with *N*-chloroacetyltryptophan to produce macrocyclic peptides through spontaneous reaction with the thiol side chain of a downstream cysteine residue (*SI Appendix*, Fig. S2*B*).

**Fig. 1.**
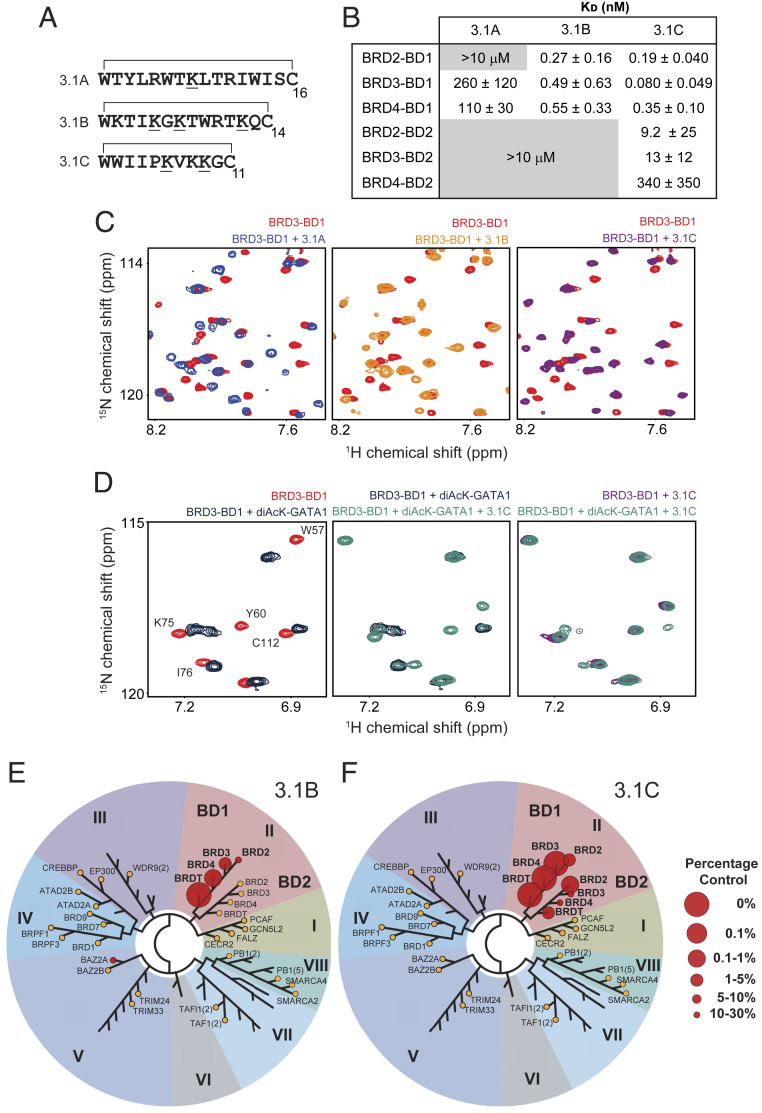
RaPID selection yields cyclic peptides with high affinity and specificity for BRD3-BD1. (*A*) Sequences of three peptides selected against BRD3-BD1. AcKs are underlined and the residues that form the macrocycle are indicated. (*B*) Table of *K*_D_ values for the binding of each BET BD to the peptides **3.1A**, **3.1B**, and **3.1C**, measured by SPR. Affinities are given as geometric means and SDs of two to five measurements. Gray boxed values indicate combinations for which no reproducible signals were observed in SPR experiments at the concentrations used. (*C*) Sections of ^15^N-HSQC spectra of BRD3-BD1 either alone (red) or in the presence of one molar equivalent of **3.1A** (*Left*; blue), one molar equivalent of **3.1B** (*Middle*; orange), or two molar equivalents of **3.1C** (*Right*; purple). ppm, parts per million. (*D*, *Left*) Selected region of ^15^N-HSQC spectra of BRD3-BD1 alone (red) and in the presence of six molar equivalents of a diacetylated GATA1 peptide (dark blue; GATA1 peptide, residues 302 to 316 and bearing acetylation marks on Lys312 and Lys314). (*D*, *Middle*) Selected region of an ^15^N-HSQC of BRD3-BD1 following the addition of two molar equivalents of **3.1C** to the BRD3-BD1–diacetylated GATA1 complex (*Left*). (*D*, *Right*) Overlay of the final spectrum obtained (*Middle*) with the same region of an ^15^N-HSQC of the BRD3-BD1–**3.1C** complex formed in the absence of GATA1. (*E*) TREEspot phylogenetic tree showing binding of 1 µM **3.1B** to 32 diverse human BDs in a BROMO*scan* competition binding assay. Larger spot size and lower percentage control reflect stronger affinity. BDs that were tested but showed no interaction are shown as orange circles and labeled. Unlabeled nodes represent BDs that were not tested. Their identity can be seen in *SI Appendix*, Fig. S5. The BET family are family II. (*F*) TREEspot phylogenetic tree showing binding of 1 µM **3.1C** to 32 diverse human BDs in a BROMO*scan* competition binding assay. Larger spot size reflects stronger affinity. BDs that were tested but showed no interaction are shown as orange circles and labeled. Unlabeled nodes represent BDs that were not tested. Their identity can be seen in *SI Appendix*, Fig. S6. The BET family are family II.

The resulting library of >10^12^ cyclic peptides was panned against biotinylated BRD3-BD1 immobilized on streptavidin beads. Following five rounds of selection, the enriched pools generated from the final three rounds were then sequenced, demonstrating a high degree of sequence enrichment (Dataset S1). As designed, the peptides identified each contained at least one AcK at the fixed central position but, interestingly, many also contained one or more additional AcK residues (Dataset S1). Notably, several biological targets of BET BDs also bear more than one AcK, with similar spacing to that observed in our selection data (11, 22). Sequence alignments of the top 500 sequences allowed us to divide the peptides into several families with distinct consensus sequences (Dataset S2). We selected the top two hits (members of BRD3-BD1 family 1 and BRD3-BD1 family 2, respectively, indicated in *SI Appendix*, Fig. S3 *A* and *B*) as well as the shortest peptide in the top 50 (the seventh hit and a member of the most abundant family, BRD3-BD1 family 3, which accounts for 28% of total sequencing reads recovered for the top 500 peptides; *SI Appendix*, Fig. S3*C*) for detailed structural and functional studies (Fig. 1*A*). These peptides were designated **3.1A**, **3.1B**, and **3.1C**, and were each synthesized using Fmoc-strategy solid-phase peptide synthesis and cyclized via thioether formation.

### RaPID-Selected Peptides against BRD3-BD1 Display Extremely High Specificity for BD1 Domains.

We next assessed the ability of each of the three lead peptides to bind BRD3-BD1. Surface plasmon resonance (SPR) studies demonstrated that these cyclic peptides exhibited very high affinity for this domain (Fig. 1*B* and *SI Appendix*, Fig. S4*A*). To assess selectivity for other BET BDs, we used SPR to determine the binding affinity of each peptide toward the remaining BDs from BRD2, BRD3, and BRD4 (Fig. 1*B*). All three peptides exhibited marked selectivity for BD1 domains, with observed binding affinities between 25 and >4,000 times tighter than those for BD2 domains. Among BD1 domains, peptides **3.1B** and **3.1C** displayed little selectivity, with affinities of each peptide for BRD2-BD1 and BRD4-BD1 within a factor of 2 to 4 of their affinity for BRD3-BD1, the domain against which they were selected. Peptide **3.1A**, in contrast, demonstrated some selectivity within the BD1 domains as well, displaying similar affinities for BRD3-BD1 and BRD4-BD1, but no observable interaction with BRD2-BD1.

We next used NMR to probe the interactions made by each of these peptides with BD1- and BD2-type BDs. ^15^N-heteronuclear single-quantum coherence (HSQC) spectra of BRD3-BD1 alone and in the presence of one molar equivalent of **3.1A**, **3.1B**, or **3.1C** showed significant chemical shift perturbations (CSPs) for a sizeable subset of signals (Fig. 1*C* and *SI Appendix*, Fig. S4 *B*–*E*), consistent with the SPR data. Spectra of the complexes had narrow lineshapes, consistent with the formation of complexes that are well-ordered, stable, and monodisperse. HSQC titrations with other BD–peptide combinations also corroborated the SPR data. In some cases, selective CSPs were observed for combinations that did not show binding in our SPR experiments (e.g., BRD2-BD1 with **3.1A** and BRD2-BD2 with **3.1B**; *SI Appendix*, Fig. S4*G*). The signals for the titration of BRD2-BD2 with **3.1B** were observed to be in fast exchange throughout the titrations, indicating significantly weaker interactions. Fitting the CSP data for the titration of BRD2-BD2 with **3.1B** to a simple 1:1 binding model yielded a binding affinity of ∼300 µM (*SI Appendix*, Fig. S4*G*), which corresponds to a remarkable selectivity of up to 10^6^-fold for the binding of **3.1B** to BD1 domains. Titration of BRD4-BD2 with peptide **3.1A** yielded no significant CSPs (*SI Appendix*, Fig. S4*H*), concordant with the SPR measurements for the same combination. Given the ability of NMR to detect interactions with *K*_D_ values as weak as ∼1 mM, these data demonstrate that **3.1A** binds BRD3-BD1 and BRD4-BD1 with a selectivity of ∼10,000-fold over BRD4-BD2.

Comparison of the CSPs observed for the BRD3-BD1 complexes (*SI Appendix*, Fig. S4 *C*–*E*) with the crystal structure of the same BD bound to the diacetylated substrate peptide from GATA1 (*SI Appendix*, Fig. S4*F*) indicated that the cyclic peptides all bind to the canonical AcK-binding pocket (22). To confirm this observation, we recorded an ^15^N-HSQC of BRD3-BD1 bound to a diacetylated GATA1 peptide and then further titrated this complex with **3.1C**. Signals that were diagnostic of GATA1 binding (e.g., W57, Y60) shift upon the addition of **3.1C** until they reach, following the addition of one molar equivalent of the cyclic peptide, the same positions observed for the BRD3-BD1–**3.1C** complex (Fig. 1*D*). These data confirm that the peptides compete directly with natural BRD3-BD1 partners for binding to the AcK pocket.

To determine the selectivity of our peptides across a diverse range of human bromodomain proteins, we subjected **3.1B** and **3.1C** to the standardized commercial BROMO*scan* assay (BromoMAX, performed by Eurofins DiscoverX), which profiles the relative affinity of 32 bromodomains for each compound. The two peptides showed extremely high selectivity for BET-family BDs, with almost no binding observed to other BD families at a peptide concentration of 1 μM (Fig. 1 *E* and *F*, full data in Dataset S3, and detailed TREEspot diagrams in *SI Appendix*, Figs. S5 and S6). The assay also allowed us to assess binding to the two BDs of BRDT, the fourth BET-family protein, which behaved comparable to the other BET proteins with regard to BD1 vs. BD2 binding to each peptide. Furthermore, the binding profile within the BET family precisely recapitulated that seen in our SPR data, emphasizing the high selectivity of **3.1B** for BD1 domains.

### Structures of 3.1C Bound to BET BDs Reveal a Highly Ordered Peptide and the Selection of a Native-Like AcK–BD Interaction.

**Fig. 2.**
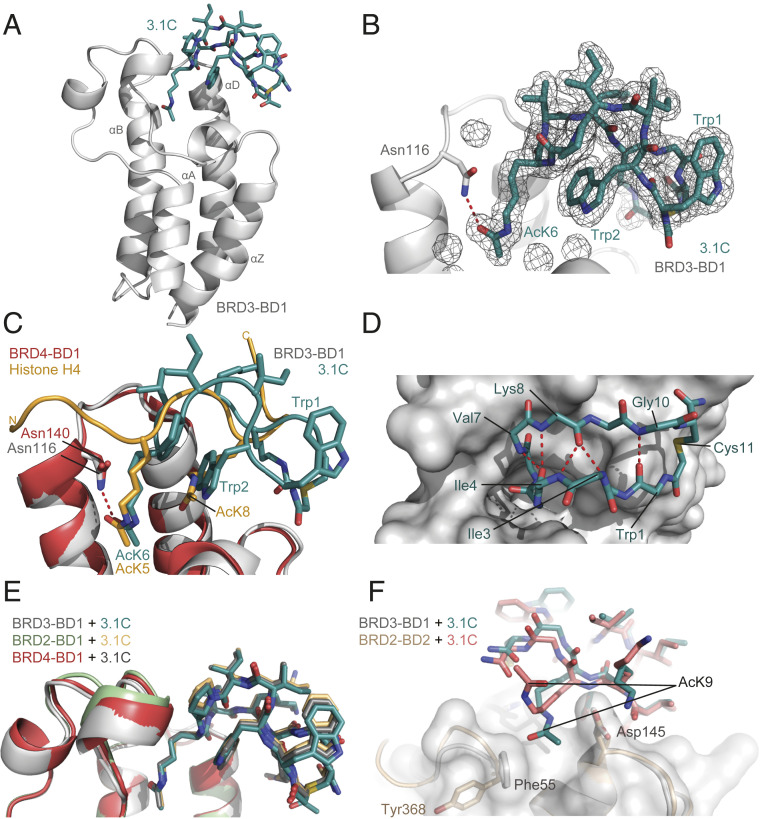
**3.1C** forms an extended beta structure that inserts an AcK into the canonical binding pocket of BRD3-BD1. (*A*) Ribbon representation of the X-ray crystal structure of the BRD3-BD1–**3.1C** complex (1.9-Å resolution, PDB ID code 6U4A). The peptide is shown in teal and the BD is shown in gray. (*B*) F_o_ − F_c_ map showing the electron density for **3.1C**, with BRD3-BD1 shown as a cartoon representation. (*C*) Overlay of the structure of BRD3-BD1 (gray) bound to **3.1C** (teal) and of BRD4-BD1 (coral red) bound to a diacetylated histone H4(1–12) (yellow) peptide (PDB ID code 3UVW) (25). The side-chain conformation of the AcK residues in the binding pocket is very similar. (*D*) Internal hydrogen bonding in the backbone of **3.1C**. Hydrogen bonds are indicated as red dashed lines. (*E*) Overlay of the X-ray crystal structures of BRD3-BD1 (gray, teal), BRD2-BD1 (pale green, light orange; 2.3-Å resolution, PDB ID code 6U61), and BRD4-BD1 (coral red, dark gray; 1.7-Å resolution, PDB ID code 6U6K), each in complex with **3.1C**. Structures are overlaid with the heavy atoms of the BDs. (*F*) Structure of **3.1C** bound to BRD2-BD2 (wheat, salmon; 1.5-Å resolution, PDB ID code 6U71) overlaid with the structure of **3.1C** bound to BRD3-BD1 (gray, teal).

We next determined the X-ray crystal structure of BRD3-BD1 bound to **3.1C** (1.9-Å resolution, Protein Data Bank [PDB] ID code 6U4A; Fig. 2 *A* and *B* and *SI Appendix*, Table S1). The conformation of the BD in the structure closely resembles that reported previously for the same domain bound to the archetypal pan-BD inhibitor JQ1 (PDB ID code 3S91; rmsd over ordered Cα backbone 0.3 Å), as well as that observed in multiple structures of BRD2-BD1 and BRD4-BD1 (12, 23, 24). Strikingly, one of the two AcK residues of the peptide (**3.1C**–AcK6) is inserted into the canonical AcK pocket and displays a side-chain conformation that closely resembles that observed for natural BD–AcK complexes; Fig. 2*C* shows an overlay of our structure with that of BRD4-BD1 bound to a diacetylated histone H4(1–12) peptide (PDB ID code 3UVW), a natural BET BD substrate, for comparison (25). The previously recognized “WPF shelf” adjacent to the AcK-binding pocket—which is occupied by a second AcK in the BRD4-BD1–histone H4 structure—is occupied by the side chain of Trp2 in our structure, and this side chain, together with the *N*-acetyl moiety of Trp1, sandwiches Trp57 of BRD3-BD1 (*SI Appendix*, Fig. S7*A*) (25). Instead of occupying this WPF shelf, the second AcK residue, AcK9, extends into an adjacent groove (*SI Appendix*, Fig. S7*B*).

Five intermolecular hydrogen bonds are formed in the complex (including the BRD3-BD1 Asn116 and **3.1C** AcK6 bond characteristic of bromodomain–AcK interactions; Fig. 2*B*) and 1,100 Å^2^ of surface area (across both molecules) is buried. This is low compared with known protein–protein complexes with the same affinity (1,500 to 2,500 Å^2^ is typical), pointing to the high binding efficiency that is accessible from cyclic peptide libraries of this type (25). Another notable feature of the structure is the high degree of internal hydrogen bonding observed for the peptide. The carbonyl group of Ile4 forms a bifurcated hydrogen bond with the amide protons of Val7 and Lys8, the Lys8 carbonyl is similarly hydrogen-bonded to the amide protons of Ile3 and Ile4, and the carbonyl of Trp1 forms a hydrogen bond with the amide of Gly10 (Fig. 2*D*).

We were also able to determine the structure of **3.1C** bound to BRD2-BD1 (2.3-Å resolution, PDB ID code 6U61; *SI Appendix*, Fig. S7*C* and Table S1) and bound to BRD4-BD1 (1.7-Å resolution, PDB ID code 6U6K; *SI Appendix*, Fig. S7*D* and Table S2). The structures overlay very closely with the BRD3-BD1–**3.1C** structure, with the peptide taking up an essentially identical conformation (Fig. 2*E*). All of the residues in all three structures that contact **3.1C** are completely conserved, consistent with the very similar affinities observed by SPR.

For comparison with the BD1-bound structures, we determined the crystal structure of **3.1C** bound to BRD2-BD2 (1.5-Å resolution, PDB ID code 6U71; *SI Appendix*, Table S2) to examine the structural basis for the selectivity of this peptide for BD1 domains (Fig. 2*F* and *SI Appendix*, Fig. S7*E*). Overall, the peptide backbone itself adopts the same conformation and all intermolecular contacts in the AcK-binding region are also conserved. Minor differences are a 180° flip of the Trp1 side chain (*SI Appendix*, Fig. S7*F*) and a change in the nature of the contacts made between AcK9 and the C-terminal part of helix αZ and N-terminal part of helix αD in the BD, which is distal to the main AcK pocket (a region less conserved between BD1 and BD2 domains). In the structures with BD1 domains, this AcK lies within a channel formed between conserved Phe (in the loop following αZ) and Asp (αD) residues. A tyrosine residue replaces the Phe in BD2 domains and is positioned in the opposite direction to the Asp, leading to a loss of this groove. As a result, AcK9 is oriented toward the solvent in our structure of the peptide with BRD2-BD2 (Fig. 2*F* and *SI Appendix*, Fig. S7*G*). This difference likely contributes to the significant differences in affinity for the two domains, illustrating the power of such peptides to achieve specificity by probing surfaces beyond the canonical binding pocket.

### 3.1B Is a β-Hairpin That Forms a Multivalent Interaction with BRD4-BD1 through Multiple AcK-Mediated Interactions.

**Fig. 3.**
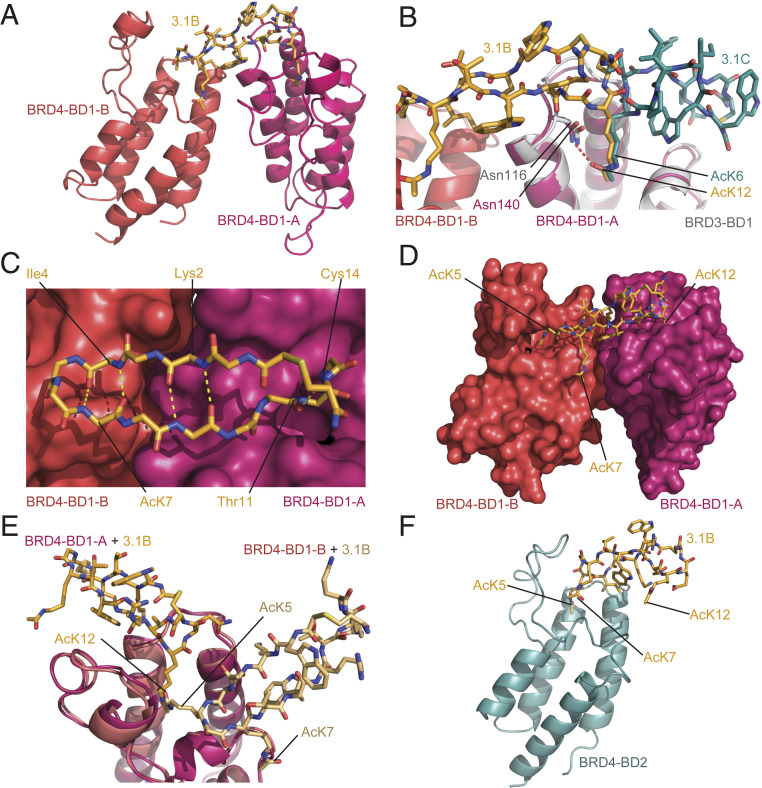
**3.1B** binds selectively to two BD1 domains through AcK-mediated interactions. (*A*) Ribbon diagram of the BRD4-BD1–**3.1B** X-ray crystal structure (1.9-Å resolution, PDB ID code 6U74). The peptide is shown in orange and the two BDs bound to the peptide are shown in pink (BRD4-BD1-A) and coral red (BRD4-BD1-B). (*B*) Comparison of the binding modes of **3.1B** (orange) and **3.1C** (teal; bound to BRD3-BD1, gray). The two peptides insert an AcK into the AcK-binding pocket in identical conformations but otherwise use completely distinct binding modes. (*C*) Internal hydrogen bonding of **3.1B**. Hydrogen bonds are indicated as yellow dashed lines. (*D*) Positions of the three AcK residues in the **3.1B** complex. AcK5 is partially buried in the AcK binding site of the additional BD (BRD4-BD1-B; coral red), whereas AcK7 lies on the surface of that domain. (*E*) Comparison of the angle at which AcK12 and AcK5 enter the binding pocket in the two BD subunits in the 2:1 complex. A copy of the BRD4-BD1-B (coral red) has been overlaid with BRD4-BD1-A (pink) and a copy of **3.1B** (pale orange) moved along with it. (*F*) Ribbon/stick representation of the X-ray crystal structure of the BRD4-BD2–**3.1B** complex (2.6-Å resolution, PDB ID code 6U6L). The peptide is shown in orange and the BD is shown in blue.

We next determined the X-ray crystal structure of BRD4-BD1 bound to **3.1B** (1.9-Å resolution, PDB ID code 6U74; Fig. 3*A* and *SI Appendix*, Table S3). This peptide binds in a very different manner from **3.1C**, while sharing one common central feature: the binding of an AcK (AcK12) in the canonical AcK pocket (Fig. 3*B*). The peptide forms an ordered β-hairpin with a high degree of internal backbone hydrogen bonding (Fig. 3*C*). It also contains two additional AcK residues and the structure reveals that, remarkably, these two residues engage a second molecule of BRD4-BD1 (BRD4-BD1-B); AcK5 binds in the AcK-binding pocket and AcK7 contacts the surface of the additional BD (Fig. 3*D*). The angle at which AcK5 enters the binding pocket differs markedly from the canonical conformation observed for AcK12 (more diagonal than vertical, as depicted in Fig. 3*E*). Consequently, the AcK hydrogen bond to Asn140 that is characteristic of these complexes cannot form in the same way and is instead mediated by a solvent water molecule (*SI Appendix*, Fig. S8*A*). Some contacts are also made between the surfaces of the two BDs (Fig. 3*D*).

To assess the contributions made by each AcK to BD binding, we synthesized peptides in which each of these three residues was mutated in turn to Ala. Mutation of AcK7 reduced the affinity of **3.1B** for all three BD1 domains by fourfold to ∼2 to 5 nM (*SI Appendix*, Fig. S8 *B* and *C*), whereas mutation of AcK5 to Ala reduced binding ∼80-fold (*K*_D_ 29 to 35 nM). Mutation of AcK12 to Ala resulted in a complete loss of binding to any BD1 domain under our assay conditions, indicating that this more canonical AcK–pocket interaction provides the largest contribution to binding. Given this dominant contribution of the AcK12–pocket interaction over the other AcK interactions, we used size-exclusion chromatography coupled to multiangle laser light scattering (SEC-MALLS) to probe whether equimolar concentrations of the protein and peptide would drive 1:1 complex formation in solution or whether the 2:1 stoichiometry observed in our structure still dominates. Somewhat surprisingly, we established that this complex displays the same 2:1 stoichiometry in solution (*SI Appendix*, Fig. S8*D*), rather than a 1:1 complex (that would have allowed the AcK12 residue to bind in the pocket of each BD). For comparison, we measured the molecular mass of the BRD3-BD1–**3.1C** complex using the same approach (*SI Appendix*, Fig. S8*D*); this complex yielded a molecular mass of 17 kDa, in good agreement with the 1:1 stoichiometry observed in our X-ray structure.

Crystal structures of BRD4-BD1 bound to the AcK5→Ala (2.3-Å resolution, PDB ID code 6U72; *SI Appendix*, Fig. S8*E* and Table S3) or AcK7→Ala peptides (2.6-Å resolution, PDB ID code 6U8G; *SI Appendix*, Fig. S8*F* and Table S4) were also essentially unchanged from the 2:1 “wild-type” complex. Together, these data suggest that the formation of the ternary complex provides significant additional energetic benefit, underscoring the importance of BD–BD contacts and reminiscent of proteolysis targeting chimera–substrate interactions reported recently (25).

Finally, we were able to determine the structure of **3.1B** bound to BRD4-BD2, a complex with a much lower affinity than those observed for **3.1B** binding to BD1 domains (2.6-Å resolution, PDB ID code 6U6L; Fig. 3*F* and *SI Appendix*, Table S4). In contrast to our observations for peptide **3.1C**, where selectivity between BD1- and BD2-type domains appears to derive from contacts in a region distal to the AcK pocket, the **3.1B** peptide recognizes the BD2 domain in a very different manner. The peptide and BD form a 1:1 complex in which it is AcK5 that is bound in the pocket (cf. AcK12 in interactions with BD1 domains). In this case, the interaction surface area is much smaller than observed for the BRD4-BD1 structure, consistent with the substantially lower *K*_D_. Furthermore, CSPs observed upon addition of **3.1B** to ^15^N-labeled BRD2-BD2 are consistent with this binding mode (*SI Appendix*, Fig. S8 *G* and *H*), indicating that this peptide binds to all BD2 domains in the same manner. This difference again highlights the ability of RaPID-selected peptides to robustly distinguish BD1- from BD2-type domains.

### Cyclic Peptides Can Also Bind Selectively to BD2 Domains or Indiscriminately to All BET BDs with Nanomolar Affinity.

**Fig. 4.**
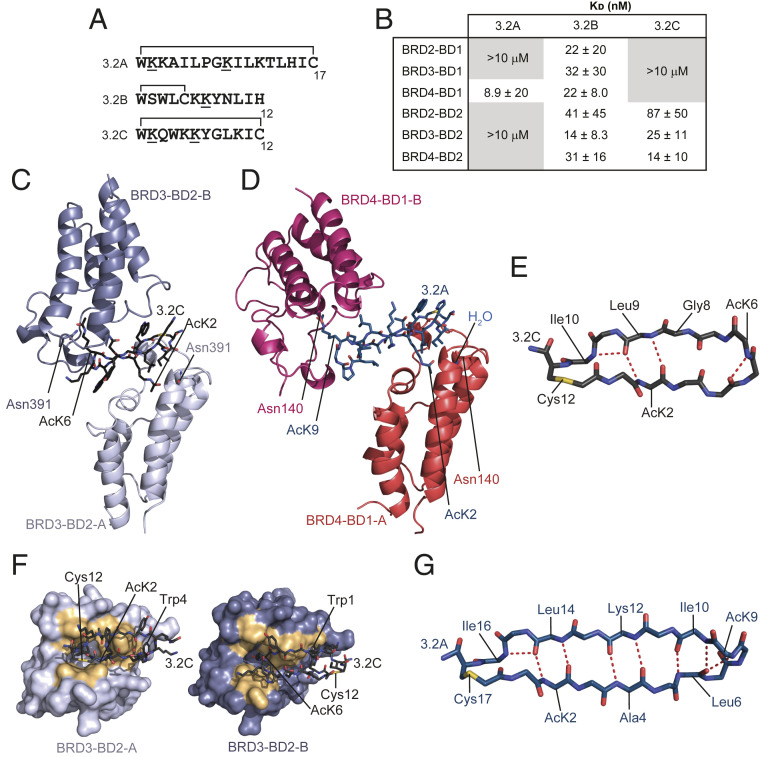
Screen against BRD3-BD2 discovers BD2-selective peptides that bind in a bivalent manner. (*A*) Sequences of three peptides selected against BRD3-BD2. Acetylated lysines are underlined and the residues that form the macrocycle are indicated. (*B*) Table of *K*_D_ values for the binding of each BET BD to the peptides **3.2A**, **3.2B**, and **3.2C**. Affinities are given as geometric means and SDs of two to five measurements. Gray boxed values indicate combinations for which no reproducible signals were observed in SPR experiments at the concentrations used. (*C*) Ribbon diagram of the BRD3-BD2–**3.2C** X-ray crystal structure (2.8-Å resolution, PDB ID code 6ULP). The two copies of the BD found in this 2:1 complex are shown in pale purple (BRD3-BD2-A) and dark purple (BRD3-BD2-B) and the peptide is shown in charcoal. AcK residues that enter the binding pocket are labeled. (*D*) Ribbon diagram of the BRD4-BD1–**3.2A** X-ray crystal structure (2.0-Å resolution, PDB ID code 6U8M). The two copies of the BD found in this 2:1 complex are shown in coral red (BRD4-BD1-A) and pink (BRD4-BD1-B) and the peptide is shown in navy. AcK residues that enter the binding pocket are labeled. (*E*) Backbone conformation of **3.2C** with the positions of AcK residues shown and hydrogen bonds indicated as red dashed lines. (*F*) Comparison of the contact surfaces (gold) made by **3.2C** in its interaction with BRD3-BD2-A and BRD3-BD2-B. Similar surfaces mediate both interactions. (*G*) Backbone of **3.2A** in the structure with BRD4-BD1. Internal hydrogen bonds are indicated with red dashed lines.

To contrast with our selection against a BD1 domain, we carried out an additional RaPID selection using the same library design but with BRD3-BD2 as the bait. Again, the top 500 sequences from each selection were divided into families based on sequence similarity (Dataset S4). Comparison of the peptides isolated from this selection with those selected in the BRD3-BD1 screen uncovered some families that were observed in both selections and some families that were unique to each screen. We selected the three most highly enriched peptides from the BRD3-BD2 screen (**3.2A**, **3.2B**, and **3.2C**; Fig. 4*A* and *SI Appendix*, Fig. S9) for synthesis and characterization. Interestingly, the most abundant peptide (**3.2A**, making up 3% of all sequence reads) was related to only one other peptide in the top 500 and not to any peptides from the BRD3-BD1 selection (*SI Appendix*, Fig. S9*A*). Likewise, the most abundant family identified in the BRD3-BD2 selection (BRD3-BD2 family 3, accounting for 53% of total sequencing reads recovered for the top 500 peptides, including **3.2C**) was not found in the BRD3-BD1 screen (*SI Appendix*, Fig. S9*C*). On the other hand, the family to which **3.2B** belongs was identified in the BRD3-BD1 screen with a relatively high abundance (BRD3-BD2 family 2, accounting for 8% of total sequencing reads recovered for the top 500 peptides from the BRD3-BD2 selection and 19% from the BRD3-BD1 selection; *SI Appendix*, Figs. S9*B* and S10).

In characterizing the BD-binding properties of the three peptides selected from the BRD3-BD2 selection, peptide **3.2A** proved to be problematic in SPR assays. Surprisingly, strong binding was not observed to BRD3-BD2, against which the peptide was selected in the SPR assay, although the peptide did reproducibly show an interaction with BRD4-BD1 (Fig. 4*B*). In ^15^N-HSQC experiments, BRD3-BD1 formed a well-defined complex with **3.2A**, with what we estimate to be micromolar affinity (*SI Appendix*, Fig. S11*A*). However, the addition of the peptide to BRD3-BD2 resulted in the disappearance of most signals, consistent with the formation of soluble aggregates (e.g., *SI Appendix*, Fig. S11*B*) and the poor behavior of the peptide in SPR experiments. Cyclic peptide **3.2B** showed strong binding but relatively little specificity, consistent with the abundance of related peptides found in the BRD3-BD1 selection; interactions with each of the six BDs had affinities in the range of ∼10 to 30 nM (Fig. 4*B*). In contrast, **3.2C** bound to the BD2 domains with affinities of 10 to 100 nM but did not bind strongly to any BD1 domain, consistent with selectivities of >10- to 100-fold between these domains (Fig. 4*B*).

We were able to obtain X-ray crystal structures of both **3.2C** bound to BRD3-BD2 (2.8-Å resolution, PDB ID code 6ULP; Fig. 4*C* and *SI Appendix*, Table S5) and **3.2A** bound to BRD4-BD1 (2.0-Å resolution, PDB ID code 6U8M; Fig. 4*D* and *SI Appendix*, Table S5). Like **3.1B**, both peptides bind two copies of the BD. In contrast to **3.1B**, however, almost no direct contacts were observed between the two BDs, suggesting that in these cases crystallization might have promoted the bivalent interaction. Consistent with this idea, SEC-MALLS indicated only weak formation of the 2:1 complex for BRD3-BD2–**3.2C** and exclusively a 1:1 complex for BRD4-BD1 bound to **3.2A** (*SI Appendix*, Fig. S11*C*). In the X-ray structure with BRD3-BD2, **3.2C** adopts an irregular β-hairpin stabilized by several internal backbone amide hydrogen bonds (Fig. 4*E*). The two AcK residues (AcK2 and AcK6) in the peptide each engage a BD via the AcK pocket although, in both cases, the *N*-acetyl moiety lies too far from the conserved Asn (Asn391) to form a direct hydrogen bond. It is likely that water-mediated interactions are formed, but the relatively low resolution of this structure (2.8 Å) prevented us from directly observing solvent water. The two peptide–BD interfaces bury 1,120 Å^2^ (BRD3-BD2-A) and 1,116 Å^2^ (BRD3-BD2-B) of surface area and the peptide contacts very similar surfaces in the two domains (Fig. 4*F*)—a surface that significantly overlaps with the contact surface for **3.1C** (Fig. 2*D*). Despite this overlap in contact surface, the only interaction that is completely conserved is the use of a Trp (Trp4 binding BD-A and Trp1 binding BD-B) to contact Trp322 on the BD.

For **3.2A**, essentially the same face of each of the two BDs is engaged as in the BRD3-BD2–**3.2C** complex, but the angle made between the long axes of the two BDs differs by ∼45°. In the **3.2A** structure, AcK9 forms a direct hydrogen bond with Asn140 in BRD4-BD1-A, whereas the AcK2–Asn140 interaction (with BRD4-BD1-B) is water-mediated (Fig. 4*D*). A prominent feature of **3.2A** is the high degree of internal backbone hydrogen bonding observed in the structure (Fig. 4*G*)—nine backbone amide hydrogen bonds form an extremely regular antiparallel β-sheet connected by two turns. Overall, these structures demonstrate that very different sets of amino acids can be used to contact the same protein surface.

### A Helical Scaffold Drives Binding of a Subset of RaPID-Selected Peptides to BD2 Bromodomains.

**Fig. 5.**
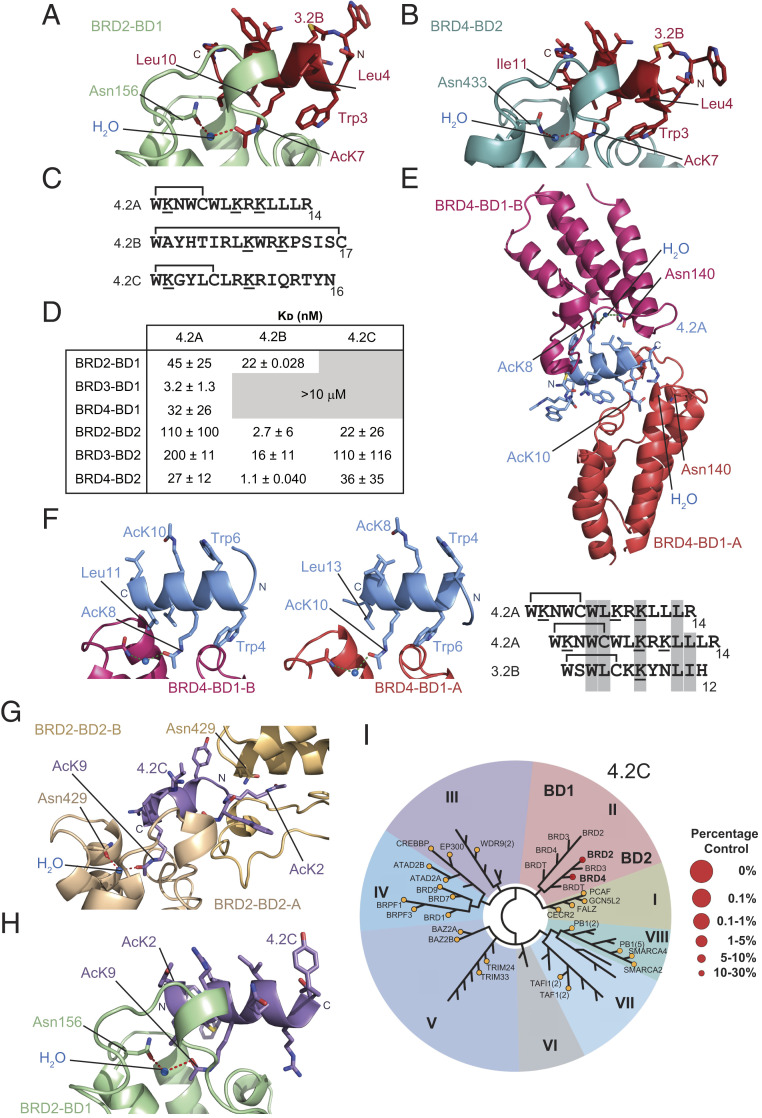
Lariat peptides derived from BD2-directed selections bind in an α-helical conformation. (*A*) Ribbon diagram of a portion of the BRD2-BD1–**3.2B** X-ray crystal structure (2.1-Å resolution, PDB ID code 6U8H). The peptide is shown in red and the BD is shown in pale green. An ordered water molecule that mediates the interaction between Asn156 of BRD2 and AcK8 of **3.2B** is shown as a blue sphere and hydrogen bonding is indicated. Side chains of **3.2B** are shown as sticks, as is Asn156 from the BD. (*B*) Ribbon diagram of a portion of the BRD4-BD2–**3.2B** X-ray crystal structure (2.5-Å resolution, PDB ID code 6U8I). The peptide is shown in red and the BD is shown in blue. An ordered water molecule that mediates the interaction between Asn433 of BRD2 and AcK8 of **3.2B** is shown as a blue sphere. Side chains of **3.2B** are shown as sticks, as is the side chain of Asn433. (*C*) Sequences of three peptides selected against BRD4-BD2. Acetylated lysines are underlined and the residues that form the macrocycle are indicated. (*D*) Table of *K*_D_ values for the binding of each BET BD to the peptides **4.2A**, **4.2B**, and **4.2C**. Affinities are given as geometric means and SDs of two to five measurements. Gray boxed values indicate combinations for which no reproducible signals were observed in SPR experiments at the concentrations used. (*E*) Ribbon representation of the X-ray crystal structure of BRD4-BD1–**4.2A** (2.2-Å resolution, PDB ID code 6ULV). The peptide is shown in light blue (with side chains as sticks) and the BD is shown in coral red (BRD4-BD1-A) and pink (BRD4-BD1-B). The AcK residues are labeled. (*F*) Comparison of the mechanism by which **4.2A** interacts with the two BD subunits of the BRD4-BD1–**4.2A** complex. (*F*, *Left*) Interaction with BRD4-BD1-A. (*F*, *Right*) Interaction with BRD4-BD1-B. The correspondence between AcK8 and AcK10, L11 and L13, and W4 and W6 can be seen. (*G*) Ribbon representation of a portion of the X-ray crystal structure of the BRD2-BD2–**4.2C** complex (2.8-Å resolution, PDB ID code 6ULT). The peptide is shown in purple (with side chains as sticks) and the BDs are shown in wheat (BRD2-BD2-A) and pale yellow (BRD2-BD2-B). The direction of the peptide helix is indicated. (*H*) Ribbon representation of a portion of the X-ray crystal structure of the BRD2-BD1–**4.2C** complex (2.7-Å resolution, PDB ID code 6ULQ). The peptide is shown in purple (with side chains as sticks) and the BD is shown in pale green. The direction of the peptide helix is indicated. (*I*) TREEspot phylogenetic tree showing binding of **4.2C** to the BDs of the BET family (represented as II) in a BROMO*scan* bromodomain competition binding assay performed using **4.2C** at 1 µM concentration. Larger spot size reflects stronger affinity. BDs that were tested but showed no interaction are shown as orange circles and labeled. Unlabeled nodes represent BDs that were not tested. Their identity can be seen in *SI Appendix*, Fig. S13. The BET family are family II.

Cyclic peptide **3.2B** differs from the other library members described above in that it displays a “lariat” topology in which cyclization involves an internal (rather than C-terminal) cysteine and a linear C-terminal tail (Fig. 4*A*). To assess whether such peptides have different structural features and binding modes, we determined X-ray crystal structures of **3.2B** bound to both BRD2-BD1 (2.1-Å resolution, PDB ID code 6U8H; Fig. 5*A* and *SI Appendix*, Table S6) and BRD4-BD2 (2.5-Å resolution, PDB ID code 6U8I; Fig. 5*B* and *SI Appendix*, Table S6). Strikingly, **3.2B** forms a single, well-defined α-helix in both structures; this helix contacts the AcK-binding surface of the two BDs with a very similar overall geometry and places its single AcK (AcK7) into the canonical binding pocket. It is notable that the AcK side chain enters the pocket at a similar diagonal angle to that observed for AcK5 in the peptide **3.1B** binding to the “second” copy of BRD4-BD1 (Fig. 3 *D* and *E*). Because of this insertion angle, the hydrogen bond made by **3.2B** with the pocket Asn is again mediated by a water molecule (Fig. 5 *A* and *B*). Residues Trp3, Leu4, Leu10, and Ile11 make hydrophobic contacts with the BDs and Trp3 also contacts the aliphatic part of the AcK7 side chain, most likely stabilizing the conformation of this residue in the binding pocket. Most of these hydrophobic contacts are conserved between the BRD2-BD1 and BRD4-BD2 structures, consistent with the similar affinities observed for the two interactions.

Concurrently, we performed a screen against BRD4-BD2, and measured the binding of the three most enriched sequences (**4.2A**, **4.2B**, and **4.2C**) to each of the BDs (Fig. 5 *C* and *D*, *SI Appendix*, Fig. S12, and Dataset S5). Sequence analysis revealed the most abundant peptide, **4.2A** (making up 21% of all sequencing reads and a member of the largest family isolated from this selection), to be related to **3.2B** (*SI Appendix*, Figs. S9*B* and S12*A*). **4.2A** bound to BRD4-BD2 with an affinity of 27 nM, which is ∼4- to 8-fold tighter than its interaction with other BD2 domains. Affinities of **4.2A** for the three BD1 domains were at least as high as that for BRD4-BD2; in fact, BRD3-BD1 bound 10-fold tighter (*K*_D_ 3.2 nM), a finding reflected in the presence of related sequences in all three of our selections. In contrast, **4.2B** and **4.2C** are members of smaller families that are detected only in the BRD4-BD2 selection (*SI Appendix*, Fig. S12 *B* and *C*). In line with this observation, **4.2B** and **4.2C** showed significant selectivity for BD2 domains over BD1 domains.

An X-ray crystal structure of the lariat peptide **4.2A** bound to BRD4-BD1 shows that it also forms an α-helix that in this case engages two molecules of BRD4-BD1 in a bivalent complex (2.2-Å resolution, PDB ID code 6ULV; Fig. 5*E* and *SI Appendix*, Table S7). SEC-MALLS for this complex indicated a 1:1 stoichiometry (*SI Appendix*, Fig. S11*D*), although a complex with BRD4-BD2 showed a weak 2:1 complex in solution. The peptide interacts with both BD molecules through the insertion of an AcK residue into the binding pocket, and the geometry of both AcKs closely resembles that observed for the related **3.2B** (featuring a bridging water molecule that mediates the interaction with Asn140; Fig. 5 *A* and *B*). Remarkably, **4.2A** presents a very similar surface to both BDs in the complex: The peptide sequence harbors two interdigitated Trp-φ-X_2_-AcK-X_2_-Leu-φ motifs (φ, hydrophobic amino acid) that are directed to opposite sides of the helix to engage the two BDs (Fig. 5*F*). This interaction surface—along with an additional hydrophobic contact residue observed in one of the **4.2A** interactions—is also conserved in **3.2B** (Fig. 5 *A* and *F* and *SI Appendix*, Fig. S12*D*). The fact that **4.2A** and **3.2B** belong to the same family of sequences, a family also found to be abundant in the BRD3-BD1 selection, indicates that this helical Trp-φ-X_2_-AcK-X_2_-Leu-φ motif is a highly favored BET BD-binding unit.

We also solved structures of another lariat-type peptide, **4.2C**, bound to a BD1 and a BD2 domain. Fig. 5*G* shows the structure in complex with BRD2-BD2 (2.8-Å resolution, PDB ID code 6ULT; *SI Appendix*, Table S8). The peptide is helical and bivalent, recruiting two molecules of BRD2-BD2. Its “primary” interaction has the **4.2C** helix binding in the same location as the other helical peptides but its long axis is ∼30° offset from that of the others. The second interaction is also mediated by an AcK–pocket interaction but with the helix unusually oriented “end on” to the pocket. In contrast, in the structure of **4.2C** bound to BRD2-BD1 (2.7-Å resolution, PDB ID code 6ULQ; Fig. 5*H* and *SI Appendix*, Table S8), the peptide takes up the same helical conformation but binds to only a single copy of BRD2-BD1 at the same location as **3.2B** and **4.2A** and with a similar overall geometry (Fig. 5*G*). Surprisingly, however, the helix in this case runs in the opposite direction from the other two structures. Together, these structures demonstrate that the class of lariat peptides that we have isolated is predisposed to take up helical conformations and that such peptides can bind BET BDs with affinities that are comparable to those observed for β-hairpin peptides.

Finally, to confirm the BD2 selectivity that we observed for **4.2C** by SPR and NMR, we subjected **4.2C** to the BROMO*scan* assay. Again, remarkable specificity was observed. No interaction was detected with any non-BET BD and, within the BET family, binding was only observed to the BD2s of BRD2 and BRD4, consistent with our SPR data (Fig. 5*I*, *SI Appendix*, Fig. S13, and Dataset S3).

### RaPID-Derived Cyclic Peptides Can Be Preorganized for Target Binding.

**Fig. 6.**
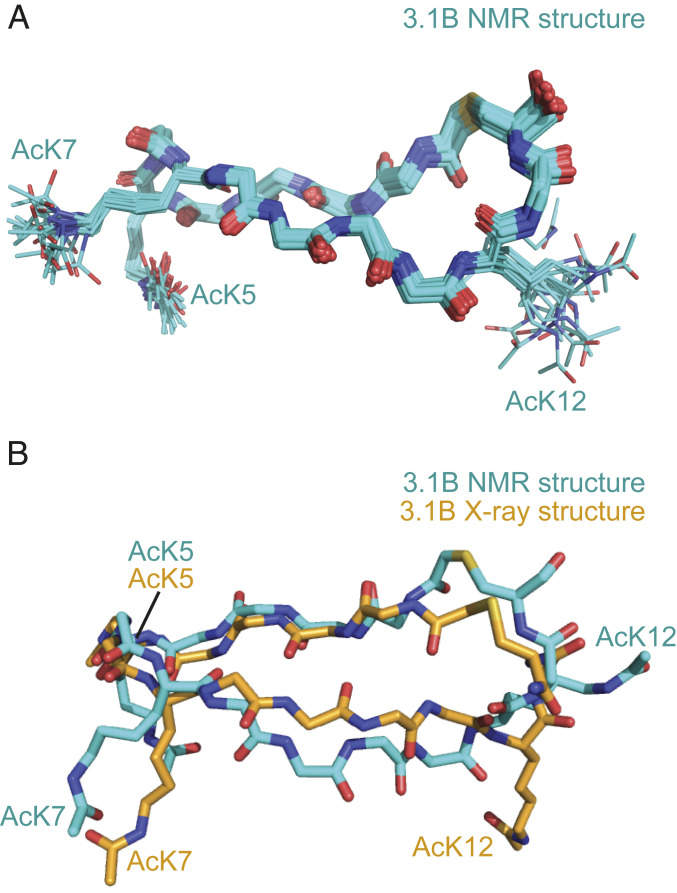
RaPID-derived cyclic peptides can be preorganized for target binding. (*A*) Solution structure of **3.1B**. An overlay (over backbone atoms) of the backbones of the 20 lowest-energy models is shown, with the side chains of the three acetyllysine residues shown as sticks. (*B*) Alignment (over Cα atoms) of a representative model from the solution structure of **3.1B** with the same peptide from the X-ray crystal structure in complex with BRD4-BD1.

A notable feature of many of the structures determined here is that the peptides take up very well ordered α- or β-type conformations with a significant number of intramolecular hydrogen bonds. We asked to what extent these conformations are preexisting before BD binding by using homonuclear ^1^H NMR spectroscopy to examine several of the peptides in isolation. A two-dimensional nuclear Overhauser effect (NOESY) spectrum of **3.1B** showed good dispersion of amide proton resonances and a significant number of medium- and long-range NOEs (*SI Appendix*, Fig. S14*A*), indicating that it is highly ordered in solution. Fig. 6*A* shows the solution structure of the peptide calculated from these NOE data (PDB ID code 6UXS; *SI Appendix*, Table S9). The structure is well-defined with the 20 lowest-energy conformers overlaying heavy backbone atoms with an rmsd of 0.19 Å. Comparison with the structure of **3.1B** in complex with BRD4-BD1 shows that residues Lys2 to Thr8 overlay closely whereas Trp1 and Trp9 to Cys14 take up a distinct, but well-ordered, conformation in the peptide alone (Fig. 6*B*). Thus, the peptide is highly ordered in isolation but unexpectedly appears to undergo a significant conformational change—from one well-ordered state to another—upon binding. The 1D ^1^H NMR spectra of **3.2A** and **3.1C** also display well-dispersed amide resonances, consistent with the formation of well-defined and stable structures (*SI Appendix*, Fig. S14*B*).

Finally, a NOESY of peptide **4.2C**, which adopts an α-helical structure when bound, displays several amide–amide NOEs, indicating helical propensity in the sequence (*SI Appendix*, Fig. S14*C*). However, the relatively small number of medium- and long-range NOEs suggests that the peptide exhibits significant conformational dynamics rather than a single well-defined conformation. Overall, these data suggest that the library contains numerous peptides that exhibit a significant degree of structural preorganization but that peptides with affinities in the nanomolar range are not all highly preordered.

## Discussion

### Cyclic Peptide Ligands Exhibit Substantial Structural and Binding-Mode Diversity.

Here we present a comparative investigation of de novo cyclic peptide ligands identified from multiple screening campaigns against a series of homologous targets. Our structural and biophysical analysis of the most highly enriched peptides from three separate RaPID screens demonstrates that cyclic peptides with a diverse range of structures can bind to even very highly conserved domains with high affinity and specificity. Strikingly, we observed very diverse structures and binding modes even for cyclic peptide ligands bound to the same pocket of the same BD target.

Alongside this diversity, some commonality was observed. The top 500 peptides in each selection could be parsed into one or two major sequence families (each comprising up to 15 to 50% of the total sequencing reads for the top 500 sequences) and several smaller families. Furthermore, some peptide families arose in all three selections while others were unique to each selection. The peptides that did not display a preference for the BET BDs, such as **3.1C**, **3.2B**, and **4.2A**, tended to belong to families with members in all three selections. Other peptides we investigated showed selectivity for either BD1 or BD2 domains and accordingly belonged to families that were either unique to the selections that they were isolated from or were only found in the selections performed against the same BD subtype. Furthermore, peptides that belong to the same family but were derived from different selections (**3.2B** and **4.2A**) adopted near-identical structures and binding modes. This result suggests that sequences in the same family most likely share the same binding mode, demonstrating the potential for assessing selectivity from the sequence alignments of selected peptide repertoires.

Despite the high diversity of the library and the high diversity of peptide structures observed, an AcK residue was found in all cases to bind in the canonical AcK-binding pocket, indicating that this is an “interaction hotspot” on the BDs and also flagging the privileged nature of the AcK–pocket interaction with its combination of hydrophobic interactions and a buried amide hydrogen bond. Beyond this similarity, however, the selected peptides adopt a wide range of highly ordered alpha- and beta-type structures that target different features of the BD surface (*SI Appendix*, Fig. S15). This observation contrasts sharply with the binding of BET BDs to native ligands. None of the 28 such structures feature significant secondary structure in the AcK peptide but rather are linear motifs with at most a single turn of α-helix (*SI Appendix*, Fig. S16).

It is notable that our cyclic peptides are without exception highly ordered in the structures of BD–peptide complexes, with only two or three residues in a single peptide not observed out of 15 X-ray crystal structures. Given that native BET BD interactions typically have affinities in the mid- to high-micromolar range, this observation suggests that the highest-affinity peptides for a given target are likely to be those for which much of the peptide takes up a well-defined conformation in the complex and makes numerous bonding interactions. A helical scaffold appears to be particularly favorable for BET BD binding and, strikingly, this property is independent of the direction of the helix but rather a consequence of the ability of a helix to juxtapose a set of side chains to engage the pocket region.

This compactness, high degree of order, and extensive internal hydrogen bonding in the peptides are clearly promoted by cyclization. Indeed, a subset of the peptides also demonstrates a high degree of preorganization in the unbound state. This preorganization is likely a strong contributor to the high affinities that are routinely observed in this and other naïve RaPID screens (26).

### RaPID-Derived Cyclic Peptides are the Highest-Affinity and Most Specific BET-Binding Molecules Discovered to Date.

BET bromodomain proteins have been therapeutic targets of substantial interest since the discovery that BRD4 is essential for disease maintenance in acute myeloid leukemia nearly 10 y ago (27). A range of long-running screening and medicinal chemistry campaigns carried out both in industry and in academia have delivered small-molecule BET BD binders with affinities of ∼1 to 50 nM (28). In fact, cyclic peptides based on native sequences containing AcK have been reported as bromodomain inhibitors, though with modest affinity (8 μM) (29). A number of these small molecules, including the prototype JQ1, have displayed high potency in murine cancer models (30). However, clinical trials to date have had mixed results (28), and it is possible that the moderate affinities of many of these molecules, combined with their generally low paralog specificity, are a contributing factor.

Using the RaPID system, we have identified peptides with diverse sequences and structures that bind to BET BDs with affinities of up to 10-fold stronger than the best small molecules. These peptides also display dramatically greater selectivity for BD1s over BD2s (and vice versa) than has been observed previously. The most selective small molecules have affinities that differ by a factor of ∼10 to 300 (28, 31–33); in comparison, we describe selectivities of up to ∼10,000-fold or more. Moreover, specificities of up to ∼10-fold were observed in some cases within the BD1 or BD2 subtypes. For example, binding of **3.1A** to BRD2-BD1 could not be observed reproducibly under our SPR conditions, whereas high-nanomolar binding was observed for BRD3-BD1 and BRD4-BD1 (Fig. 1*B*). Similarly, **4.2A** bound BRD3-BD1 30- and 40-fold tighter than BRD4-BD1 and BRD2-BD1, respectively (Fig. 5*D*). These data suggest that cyclic peptides might ultimately provide a route to achieving the intrasubfamily selectivity that has proved elusive with other classes of small molecules.

We note here that, rather than selectivity, the challenge with progressing cyclic peptides will be achieving robust cellular delivery. Although significant advances in cyclic peptide delivery are being made, it is likely that our peptides would need some modification before they could be applied as cellular tools (34). Efforts are currently ongoing to explore the cell permeability and stability of peptides identified in this work.

### Bivalent Complexes Offer Opportunities for More Sophisticated BET Targeting Strategies.

In many cases, our peptides were found to contain more than one AcK residue. While in some cases these multiple AcK residues bind to a single bromodomain, we also observed multiple instances of bivalent bromodomain binding. It is likely that this multivalency plays a role in the specificity of peptides for particular BDs, especially in cases where there are significant contacts between the two BDs—such as in the interactions between **3.1B** and BD1-type domains.

The observation of bivalent binding suggests a strategy for the design of peptides (or other molecules) that can simultaneously bind both BDs in a single BET molecule. Although our structures demonstrate the potential for bivalent interactions to occur with full-length BET proteins, we have not yet explored this possibility. There is, however, recent precedent for this activity in a report of bivalent BET BD inhibitors for ATAD2 and BRD4, which demonstrates the potential to induce full-length *cis* and *trans* BET–protein dimers (35, 36). Furthermore, Smith and coworkers recently exploited a similar bivalent strategy with covalent small-molecule fragments to derive selectivity for BRD4, suggesting that this approach may be generally applicable (37). Such pseudoternary complexes could well display higher specificity and affinity for one paralog.

This presence of multiple AcKs is a common feature of many bromodomain-binding acetylated transcription factors and histones (11, 22, 38, 39). Given this situation, it is possible that bivalent or multivalent binding of BD proteins is observed in the cell. To date, however, most structures containing native multi-AcK peptides have the AcK residues accommodated in a single BD (22, 40–42). There are, however, two structures that show binding of two BET BDs to a histone peptide through two closely spaced AcKs (*SI Appendix*, Fig. S16) (25, 40), as well as a third structure showing a diacetylated SIRT7 peptide binding two molecules of BRD4-BD1 (41). It remains to be seen whether BD multivalency is a common feature in biology.

Overall, our findings demonstrate that relatively small macrocyclic peptide ligands can adopt highly diverse conformations during binding to target proteins and greatly expand the catalog of de novo cyclic peptide–protein crystal structures solved to date. Whereas previous studies have demonstrated that such peptide ligands can adopt recognizable secondary structural features (e.g., β-helix or antiparallel β-sheet) when bound to specific binding pockets, our findings demonstrate that peptides with diverse structures can bind to the same pocket. This has profound implications for the design of cyclic peptide ligands, since optimal ligands may be structurally unrelated to known ligands in many cases. This finding likely also contributes to the success of RaPID and related strategies for isolating high-affinity ligands.

## Materials and Methods

Detailed descriptions of the materials, methods, and equipment used in this work, including protein expression and purification, RaPID screening, peptide synthesis, SPR, X-ray crystallography, NMR spectroscopy, and SEC-MALLs, are provided in *SI Appendix*, *Materials and Methods*.

## Supplementary Material

Supplementary File

Supplementary File

Supplementary File

Supplementary File

Supplementary File

Supplementary File

## Data Availability

All data are provided in the manuscript and supporting information. The coordinates and structure factors for all structures described in this paper have been deposited in the Protein Data Bank (PDB). The PDB IDs for all structures described in this study are as follows: 6U61 (BRD2-BD1 in complex with 3.1C), 6U4A (BRD3-BD1 in complex with 3.1C), 6U6K (BRD4-BD1 in complex with 3.1C), 6U71 (BRD2-BD2 in complex with 3.1C), 6U74 (BRD4-BD1 in complex with 3.1B), 6U6L (BRD4-BD2 in complex with 3.1B), 6U72 (BRD4-BD1 in complex with 3.1B_AcK5→Ala), 6U8G (BRD40BD1 in complex with 3.1B_AcK7→Ala, 6ULP (BRD3-BD2 in complex with 3.2C), 6U8M (BRD4-BD1 in complex with 3.2A), 6U8H (BRD2-BD1 in complex with 3.2B), 6U8I (BRD4-BD2 in complex with 3.2B), 6ULV (BRD4-BD1 in complex with 4.2A), 6ULQ (BRD2-BD1 in complex with 4.2C), and 6ULT (BRD2-BD2 in complex with 4.2C). The PDB ID codes for each structure have also been provided throughout the main text and are also listed in *SI Appendix*, Tables S1–S9.
