# Supplementary material for: Cyclic peptides can engage a single binding pocket through highly divergent modes
Source: Proc Natl Acad Sci U S A. 2020 Oct 12;117(43):26728–38. doi: 10.1073/pnas.2003086117 (PMC7604503; doi:10.1073/pnas.2003086117)
Supplement: Supplementary File [file pnas.2003086117.sapp.pdf]

PNAS
www.pnas.org

Supplementary Information for

Cyclic peptides can engage a single binding pocket through highly divergent modes.

Karishma Patel,[1]† Louise J. Walport,[2,3,4]†* James L Walshe,[1] Paul D. Solomon,[1] Jason K. K. Low,[1] Daniel H. Tran,[5] Kevork S. Mouradian,[1] Ana P. G. Silva,[1] Lorna Wilkinson-White,[6] Alexander Norman,[5] Charlotte Franck,[1, 5] Jacqueline M. Matthews,[1] J. Mitchell Guss,[1] Richard J. Payne,[5] Toby Passioura,[1,4,5,6] Hiroaki Suga,[4,*] Joel P. Mackay[1,*]

This PDF file includes:

Materials and Methods
Figures S1 to S17
Tables S1 to S9
SI References

SI Appendix Datasets provided in separate downloadable files:

Datasets S1-S5

www.pnas.org/cgi/doi/10.1073/pnas.2003086117

Materials and Methods

Protein expression and purification

The bromodomains from human BRD2 (BD1: 65-194; BD2: 347-455), BRD3 (BD1: 25-147; BD2: 307-419), and BRD4 (BD1: 42-168; BD2: 348-464) were cloned for bacterial expression into the pGEX-6P plasmid for expression as N-terminal GST-fusion proteins and into pQE80L-NAvi for expression as N-terminally His-tagged and biotinylated proteins.

Biotinylated bromodomains were prepared for SPR using the pQE80L-NAvi constructs. Proteins for crystallography and SEC-MALLS were produced using the pGEX-6P constructs. All bromodomain constructs, except BRD4-BD1, were transformed into competent BL21(DE3) *Escherichia coli* (*E. coli*) cells. LB expression cultures were inoculated with saturated starter cultures (1:100 dilution) grown from single colonies from fresh transformation plates. Expression cultures were incubated at 37 ºC, with shaking at 150 rpm, until cultures reached mid-log phase (OD600 of ~0.6-0.8). At this point, cultures were cooled to room temperature and supplemented with 250 µM IPTG (and 200 µM biotin for pQE80L-NAvi constructs). Expression cultures were transferred to 18 ºC for expression for a further ~20–24 h before harvesting via centrifugation. Biotinylated BRD4-BD1 was expressed using Rosetta2(DE3) *E. coli* cells. Autoinduction expression cultures were inoculated with a starter culture, as described above, and incubated at 37 ºC with shaking at 150 rpm. Upon reaching mid-log phase, cultures were cooled to room temperatures and supplemented with 200 µM biotin. Expression cultures were transferred to 18 ºC for expression for a further ~20–24 h before harvesting. All media were supplemented with the appropriate antibiotics. All cultures were harvested via centrifugation at 5000 ×g at 4 ºC for 25 min and cell pellets were stored at –20 ºC.

^{15}N-labelled proteins were expressed by transforming competent BL21(DE3) *E. coli* cells with pGEX-6P constructs. LB cultures were inoculated as described in the previous section. The cultures were harvested and washed in M9 minimal media salts once they reached mid-log phase. The cells were then resuspended in a volume of minimal media, containing $^{15}NH_4Cl$, half the volume of the original LB culture. The cultures were first transferred to 18 ºC for 45 min and then expression was induced using 250 µM IPTG. The cultures were incubated at 18 ºC for expression for a further ~20–24 h, with shaking at 150 rpm. All cultures were supplemented with antibiotics, harvested, and stored as described in the previous section.

Pellets from cells expressing proteins for SPR were lysed via sonication in a buffer composed of 50 mM Tris pH 8.0, 500 mM NaCl, 20 mM imidazole, 5 mM β-mercaptoethanol (β-ME), 0.1% Triton X-100, 1× cOmplete EDTA-free protease inhibitor, 10 µg/mL DNase I, 10 µg/mL RNase, and 100 µg/mL lysozyme. The lysate was clarified via centrifugation at 18,000 ×g for 30–60 min. The soluble fraction of the clarified lysate was subjected to immobilised nickel ion affinity chromatography using a 1-mL HisTrap column. The bound protein was eluted using a 20–250 mM imidazole gradient elution. The protein containing eluates from the affinity chromatography step were pooled and concentrated to a small volume. The concentrated sample was subjected to size exclusion chromatography (SEC) using a HiLoad 16/600 Superdex 75 column. Protein was eluted from the column using 50 mM Tris pH 8.0, 150 mM NaCl, and 1 mM DTT. Protein containing eluates were pooled and either aliquoted directly or concentrated before aliquoting. Aliquots were snap frozen in liquid nitrogen and stored at –80 ºC. Protein purification was analysed by SDS-PAGE and monitoring UV absorbance at 280 nm.

Cell pellets for protein for all other purposes were lysed in a buffer composed of 50 mM Tris pH 7.2, 500 mM NaCl, 5 mM β-mercaptoethanol (β-ME), 0.1% Triton X-100, 1× cOmplete EDTA-free protease inhibitor, 10 µg/mL DNase I, 10 µg/mL RNase, and 100 µg/mL lysozyme via sonication. Lysate was clarified via centrifugation at 18,000 ×g for 30–60 min. The soluble fraction of the clarified lysate was subjected to GSH-affinity chromatography using a 5-mL GSTrap column. Bound protein was step eluted with 50 mM Tris pH 7.2, 150 mM NaCl, 10 mM reduced glutathione, and 5 mM β-ME. Protein containing eluates were pooled and incubated with HRV-3C protease overnight

at 4 ºC to enable cleavage of the GST-tag. The pooled eluate was concentrated and subjected to size exclusion chromatography using a HiLoad 16/600 Superdex 75 column. The protein was eluted from the column in a buffer comprising 10 mM Tris pH 7.2, 100 mM NaCl, and 1 mM dithiothreitol (DTT). Fractions containing the desired protein were pooled and concentrated before aliquoting. ^{15}N-labelled protein was concentrated to 5–10 mg/mL and unlabelled protein was concentrated to 10–15 mg/mL. Protein aliquots were snap frozen in liquid nitrogen and stored at –80 ºC.

RaPID screening

DNA sequences containing a T7 polymerase binding site, ribosome binding site, variable length randomised peptide coding region containing a fixed central 'ATG' codon for incorporation of the fixed AcK, $(Gly\text{-}Ser)_3$, linker, 'TAG' stop codon and sequence for puromycin ligation were constructed by extensions and PCR from oligos purchased from Eurofins Genomics K.K. (Japan) to produce AcK-focused libraries. Library DNA –

TAATACGACTCACTATAGGGTTGAACTTTAAGTAGGAGATATATCCATG$(NNK)_{m=3-7}$ATG$(NNK)_{n=4-7}$TGTGGGTCTGGGTCTGGGTCTTAGGTAGGTAGGCGGAAA

DNA libraries were transcribed to mRNA using T4 RNA polymerase and mRNA libraries were ligated to a puromycin-PEG-DNA splint using T4 RNA ligase following standard reaction conditions. For the first selection round libraries were mixed in the following proportions: (m=3,n=4):(m=4,n=4):(m=4,n=5):(m=5,n=5):(m=5,n=6):(m=6,n=6):(m=6,n=7):(m=7,n=7) = 0.01425:0.45:10:10:7.5:7.5:7.5:7.5

For codon reprogramming, the 3,5-dinitrobenzyl ester of N^{ε}-acetyl-lysine was synthesised as previously described and aminoacylated onto $tRNA^{Asn}_{CAU}$ using dFx (2hr, RT, standard aminoacylation conditions) for incorporation into library peptides[1]. Peptides were initiated with *N*-(chloroacetoxy)-L-tryptophan (ClAc-L-Trp), which was aminoacylated via the cyanomethyl ester onto $tRNA^{fMet}_{CAU}$ using eFx (2hr, RT, standard aminoacylation conditions). Flexizymes were prepared as described previously[2].

RaPID screens were carried out as previously described[3]. Briefly, puromycin-ligated randomised mRNA libraries were *in vitro* translated (30 min, 37 °C then 12 min, 25 °C) using a custom transcription/translation mixture containing additional 12.5 µM ClAc-L-Trp-$tRNA^{fMet}_{CAU}$ and 25 µM AcK-$tRNA^{Asn}_{CAU}$ and lacking methionine and 10-formyl-5,6,7,8-tetrahydrofolic acid[3]. First-round translations were carried out on a 150-µL scale, subsequent rounds on a 5-µL scale. Following addition of 200 mM EDTA, pH 8.0 (15 µL) and reverse transcription with M-MLV RTase, RNase H minus (Promega), blocking buffer was added (50 mM HEPES, 150 mM NaCl, 2 mM DTT, 0.1% Tween-20, 0.2% (w/v) acetylated bovine serum albumin, pH 7.5) and libraries were incubated with magnetic streptavidin bead-immobilised bromodomain (Promega) (200 nM, 30 min). Following washing (156 µL ice-cold 50 mM HEPES, 150 mM NaCl, 2 mM DTT, 0.1% Tween-20, pH 7.5, 3 × 5 min), 400 µL PCR solution was added and retained peptide-mRNA/DNA hybrids were eluted from the beads by heating (95 °C, 5 min). Library enrichment was assessed by quantitative real-time PCR relative to standards and the input DNA library using primers T7g10M_F46 and CGS3-CH.R22. Enriched pools were amplified using the same primers and used as the input DNA for subsequent selection rounds.

T7g10M_F46 -TAATACGACTCACTATAGGGTTGAACTTTAAGTAGGAGATATATCC

CGS3-CH.R22 – TTTCCGCCTACCTACCTAAGAC

Following RaPID selections, double indexed libraries (Nextera XT indices) were prepared from recovered library DNA from rounds 3–5 and sequenced on a MiSeq platform (Illumina) using a v3

chip as single 151 cycle reads[3]. Each DNA sequence was converted to a peptide sequence and ranked by total read number (Dataset S1).

Peptide synthesis

Peptides were synthesised as C-terminal amides using standard fluorenylmethyloxycarbonyl (Fmoc)-strategy solid-phase chemistry using a Syro I automated synthesiser (Biotage) and NovaPEG Rink Amide resin (Novabiochem). Couplings were performed with HBTU/HOBt (1:1) and 6 equivalents of each amino acid. Double couplings were performed for arginine residues. Following the final amino acid coupling reaction, the Fmoc group was removed and resin incubated with N-(chloroacetoxy)succinimide (0.2 M in DMF, 1 h, RT). Resin was washed with DMF (5 times) and DCM (5 times) and dried *in vacuo*.

Linear peptides were cleaved from the resin by incubation (3 h, RT) with a trifluoroacetic acid (TFA) cleavage cocktail (TFA/2,2'-(ethylenedioxy)diethanethiol/triisopropyl silane/H_2O (92.5:2.5:2.5:2.5)) before filtration, concentration *in vacuo* and precipitation with ice-cold diethyl ether. Crude peptides were washed with diethyl ether (5 times), dried and resuspended in DMSO. The pH was raised to >8 using triethylamine to allow cyclisation. Following incubation (1 h, RT), peptides were re-acidified with TFA for purification.

Crude peptides were purified by reverse-phase high-performance liquid chromatography using a Chromolith Prep column (Merck) on a Prominence LC-20AP system (Shimadzu) (Solvent A: 0.1% TFA in H_2O, Solvent B: 0.1% TFA in acetonitrile) to >95% purity as determined by mass spectrometry (Fig. S17). Purified peptides were reconstituted in DMSO and concentrations determined from their absorbance at 280 nm in 5% DMSO using predicted extinction coefficients.

Surface plasmon resonance (SPR)

Measurements were conducted on a T200 or S200 (GE Healthcare) and data analysed using the Biacore Insight Evaluation Software. Experiments were performed at 4 °C in single cycle kinetics mode. Biotinylated BET bromodomains were immobilised on a CAP chip (GE Healthcare) with a target density of ~1000-1500 RU. 50 mM HEPES, 150 mM NaCl, 0.05% Tween-20, 0.1% DMSO, pH 7.5 was used as the running buffer. Between cycles the chip was regenerated following the manufacturer's protocol.

X-ray crystallography

Crystallisation of bromodomain-peptide complexes was performed using a sitting-drop vapour-diffusion technique. Purified bromodomains (10–15 mg/mL) were combined with ~1.5 molar equivalents of peptide and incubated on ice for at least 0.5 h. In situations where the bromodomain concentration was diluted below 5 mg/mL after addition of peptide, the bromodomain-peptide mixture was concentrated to bring the bromodomain concentration back to ~10 mg/mL. Initial crystallisation trials were performed using commercial 96-well crystallisation screens. Bromodomain-peptide mixtures were dispensed into MRC two-drop chamber, 96-well crystallisation plates using a Mosquito crystallisation robot and each condition was screened at a 1:1 or 2:1 protein to precipitant ratio (maintaining a final drop volume of 300 nL). In certain cases, crystallisation was performed without pre-mixing of the bromodomain and peptides by separately dispensing 100 nL of protein and peptide directly into the crystallisation plate prior to mixing with the precipitant. Where required, initials hits were optimised by gradient refinement of the original condition, scaling up drop sizes, and microcrystal seeding. All experiments were performed at 18 °C. Protein crystals generally took days to weeks to appear. Crystals were frozen by plunge-freezing in liquid nitrogen following cryoprotection with 10% glycerol in the mother liquid from which the crystals were grown.

X-ray diffraction data were collected from frozen crystals at the Australian Synchrotron using the Macromolecular Crystallography MX1 (bending magnet) and MX2 beamlines (microfocus) at 100 K and a wavelength of 0.9537 Å[4,5]. Data were integrated using XDS and were processed further using the CCP4i suite[6,7]. AIMLESS was used for indexing, scaling, and merging of the data and the initial phases were calculated by the molecular replacement program PhaserMR using existing x-ray structures of bromodomains as the molecular replacement models (PDB IDs 4UYF for BRD2-BD1, 3ONI for BRD2-BD2, 3S91 for BRD3-BD1, 3S92 for BRD3-BD2, 4LYI for BRD4-BD1, and 5UVV for BRD4-BD2)[8–12]. Manual model building was performed using COOT and refinement was performed by iterative rounds of manual building in COOT followed by refinement using Phenix[8,13]. The quality of the final model was validated with the wwPDB server and submitted to the PDB. Structure diagrams were generated using PyMol. Protein:peptide interfaces were analysed using PDBePISA. The data collection and refinement statistics for all structures described in this study are outlined in Supplementary Tables 2-16.

NMR spectroscopy

NMR samples of BET BDs were prepared at ~50-100 µM concentrations and were titrated with different molar equivalents of peptides. NMR spectra were acquired at 298 K using Bruker Avance III 600- or 800-MHz NMR spectrometers fitted with TCI probe heads and using standard pulse sequences from the Bruker library. TOPSPIN3 (Bruker) and NMRFAM-SPARKY were used for analysis of spectra[14]. Spectra were internally referenced to 10 μM 4,4-dimethyl-4-silapentane-1-sulfonic acid. Chemical shift perturbation experiments were performed by collecting ^{15}N-HSQC spectra of ^{15}N-labelled bromodomains before and after titration of unlabelled peptide into the samples. Interactions were assessed by monitoring the chemical shift perturbations induced by addition of the peptides to the bromodomains.

Chemical shift assignments were made for BRD3-BD1 using the standard triple resonance approach. Chemical Shift Perturbation (CSP) plots were generated using the following approach. A reference HSQC was recorded without the ligand present (ref HSQC), and then the ligand was added and a second HSQC (shift HSQC) recorded. The two HSQCs were peak picked using the APES algorithm in NMRFAM-Sparky to generate peak lists. The distance between each peak on the ref HSQC and all peaks on the shift HSQC was computed as follows:

$$dist = \sqrt{\left(\delta H_{ref} - \delta H_{shift}\right)^2 + 0.1 * \left(\delta N_{ref} - \delta N_{shift}\right)^2}$$

The minimum of the distances computed for each reference peak was taken as the CSP. Although this approach does not ensure every peak is correctly matched, it has the desirable property that the reported CSPs are guaranteed to be real and not an artefact of the algorithm, as the reported CSPs are a minimum of all possible CSP allocations.

The structure of **3.1B** was determined using NOESY, TOCSY and DQF-COSY spectra recorded at 5 °C. Spectra were analysed using CCPNMR Analysis and structures were calculated in CYANA3.98.5[15,16]. Cyclization was introduced according to the protocol provided at the CYANA wiki (http://www.cyana.org/wiki/). The topology for the acetyllysine residues was created manually. For structures run with hydrogen bond restraints, the hydrogen bonds introduced were (a) Trp9-HN to Lys2 O and (b) Ile4 HN to AcK7 O. The restraints were added following the CYANA wiki recommendations. The final ensemble was the lowest-energy set of 20 models from 1000 calculated models.

SEC-MALLS

Molecular weight calculations were carried out by subjecting samples (100 µL of 100-300 µM protein) to SEC (Superdex 75 10/300 Increase) on an Äkta system with inline multi-angle laser light (MALLS), UV absorbance, and refractive index (dRI) detectors. MALLS, UV, and dRI data were collected and analysed using the ASTRA software (Wyatt) and molecular weight determination was carried out according to the Debye-Zimm model. BSA (100 µL of 2 mg/mL) was run as a standard to calibrate align the MALLS, UV, and dRI signals. We estimate the uncertainty in the molecular weight determination from this system to be ±10%.

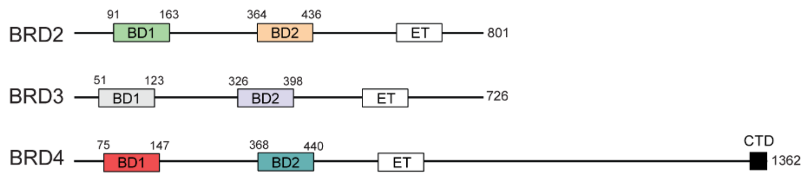

Figure S1. Domain architecture of the BET family and RaPID warhead library design. Domain topology of human BRD2, -3 and -4. BD1 and BD2 are bromodomains, ET is the extra-terminal domain, and CTD is the C-terminal domain found in BRD4. Residue ranges for the BDs are indicated.

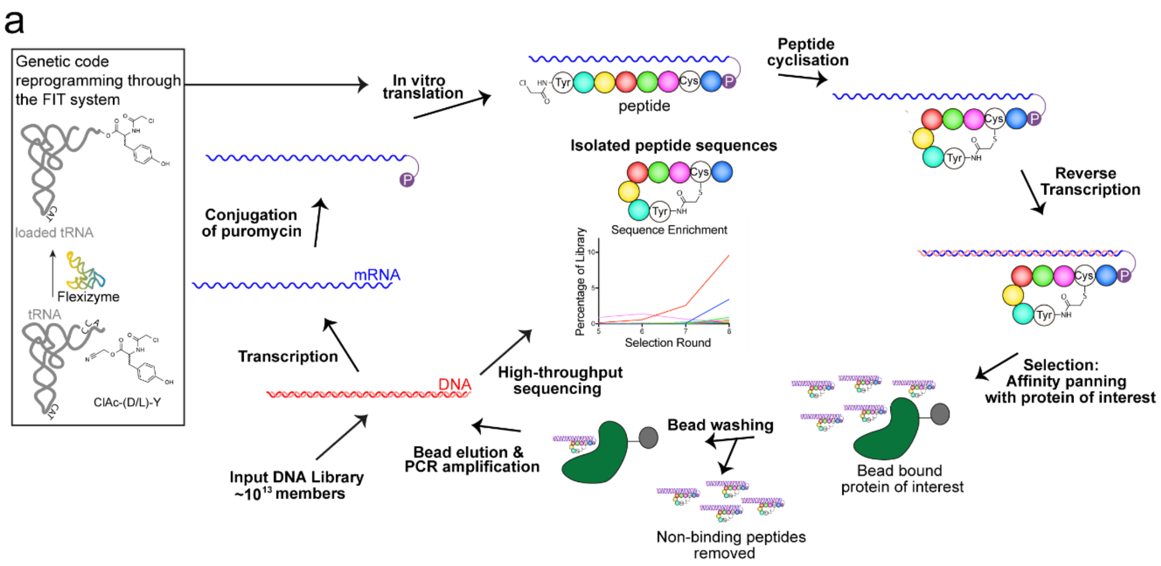

b

m = 3-7
n = 4-7

ATG(NNK)mATG(NNK)nTGTGGGTCTGGGTCTGGGTCTTAG

Peptide Sequence | Linker | Stop Codon

1st	3rd	2nd: U	2nd: C	2nd: A	2nd: G
U	U	Phe	Ser	Tyr	Cys
	C				
	A				
	G	Leu	Ser	Stop	Trp
C	U	Leu	Pro	His	Arg
	C				
	A				
	G	Leu	Pro	Gln	Arg
A	U	Ile	Thr	Asn	Ser
	C				
	A				
	G	**KAc**	Thr	Lys	Arg
G	U	Val	Ala	Asp	Gly
	C				
	A				
	G	Val	Ala	Glu	Gly

Initiator = ClAc-Trp

Figure S2. Overview of the RaPID system. *(A)* Schematic of the RaPID selection scheme. *(B)* Codon assignment used in the RaPID selections.

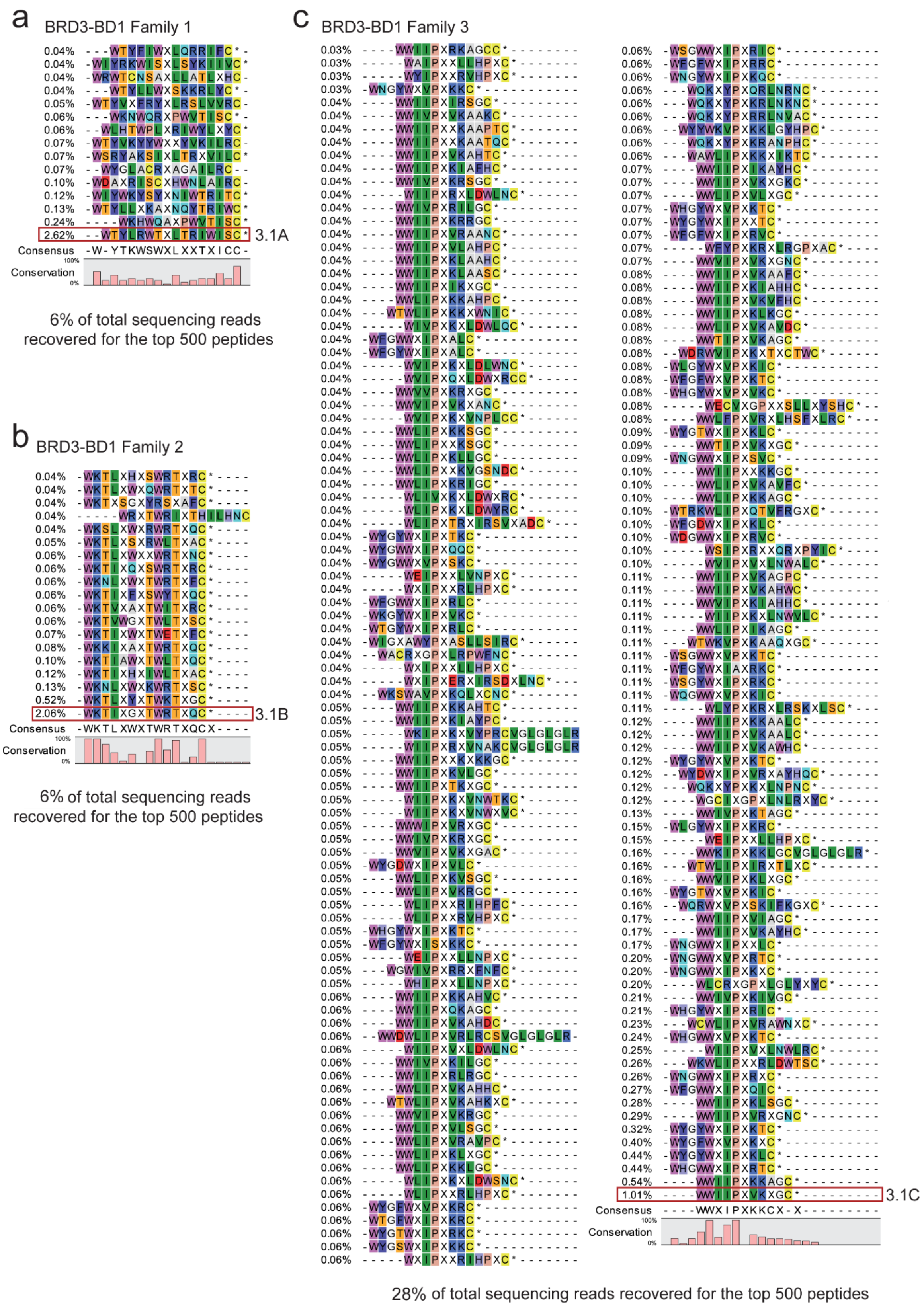
a
BRD3-BD1 Family 1
3.1A
Consensus
-W-YTKWSWXLXXTXICC-
Conservation
6% of total sequencing reads recovered for the top 500 peptides
b
BRD3-BD1 Family 2
3.1B
Consensus
-WKTLXWXTWRTXQCX----
Conservation
6% of total sequencing reads recovered for the top 500 peptides
c
BRD3-BD1 Family 3
3.1C
Consensus
----WWXIPXKKCX-X---------
Conservation
28% of total sequencing reads recovered for the top 500 peptides

Figure S3. Sequences of all peptides from the BRD3-BD1 selection that are members of the families to which **3.1A** *(A)*, **3.1B** *(B)*, and **3.1C** *(C)* belong. The percentages next to each peptide are the proportions of the total sequencing reads that were recovered for the peptide. The proportion of total sequencing reads recovered for the top 500 peptides is indicated below each family.

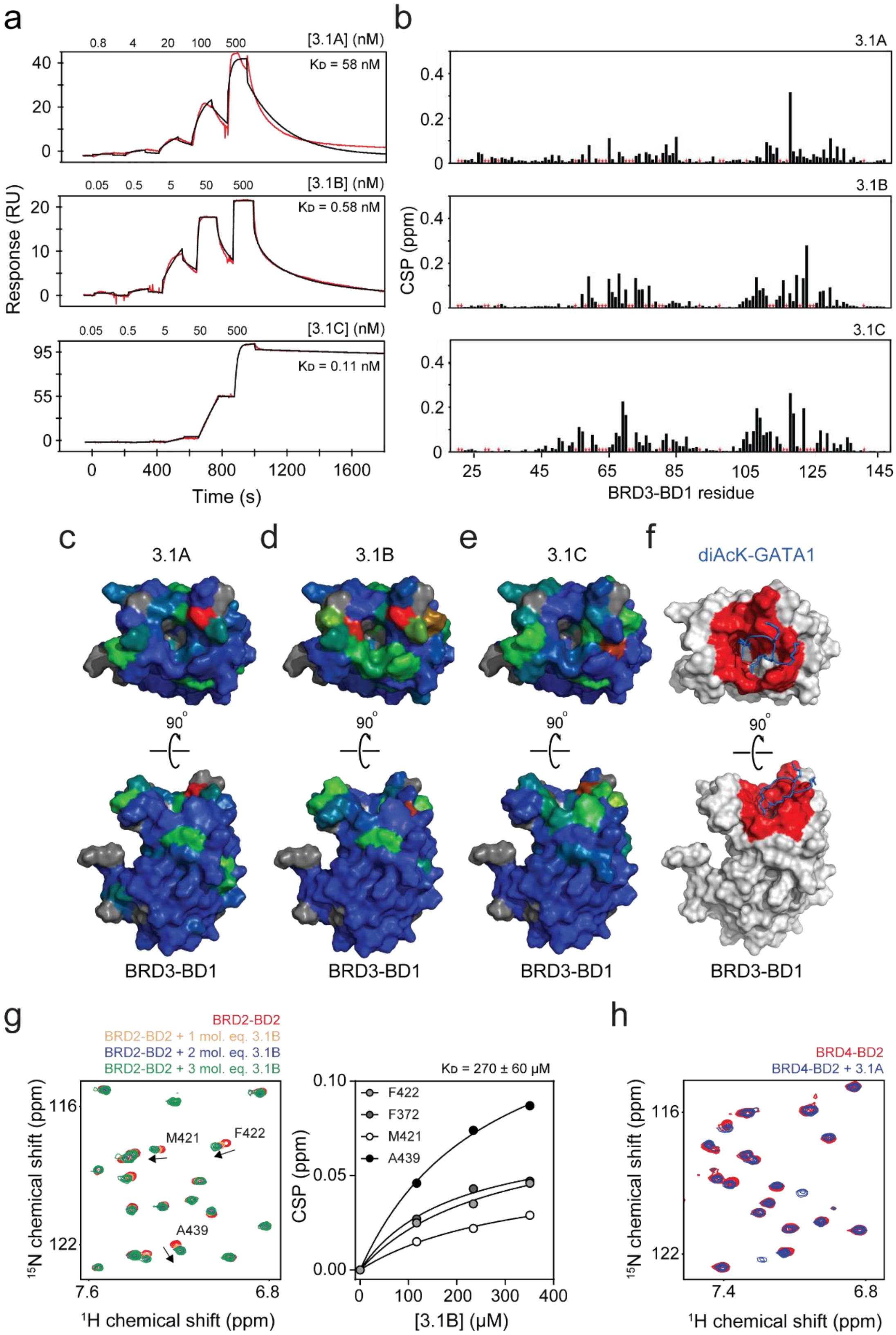
a
[3.1A] (nM)
0.8 4 20 100 500
KD = 58 nM
[3.1B] (nM)
0.05 0.5 5 50 500
KD = 0.58 nM
[3.1C] (nM)
0.05 0.5 5 50 500
KD = 0.11 nM
Response (RU)
Time (s)
b
3.1A
3.1B
3.1C
CSP (ppm)
BRD3-BD1 residue
c
3.1A
d
3.1B
e
3.1C
f
diAcK-GATA1
90°
BRD3-BD1
g
BRD2-BD2
BRD2-BD2 + 1 mol. eq. 3.1B
BRD2-BD2 + 2 mol. eq. 3.1B
BRD2-BD2 + 3 mol. eq. 3.1B
M421
F422
A439
15N chemical shift (ppm)
1H chemical shift (ppm)
KD = 270 ± 60 µM
F422
F372
M421
A439
CSP (ppm)
[3.1B] (µM)
h
BRD4-BD2
BRD4-BD2 + 3.1A
15N chemical shift (ppm)
1H chemical shift (ppm)

Figure S4. RaPID peptides bind BRD3-BD1 in the canonical AcK-binding pocket. *(A)* Representative SPR sensorgrams for BRD3-BD1 binding to **3.1A** (*upper panel*), **3.1B** (*middle panel*), and **3.1C** (*bottom panel*). *(B)* Chemical shift perturbations in the ^{15}N-HSQC of BRD3-BD1 upon formation of complexes with either **3.1A** (*upper panel*), or **3.1B** (*middle panel*), or **3.1C** (*lower panel*). The red stars indicate unassigned residues. *(C)* CSPs observed upon formation of the **3.1A** complex mapped onto the structure of BRD3-BD1 (PDB: 3S91)[9]. The degrees of CSP are displayed through a blue-green-red gradient: the blue portions of the structure do not undergo any CSPs and the red sections represent the residues that display the greatest CSPs. Grey regions indicate unassigned residues. *(D)* CSPs observed upon the **3.1B** complex mapped onto the structure of BRD3-BD1 (PDB: 3S91)[9]. The degrees of CSP are displayed through a blue-green-red gradient: the blue portions of the structure do not undergo any CSPs and the red sections represent the residues that display the greatest CSPs. Grey regions indicate unassigned residues. *(E)* CSPs from the **3.1C** complex are mapped onto the structure of BRD3-BD1 (PDB: 3S91)[9]. The degrees of CSP are displayed through a blue-green-red gradient: the blue portions of the structure do not undergo any CSPs and the red sections represent the residues that display the greatest CSPs. Grey regions indicate unassigned residues. *(F)* Structure of the BRD3-BD1 complex with a diacetylated-GATA1 peptide (2L5E, the peptide is shown in *blue* and the BD is shown in *grey*)[17], highlighting BD residues (*red*) that directly contact the peptide. *(G)* Overlaid section of ^{15}N-HSQC spectra of BRD2-BD2 alone (*red*) or in the presence of one (*yellow*), two (*blue*), or three (*green*) molar equivalents of **3.1B**. Signals that undergo the greatest CSPs in the titration are labelled in the protein alone spectrum. Binding curves were derived by tracking the combined ^{15}N/^{1}H chemical shift perturbations of the signals undergoing the greatest CSPs. The data were fitted to a simple 1:1 Langmuir binding isotherm using Graphpad. *(H)* Overlaid ^{15}N-HSQC spectra of BRD4-BD2 alone (*red*) or in the presence of one (*blue*) molar equivalent of **3.1A**.

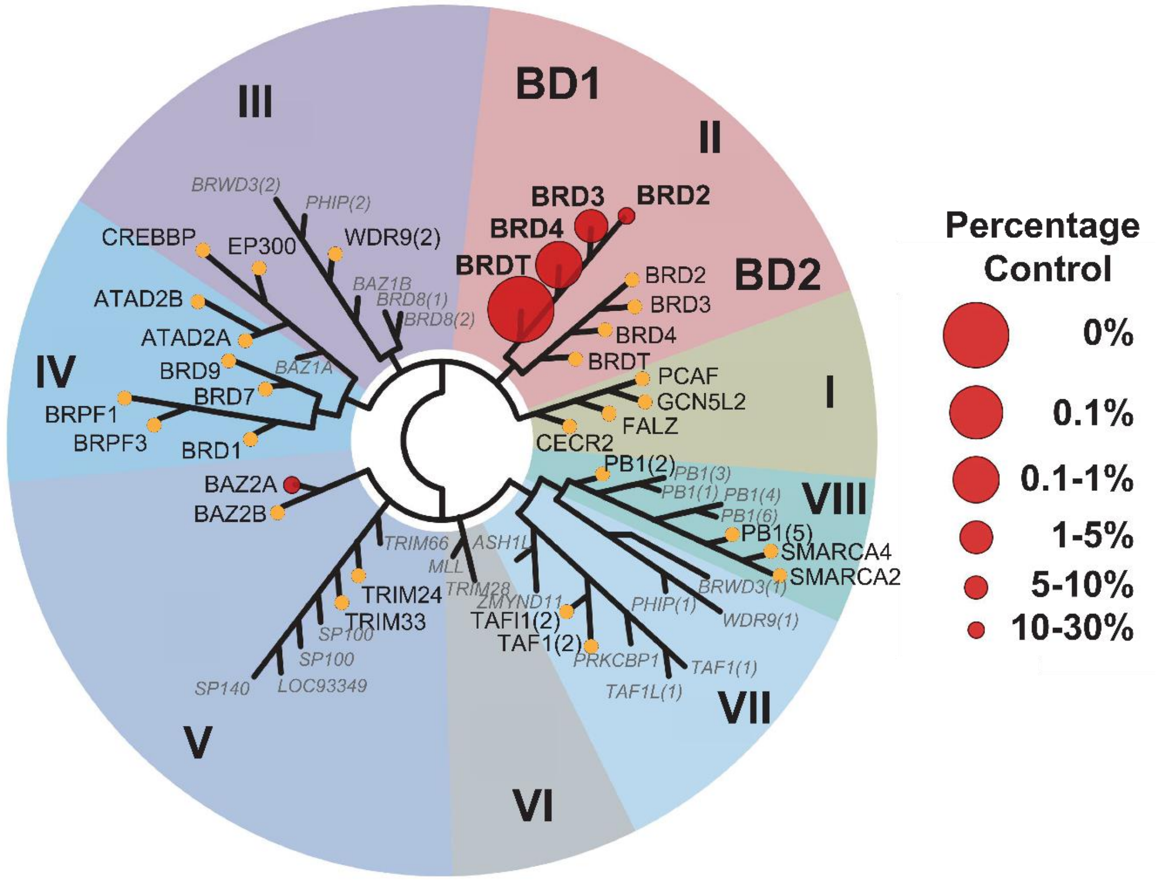

Figure S5. Detailed TREEspot phylogenetic tree showing binding of 1 µM 3.1B to 32 diverse human BDs in a BROMOscan competition binding assay. Larger spot size reflects stronger affinity. BDs that were tested but showed no interaction are shown as orange circles and labelled. BDs that were not tested are indicated by nodes labelled in *grey italics*. The BET family are family II.

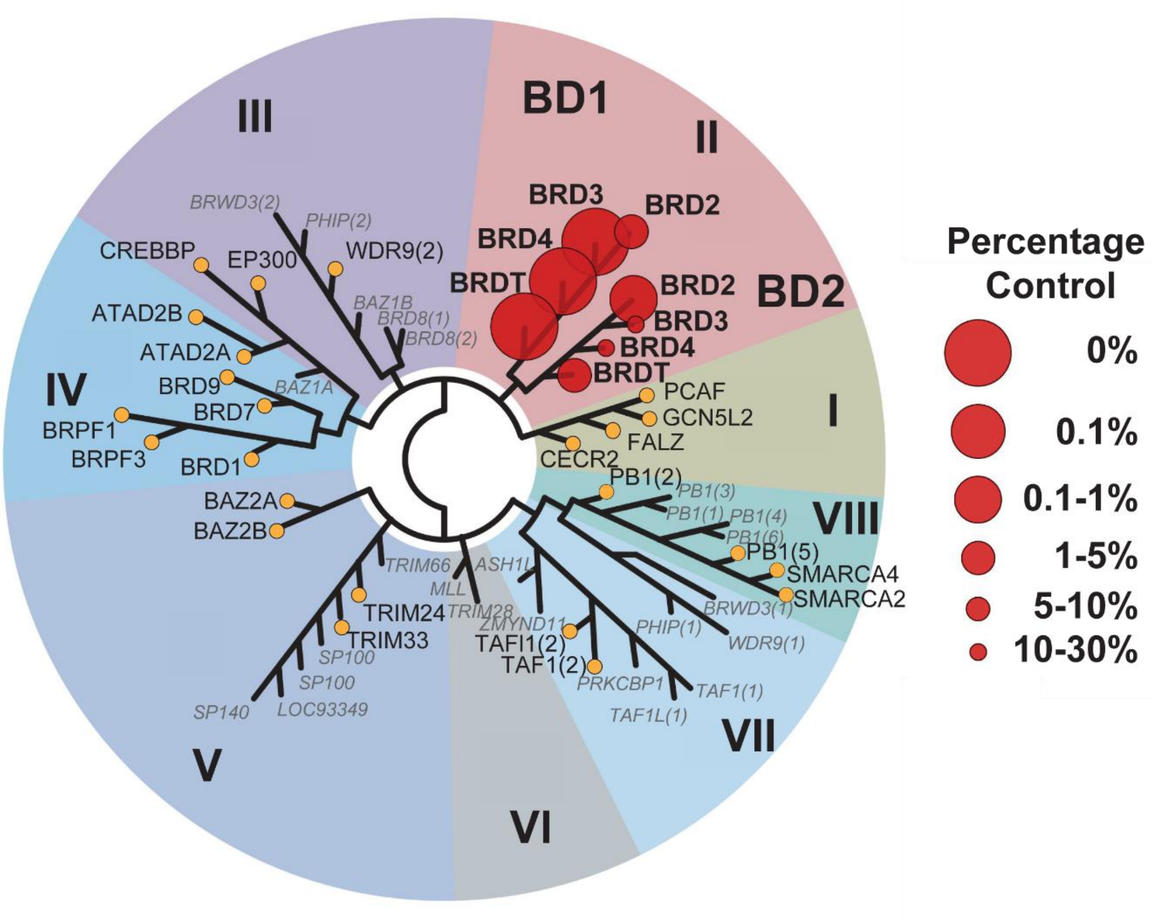

Figure S6. Detailed TREEspot phylogenetic tree showing binding of 1 µM 3.1C to 32 diverse human BDs in a BROMOscan competition binding assay. Larger spot size reflects stronger affinity. BDs that were tested but showed no interaction are shown as orange circles and labelled. BDs that were not tested are indicated by nodes labelled in *grey italics*. The BET family are family II

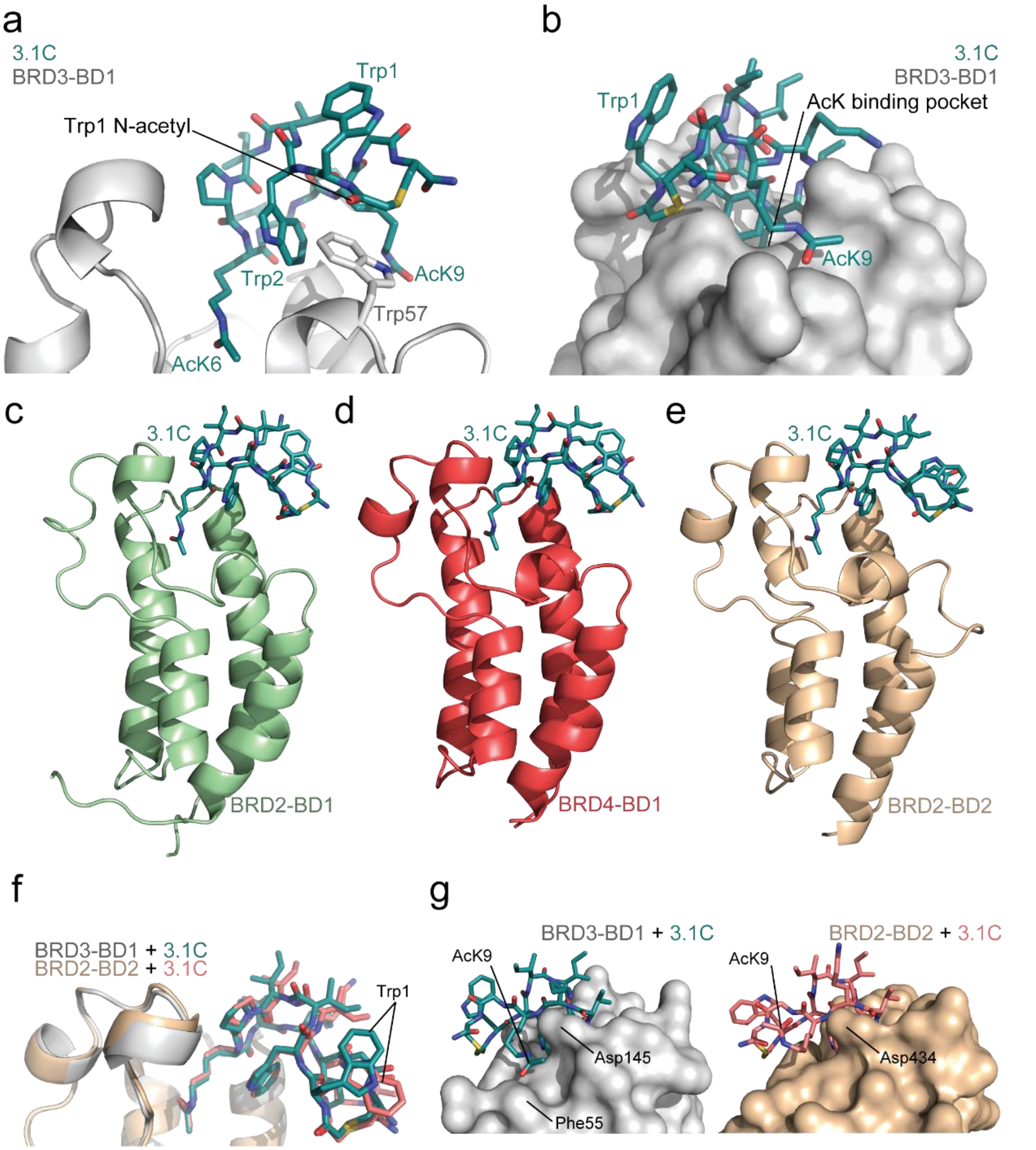

Figure S7. X-ray crystal structures of BRD2-BD1, BRD4-BD1 and BRD2-BD2 bound to 3.1C. *(A)* The structure of BRD3-BD1 (*grey*) in complex with **3.1C** (*teal*) showing the sandwich of Trp57 from the BD between the Trp2 sidechain and the N-acetyl moiety of Trp1 from **3.1C**. *(B)* Surface representation of BRD3-BD1 (*grey*) when in complex with **3.1C** (*teal*). AcK9 of **3.1C** interacts with a groove formed on the surface of the BD between the αZ and αD helices at a location distal to the AcK binding pocket. *(C)* Ribbon representation of the X-ray crystal structure of BRD2-BD1 (*pale green*) bound to **3.1C** (*teal,* 2.3-Å resolution, PDB ID 6U61). *(D)* Ribbon representation of the X-ray crystal structure of BRD4-BD1 (*coral red*) bound to **3.1C** (*teal,* 1.7-Å resolution, PDB IDs 6U6K). *(E)* Ribbon representation of the X-ray crystal structure of BRD2-BD2 (*wheat*) bound to **3.1C** (*teal,* 1.5-Å resolution, PDB ID 6U71). *(F)* Overlay of the BRD3-BD1:**3.1C** complex with the BRD2-BD2:**3.1C** complex. One of the major differences between the two peptide structures, the 180° flipping of the Trp1 sidechain, is indicated. *(G)* Comparison of the orientation of AcK9 of **3.1C** and the surface representation the two BDs from the BRD3-BD1:**3.1C** and BRD2-BD2:**3.1C** complexes.

BRD2-BD2 lacks the groove that AcK9 rests upon in the BRD3-BD1:**3.1C** structure leading to the residue being oriented upwards into the solvent in the BRD2-BD2:**3.1C** structure.

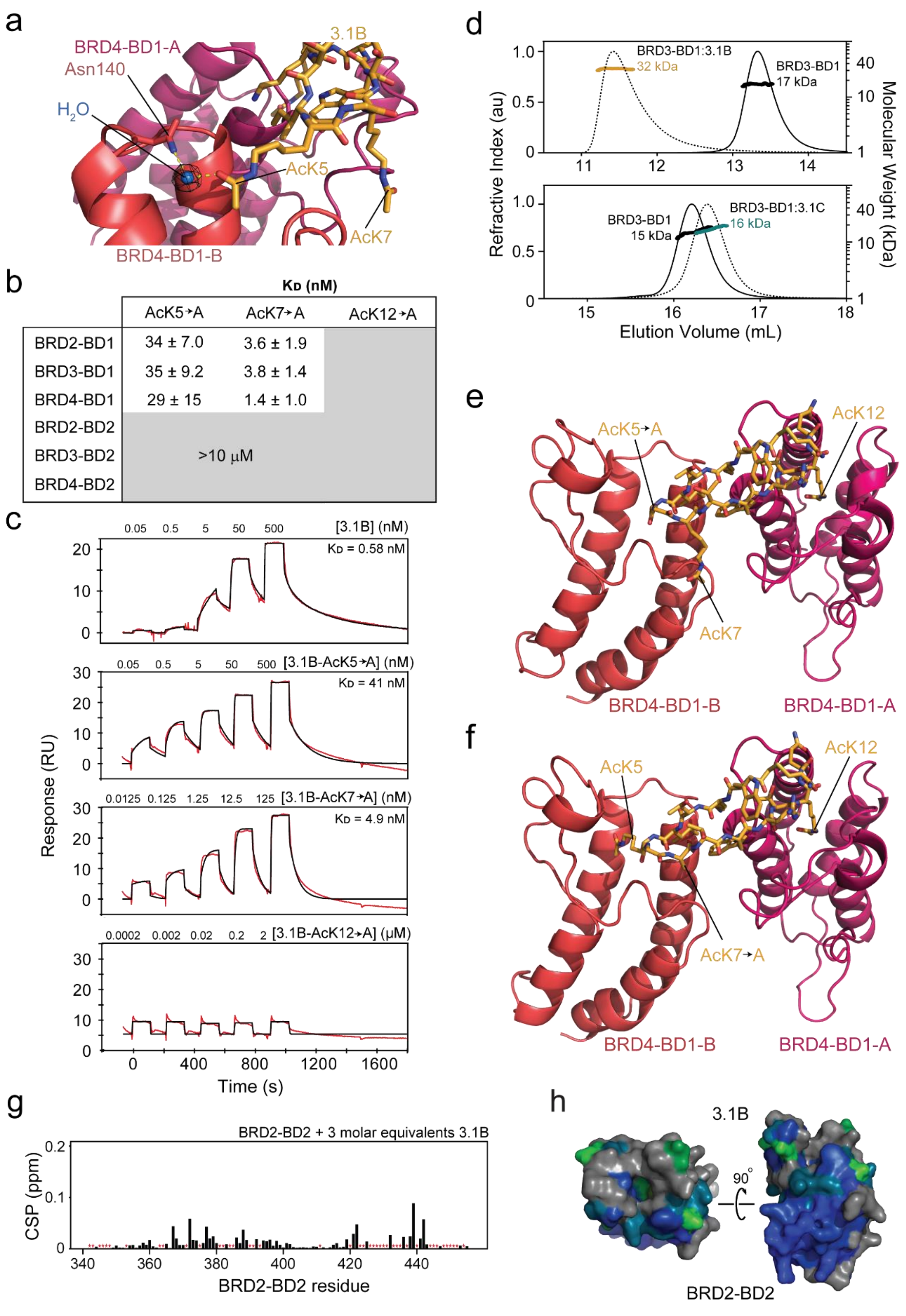
a
BRD4-BD1-A
Asn140
3.1B
H2O
AcK5
AcK7
BRD4-BD1-B
b
KD (nM)
AcK5→A
AcK7→A
AcK12→A
BRD2-BD1 34 ± 7.0 3.6 ± 1.9
BRD3-BD1 35 ± 9.2 3.8 ± 1.4
BRD4-BD1 29 ± 15 1.4 ± 1.0
BRD2-BD2
BRD3-BD2
BRD4-BD2
>10 µM
c
0.05 0.5 5 50 500 [3.1B] (nM)
KD = 0.58 nM
0.05 0.5 5 50 500 [3.1B-AcK5→A] (nM)
KD = 41 nM
0.0125 0.125 1.25 12.5 125 [3.1B-AcK7→A] (nM)
KD = 4.9 nM
0.0002 0.002 0.02 0.2 2 [3.1B-AcK12→A] (µM)
Response (RU)
Time (s)
d
BRD3-BD1:3.1B
32 kDa
BRD3-BD1
17 kDa
BRD3-BD1
15 kDa
BRD3-BD1:3.1C
16 kDa
Refractive Index (au)
Molecular Weight (kDa)
Elution Volume (mL)
e
AcK5→A
AcK12
AcK7
BRD4-BD1-B
BRD4-BD1-A
f
AcK5
AcK12
AcK7→A
BRD4-BD1-B
BRD4-BD1-A
g
BRD2-BD2 + 3 molar equivalents 3.1B
CSP (ppm)
BRD2-BD2 residue
h
3.1B
90°
BRD2-BD2

Figure S8. Analysis of BRD3/4-BD1:3.1B complexes. *(A)* A close up view of the water-mediated interaction between Asn140 of BRD4-BD1-B (*coral red*) and AcK5 of **3.1B** (*orange*). AcK5 enters the binding pocket at a distinct angle to the canonical AcK binding mode and forms a hydrogen bond with a water molecule (shown as a *blue sphere*) which in turn forms a hydrogen bond with Asn140. The electron density for the water is shown and hydrogen bonding is indicated by yellow dashed lines. *(B)* Dissociation constants for the binding of each of the six BDs to **3.1B** AcK mutants. *(C)* Representative SPR data (*red*) for the binding of BRD3-BD1 to **3.1B** (*top*) and to AcK5→Ala, AcK7→Ala and AcK12→Ala mutants. Fits to a simple 1:1 binding model (*black*) are shown and derived K_Ds are indicated on each plot. *(D) Top:* Size-exclusion chromatogram for BRD3-BD1 alone (solid line) and in the presence of one molar equivalent of **3.1B** (dashed line). The calculated molecular weight trace from MALLS analysis is also shown. BRD3-BD1 alone elutes as a single peak with the expected mass of 17 kDa. Addition of one molar equivalent of **3.1B** (Mw =1.9 kDa) shifts the peak to an earlier elution time and yields a mass of 32 kDa, in close agreement with the expected mass of a 2:1 BD-**3.1B** complex. *Bottom:* Size-exclusion chromatogram for BRD3-BD1 alone (solid line) and in the presence of one molar equivalent of **3.1C** (dashed line). The calculated molecular weight trace from MALLS analysis is also shown. *(E)* Ribbon diagram of the BRD4-BD1:**3.1B**_AcK5→Ala X-ray crystal structure (2.3-Å resolution, PDB ID 6U72). The peptide is shown in *orange* and the two BDs bound to the peptide are shown in pink (BRD4-BD1-A) and *coral red* (BRD4-BD1-B). The AcK5→Ala replacement and remaining AcKs are indicated. *(F)* Ribbon diagram of the BRD4-BD1:**3.1B**_AcK7→Ala X-ray crystal structure (2.6-Å resolution, PDB ID 6U8G). The peptide is shown in *orange* and the two BDs bound to the peptide are shown in pink (BRD4-BD1-A) and *coral red* (BRD4-BD1-B). The AcK7→Ala replacement and remaining AcKs are indicated. *(G)* Chemical shift perturbations in the ^{15}N-HSQC of BRD2-BD2 upon formation of complexes following addition of three molar equivalents of **3.1B**. The red stars indicate unassigned residues. *(H)* CSPs observed upon formation of the **3.1B** complex mapped onto the structure of BRD2-BD2 (PDB: 3ONI)[9]. The degrees of CSP are displayed through a blue-green-red gradient: the blue portions of the structure do not undergo any CSPs and the red sections represent the residues that display the greatest CSPs. Grey regions indicate unassigned residues.

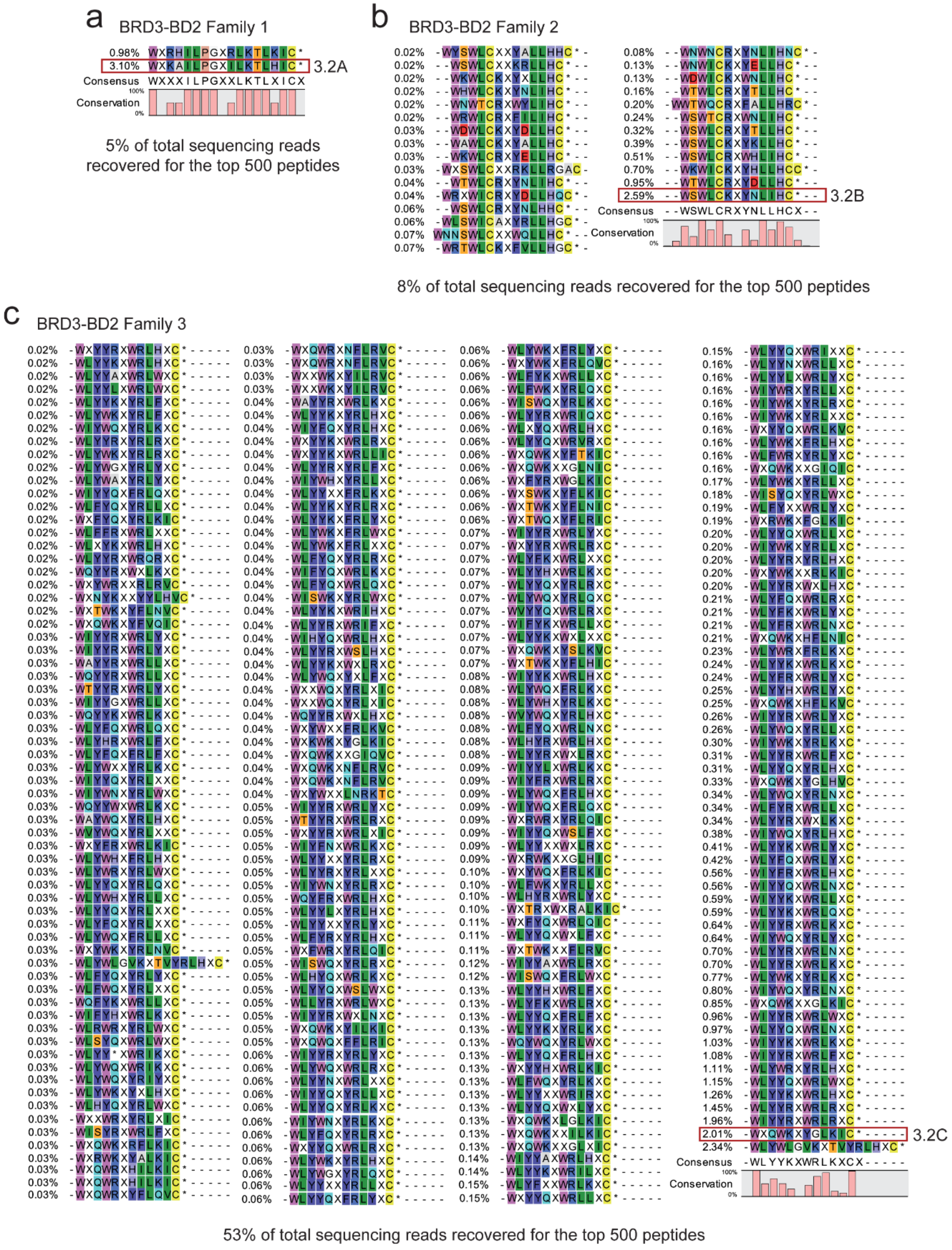

Figure S9. Sequences of all peptides from the BRD3-BD2 selection that are members of the families to which **3.2A** *(A)*, **3.2B** *(B)*, and **3.2C** *(C)* belong. The percentages next to each peptide are the proportions of the total sequencing reads that were recovered for the peptide. The

proportion of total sequencing reads recovered for the top 500 peptides is indicated below each family.

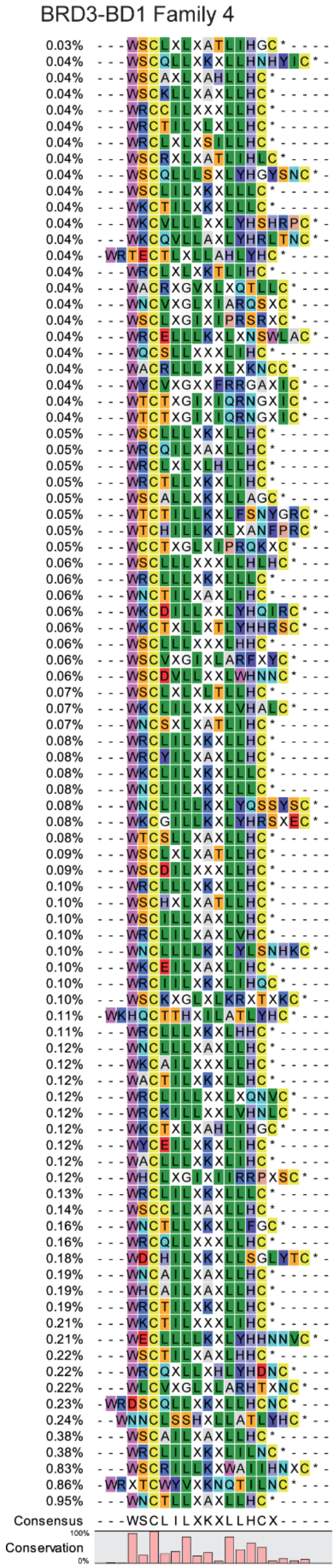

19% of total sequencing reads
recovered for the top 500 peptides

Figure S10. Sequences of all peptides belonging to BRD3-BD1 family 4. This family is related to BRD3-BD2 family 2 and BRD4-BD2 family 1.

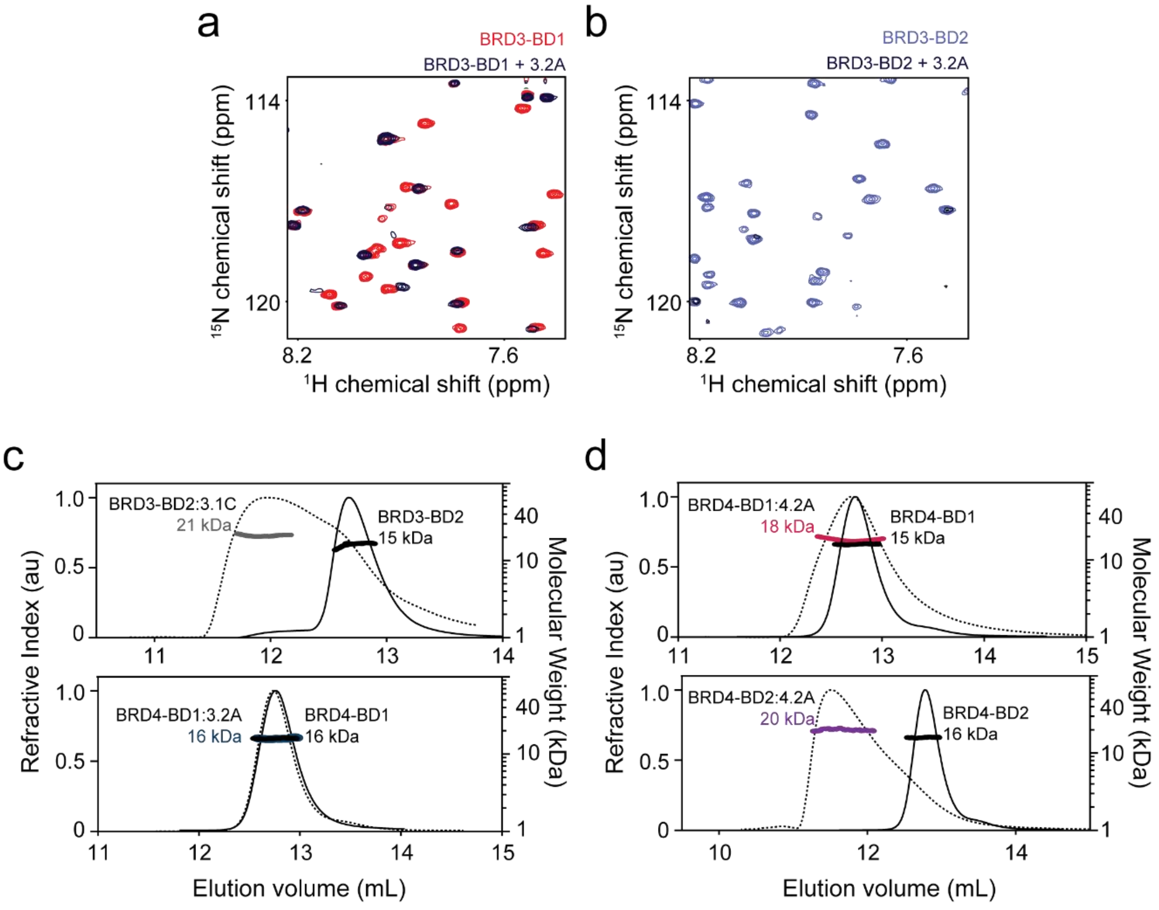

Figure S11. Analysis of 3.2A, 3.2C, and 4.1A interactions. *(A)* Overlaid ^{15}N-HSQC spectra of BRD3-BD1 alone (*red*) or in the presence of 1.5 molar equivalents of **3.2A** (*dark purple*). *(B)* Overlaid ^{15}N-HSQC spectra of BRD3-BD2 alone (*light purple*) or in the presence of 0.5 molar equivalents of **3.2A**. There is a mass disappearance of signals upon addition of **3.2A**. *(C) Top:* Size-exclusion chromatogram for BRD3-BD2 alone (solid line) and in the presence of 0.5 molar equivalents of **3.2C** (dashed line). The calculated molecular weight from MALLS analysis is also shown. Addition of 0.5 molar equivalents of **3.2C** leads to a shift from a peak that yields a mass of 15 kDa (BRD3-BD2 alone) to species with an earlier elution time that yields a molecular weight of 21 kDa, suggesting the formation of a weak 2:1 BD:**3.2C** complex. *Bottom:* Size-exclusion chromatogram for BRD4-BD1 alone (solid line) and in the presence of 0.5 molar equivalents of **3.2A** (dashed line). The calculated molecular weight from MALLS analysis is also shown. *(D) Top:* Size-exclusion chromatogram for BRD4-BD1 alone (solid line) and in the presence of 0.5 molar equivalent of **4.2A** (dashed line). The calculated molecular weight trace from MALLS analysis is also shown. *Bottom:* Size-exclusion chromatogram for BRD4-BD2 alone (solid line) and in the presence of one molar equivalent of **3.1C** (dashed line). The calculated molecular weight trace from MALLS analysis is also shown. BRD4-BD1 alone elutes as a single peak with the expected mass of 15 kDa. Addition of 0.5 molar equivalent of **4.2A** causes peak broadening and yields a mass of 18 kDa, consistent with a 1:1 BD:**4.2A** complex. Addition of 0.5 molar equivalents of **4.2A** to BRD4-BD2 leads to a shift from a peak that yields a mass of 16 kDa (BRD4-BD2 alone) to a species with an earlier elution time that yields a molecular weight of 20 kDa, indicating the formation of a weak 2:1 BD:**4.2A** complex.

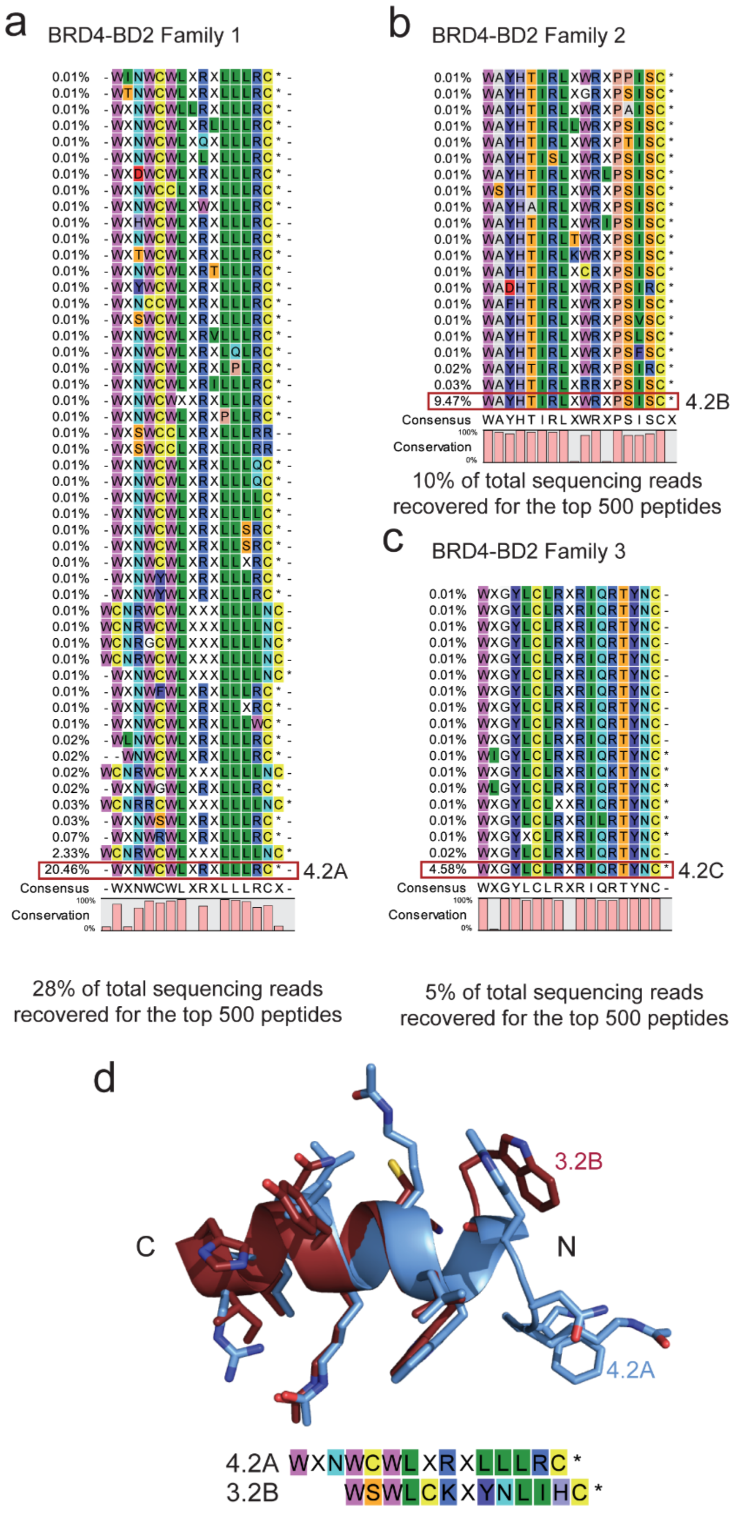

Figure S12. Sequences of all peptides from the BRD4-BD2 selection that are members of the families to which **4.2A** *(A)*, **4.2B** *(B)*, and **4.2C** *(C)* belong. The percentages next to each peptide are the proportions of the total sequencing reads that were recovered for the peptide. The proportion of total sequencing reads recovered for the top 500 peptides is indicated below each family. *(D)* Overlay of the structures and alignment of the sequences of peptides **3.2B** and **4.2C**.

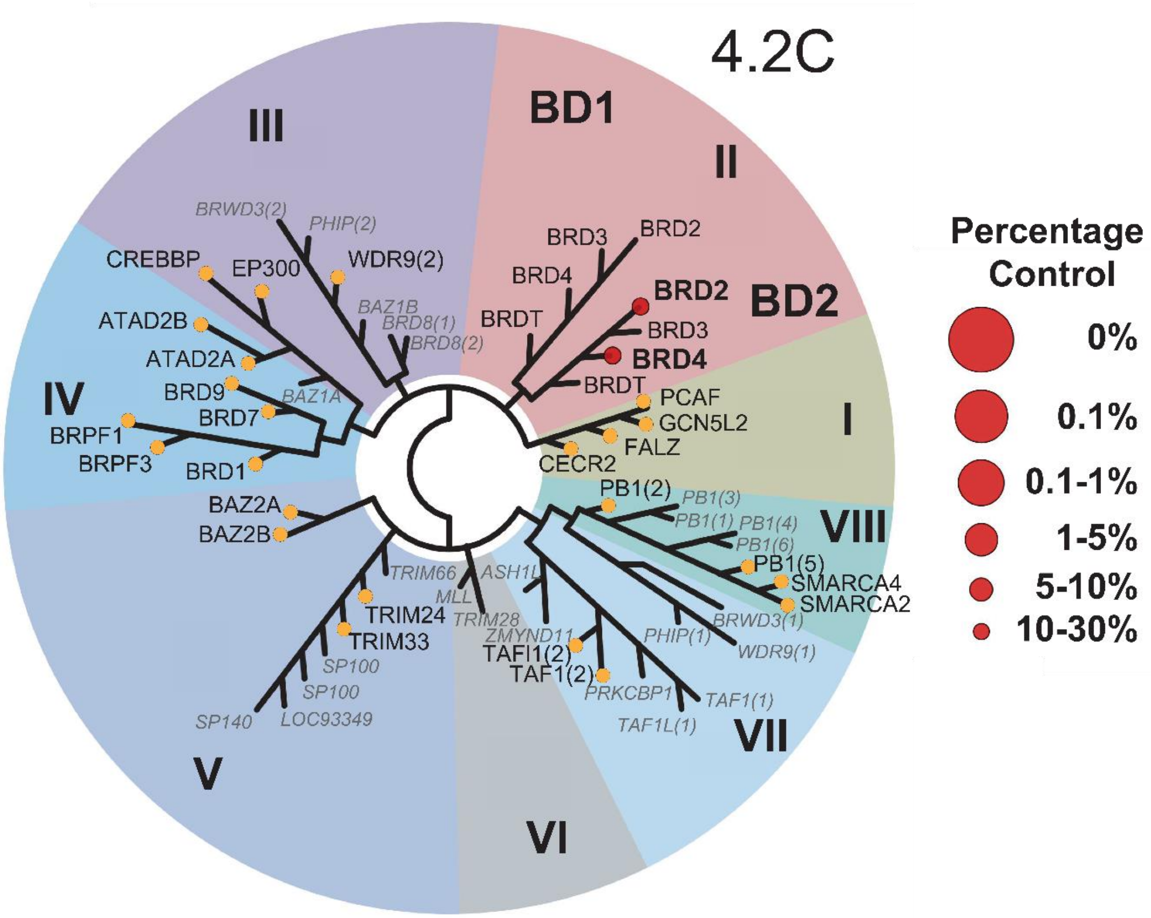

Figure S13. Detailed TREEspot phylogenetic tree showing binding of 1 µM 4.2C to 32 diverse human BDs in a BROMOscan competition binding assay. Larger spot size reflects stronger affinity. BDs that were tested but showed no interaction are shown as orange circles and labelled. BDs that were not tested are indicated by nodes labelled in *grey italics*. The BET family are family II

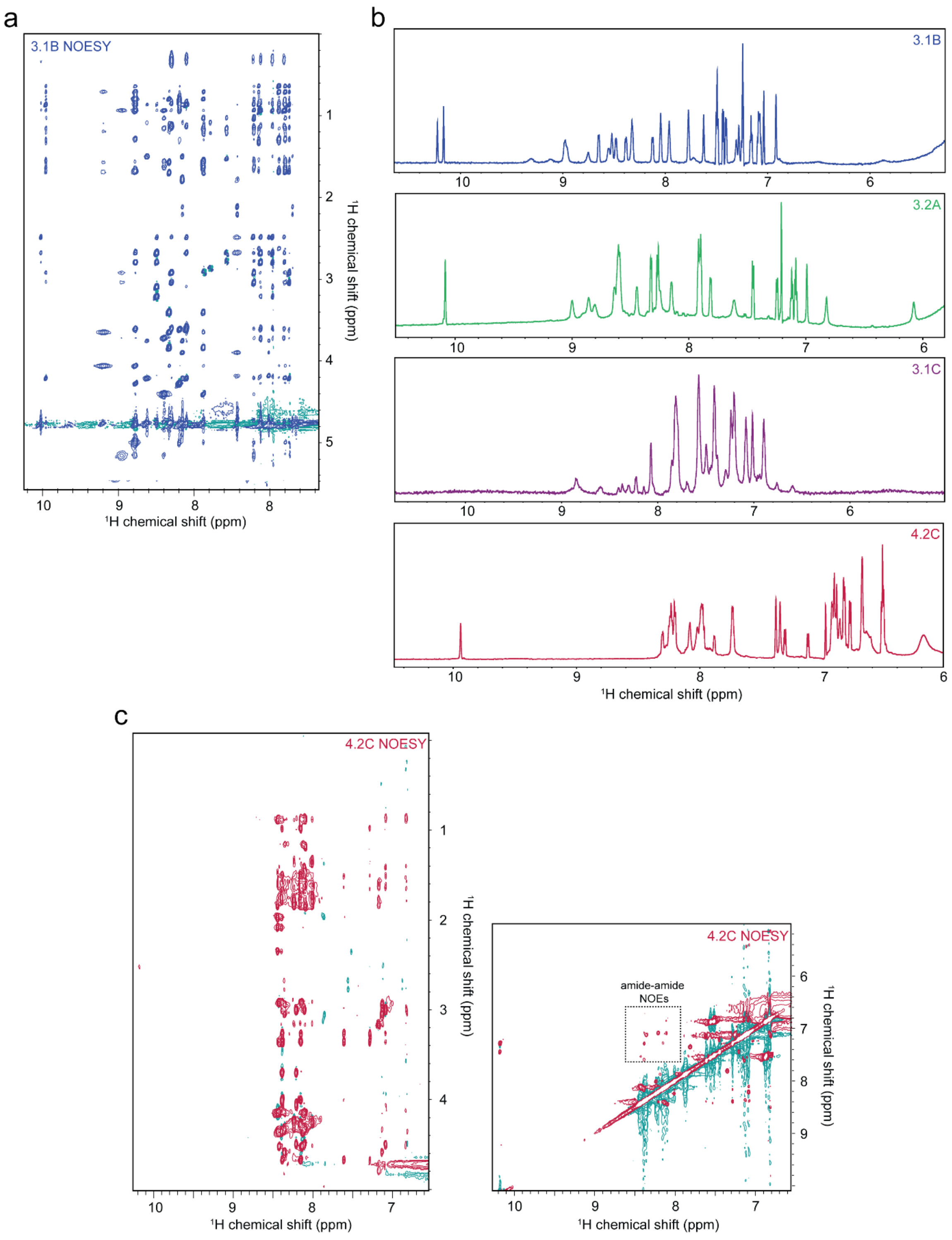

Figure S14. Two-dimensional NOESY and one-dimensional ^{1}H-NMR spectra of selected peptides. *(A)* Section of a two-dimensional NOESY spectra of **3.1B**. *(B)* One-dimensional ^{1}H-NMR spectra of **3.1B**, **3.2A**, **3.1C**, and **4.2C**. *(C)* Sections of two-dimensional NOESY spectra of **4.2C**. All spectra were collected at 25 ºC on an 800-MHz spectrometer.

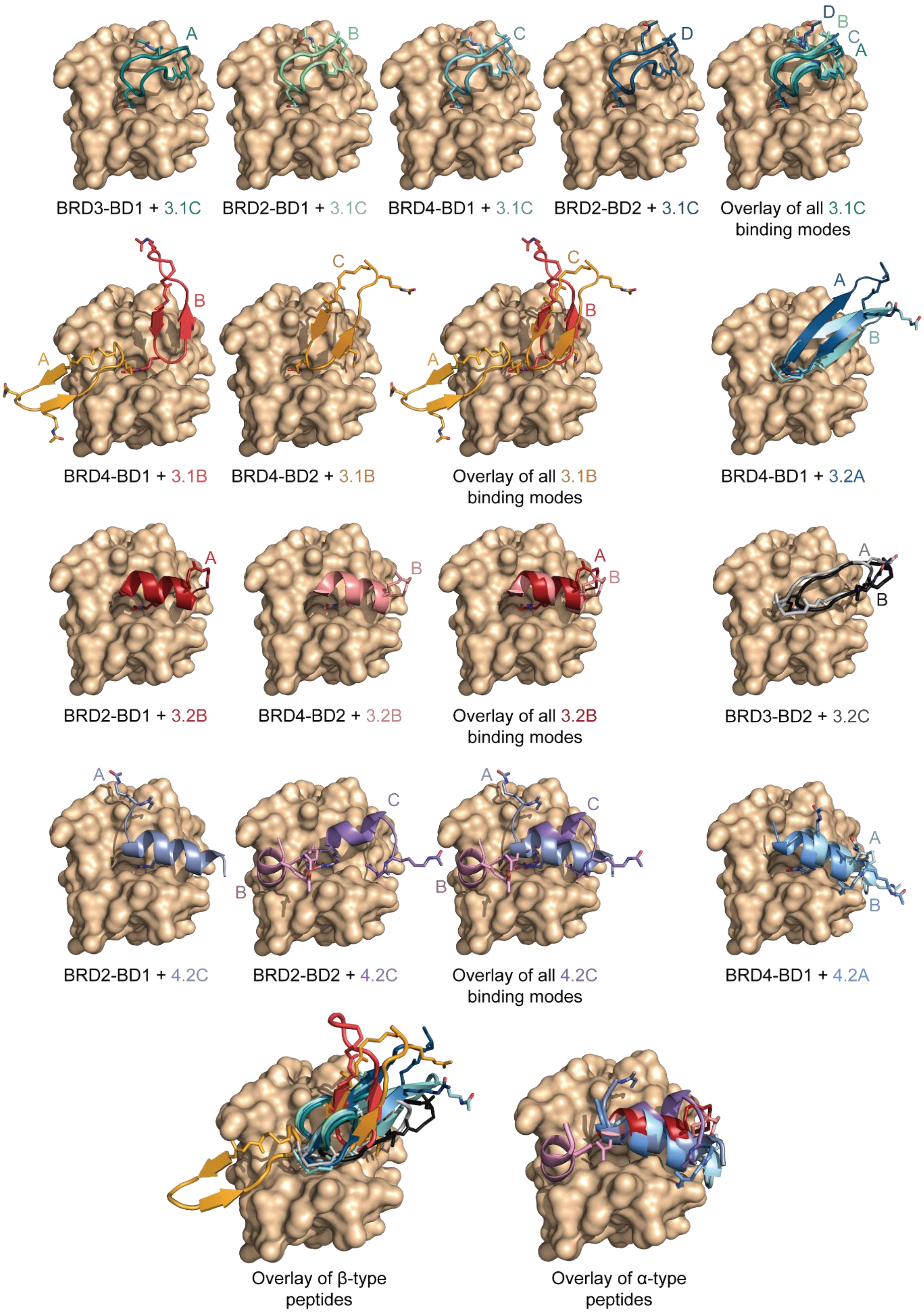

Figure S15. RaPID cyclic peptide structure summary

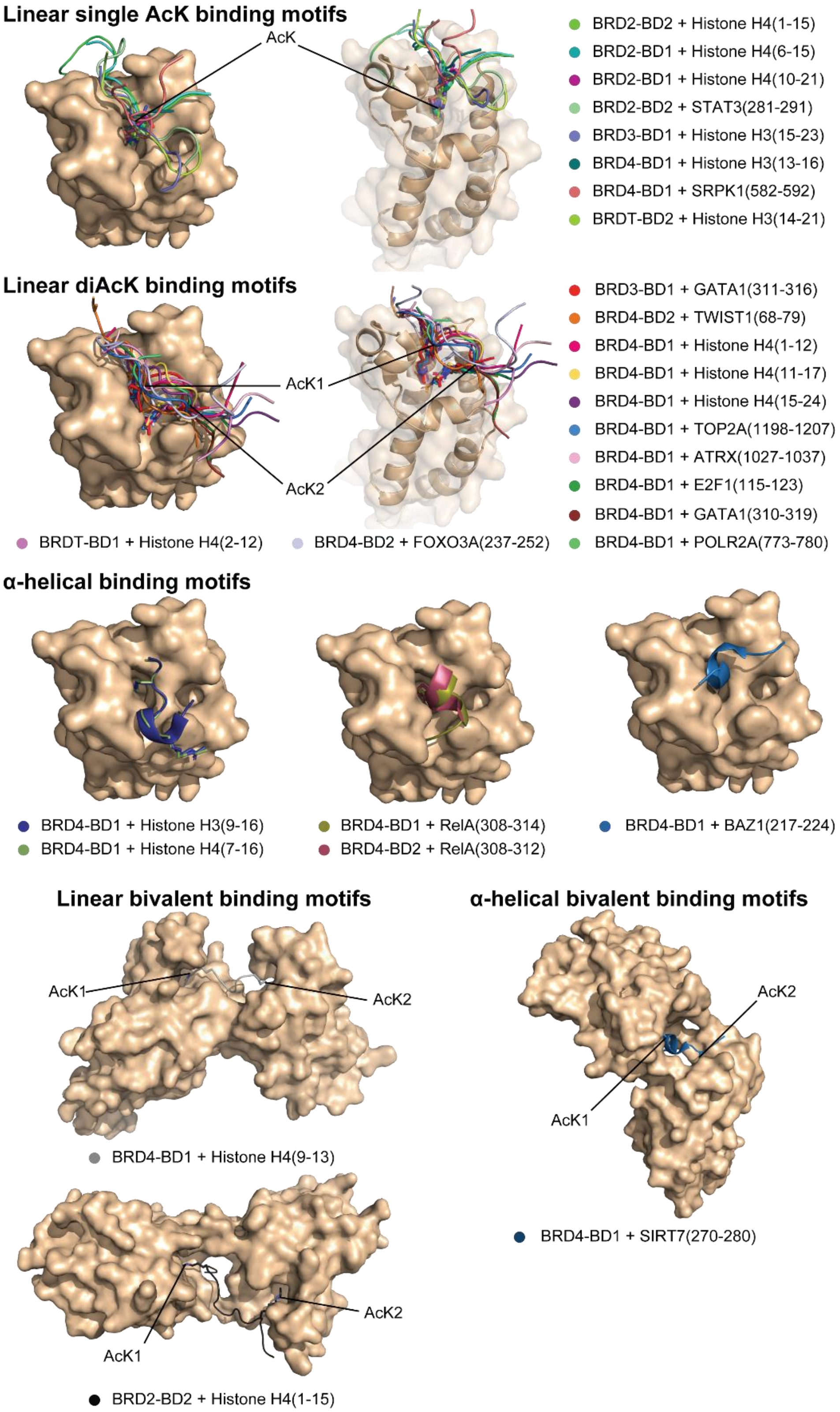

Figure S16. Summary of all structures reported of BET BDs in complex with native ligands. PDB codes for each structure displayed in the linear single AcK binding motifs section: **2DVQ**

BRD2-BD2 + Histone H4(1-15)[18], **2DVR** BRD2-BD1 + Histone H4(6-15)[18], **2DVS** BRD2-BD1 + Histone H4(10-21)[18], **5U5S** BRD2-BD2 + STAT3(281-291)[19], **5HJC** BRD3-BD1 + Histone H3(15-23)[20], **3JVK** BRD4-BD1 + Histone H4(13-16)[21], **5NNG** BRD4-BD1 + SRPK1(582-592)[22], and **2WP1** BRDT-BD2 + HistoneH3(14-21)[23]. PDB codes for each structure displayed in the linear diAcK binding motifs section: **2I5E** BRD3-BD1 + GATA1(311-316)[17], **2MJV** BRD4-BD2 + TWIST1(68-79)[24], **3UVW** BRD4-BD1 + Histone H4(1-12)[25], **3UVX** BRD4-BD1 + Histone H4(11-17)[25], **5NNE** BRD4-BD1 + TOP2A(1198-1037)[22], **6G0P** BRD4-BD1 + E2F1(115-123)[22], **6G0Q** BRD4-BD1 + GATA1(310-319)[22], **6G0R** BRD4-BD1 + POLR2A(773-780)[22], **6MNL** BRD4-BD2 + FOXO3A(237-252)[26], and **2WP2** BRDT-BD1 + Histone H4(2-12)[23]. PDB codes for structures displayed in the α-helical binding motifs section: **5NNC** BRD4-BD1 + Histone H3(9-16)[22], **5NND** BRD4-BD1 +Histone H4(7-16)[22], **4KV1** BRD4-BD1 + RelA(308-314)[27], **4KV4** BRD4-BD2 + RelA(308-312)[27], and **5NNF** BRD4-BD1 + BAZ1(217-224)[22]. PDB codes for structures displayed in the linear bivalent binding motifs section: **2E3K** BRD4-BD1 + Histone H3(9-13)[18] and **3UW9** BRD2-BD2 + Histone H4(1-15)[25]. PDB codes for structures displayed in the α-helical bivalent binding motifs section: **6G0S** BRD4-BD1 + SIRT7(270-280)[22].

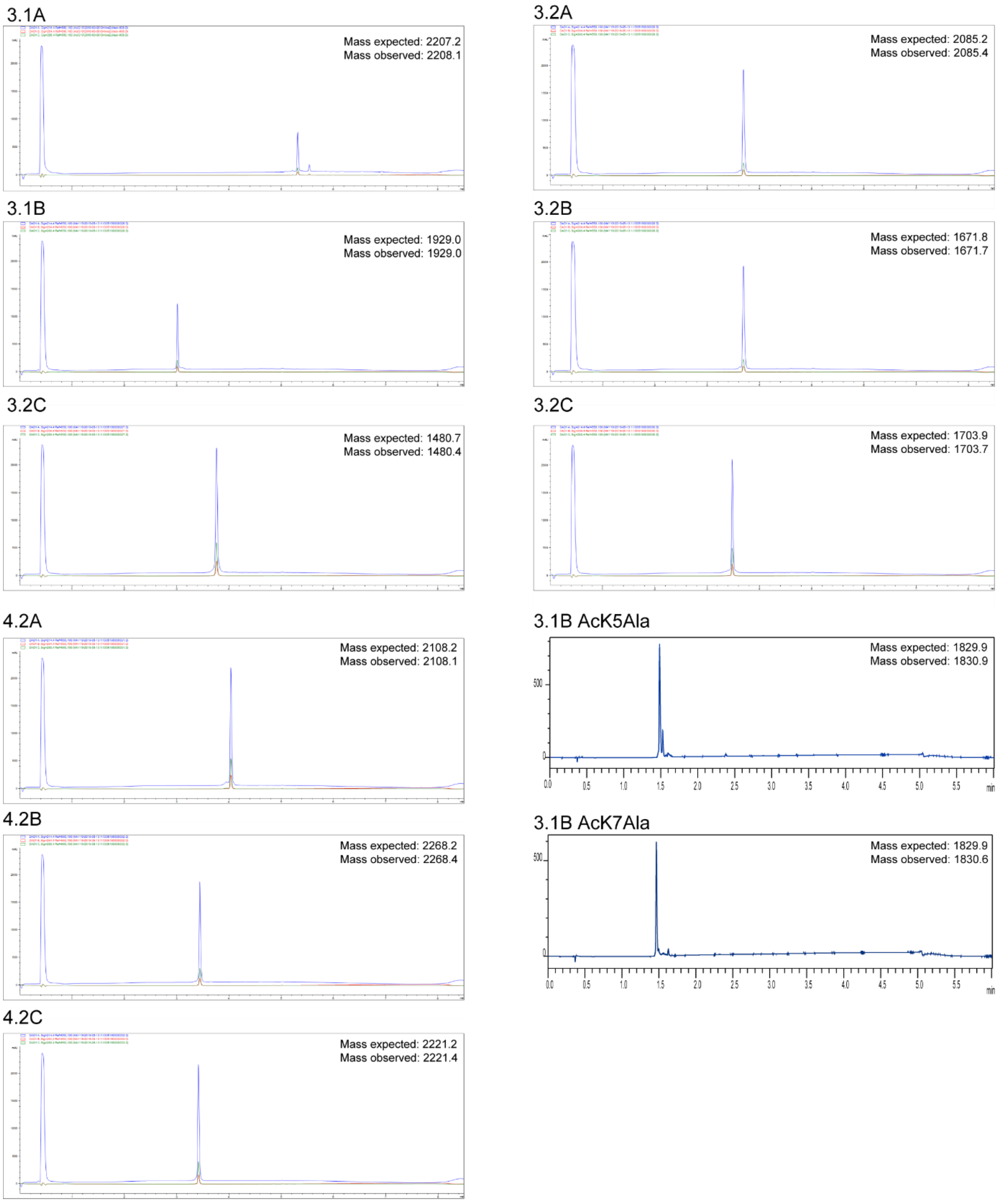

Figure S17. HPLC traces of all RaPID peptides

Table S1.
Data collection and refinement statistics for the crystal structures of the BRD3-BD1:**3.1C** (PDB ID 6U4A) and BRD2-BD1:**3.1C** (PDB ID 6U61) complexes solved by molecular replacement.

	BRD3-BD1:**3.1C**	BRD2-BD1:**3.1C**
**Data collection**		
Space group	P 21 21 21	C 2 2 21
Cell dimensions		
a, *b*, *c* (Å)	48.83, 64.42, 87.65	69.54, 86.75, 115.48
α, β, γ (°)	90.00, 90.00, 90.00	90.00, 90.00, 90.00
Resolution (Å)	1.88 (1.88-1.92)	2.29 (2.29-2.37)
R_{merge}	0.075 (0.515)	0.113 (0.775)
I / σI	16.4 (3.8)	13.9 (4.3)
Completeness (%)	99.9 (0.883)	99.6 (96.4)
Redundancy	7.4 (7.2)	13.2 (12.7)
**Refinement**		
Resolution (Å)	1.88	2.29
No. reflections	23024	16071
R_{work} / R_{free}	0.1637/0.1881	0.222/0.2634
No. atoms		
Protein	2268	2165
Ligand/ion	36	11
Water	206	77
B-factors	24.0	43.0
R.m.s. deviations		
Bond lengths (Å)	0.004	0.002
Bond angles (°)	0.674	0.519

*Values in parentheses are for highest-resolution shell.
All were data were collected from a single crystal.

Table S2.
Data collection and refinement statistics for the crystal structures of the BRD4-BD1:**3.1C** (PDB ID 6U6K) and BRD2-BD2:**3.1C** (PDB ID 6U71) complexes solved by molecular replacement.

	BRD4-BD1:**3.1C**	BRD2-BD2:**3.1C**
**Data collection**		
Space group	C 1 2 1	P 2 21 21
Cell dimensions		
a, *b*, *c* (Å)	121.96, 41.90, 29.54	31.82, 51.81, 71.79
α, β, γ (°)	90.00, 94.20, 90.00	90.00, 90.00, 90.00
Resolution (Å)	1.70 (1.70-1.73)	1.47 (1.47-1.50)
R_{merge}	0.061 (0.611)	0.062 (0.547)
I / σI	9.0 (1.8)	11.8 (2.7)
Completeness (%)	99.7 (99.7)	99.7 (99.3)
Redundancy	3.3 (3.2)	4.4 (4.4)
**Refinement**		
Resolution (Å)	1.70	1.47
No. reflections	16475	20844
R_{work} / R_{free}	0.1799/0.2177	0.1600/0.1991
No. atoms		
Protein	1152	1150
Ligand/ion	4	4
Water	110	116
B-factors	27.0	20
R.m.s. deviations		
Bond lengths (Å)	0.007	0.003
Bond angles (°)	0.934	0.615

*Values in parentheses are for highest-resolution shell.
All were data were collected from a single crystal.

Table S3.
Data collection and refinement statistics for the crystal structures of the BRD4-BD1:**3.1B** (PDB ID 6U74) and BRD4-BD1:**3.1B**_AcK5→Ala (PDB ID 6U72) complexes solved by molecular replacement.

	BRD4-BD1:**3.1B**	BRD4-BD1:**3.1B**_AcK5→Ala
**Data collection**		
Space group	P 1 21 1	P 1 21 1
Cell dimensions		
a, b, c (Å)	58.68, 49.23, 115.94	58.21, 49.05, 59.15
α, β, γ (°)	90.00, 102.58, 90.00	90.00, 103.87, 90.00
Resolution (Å)	1.85 (1.85-1.89)	2.30 (2.30-2.38)
R_{merge}	0.317 (2.03)	0.145 (0.638)
$I / \sigma I$	4.5 (1.0)	7.8 (2.7)
Completeness (%)	100.0 (100.0)	99.9 (99.9)
Redundancy	7.4 (7.4)	6.7 (7.0)
**Refinement**		
Resolution (Å)	1.85	2.30
No. reflections	54657	14555
R_{work} / R_{free}	0.2841/0.3234	0.2093/0.2661
No. atoms		
Protein	4161	2074
Ligand/ion	8	4
Water	457	63
B-factors	27.0	59.0
R.m.s. deviations		
Bond lengths (Å)	0.002	0.003
Bond angles (°)	0.570	0.533

*Values in parentheses are for highest-resolution shell.
All were data were collected from a single crystal.

Table S4.
Data collection and refinement statistics for the crystal structures of the BRD4-BD1:**3.1B**_AcK7→Ala (PDB ID 6U8G) and BRD4-BD2:**3.1B** (PDB ID 6U6L) complexes solved by molecular replacement.

	BRD4-BD1:**3.1B**_AcK7→Ala	BRD4-BD2:**3.1B**
**Data collection**		
Space group	P 1 21 1	P 2 21 21
Cell dimensions		
a, *b*, *c* (Å)	58.71, 48.98, 114.15	32.40, 56.51, 73.34
α, β, γ (°)	90.00, 101.66, 90.00	90.00, 90.00, 90.00
Resolution (Å)	2.6 (2.6-2.72)	2.6 (2.6-2.72)
R_{merge}	0.237 (1.021)	0.103 (0.636)
$I / \sigma I$	6.1 (2.6)	12.6 (2.8)
Completeness (%)	99.8 (100.0)	97.3 (98.6)
Redundancy	5.7 (5.6)	4.7 (4.7)
**Refinement**		
Resolution (Å)	2.60	2.60
No. reflections	19831	4472
R_{work} / R_{free}	0.2416/0.2965	0.2160/0.2425
No. atoms		
Protein	4169	1043
Ligand/ion	8	10
Water	45	16
B-factors	47.0	42.0
R.m.s. deviations		
Bond lengths (Å)	0.001	0.002
Bond angles (°)	0.462	0.525

*Values in parentheses are for highest-resolution shell.
All were data were collected from a single crystal.

Table S5.
Data collection and refinement statistics for the crystal structures of the BRD3-BD2:**3.2C** (PDB ID 6ULP) and BRD4-BD1:**3.2A** (PDB ID 6U8M) complexes solved by molecular replacement.

	BRD3-BD2:**3.2C**	BRD4-BD1:**3.2A**
**Data collection**		
Space group	I 4	P 21 21 21
Cell dimensions		
a, *b*, *c* (Å)	97.57, 97.57, 77.75	61.83, 72.62, 86.06
α, β, γ (°)	90.00, 90.00, 90.00	90.00, 90.00, 90.00
Resolution (Å)	2.80 (2.80-2.96)	1.95 (1.95-2.00)
R_{merge}	0.049 (0.832)	0.061 (0.432)
$I / \sigma I$	29.9 (3.4)	22.1 (4.8)
Completeness (%)	99.8 (98.8)	100.0 (100.0)
Redundancy	14.0 (14.2)	13.0 (13.2)
**Refinement**		
Resolution (Å)	2.80	1.95
No. reflections	9017	28898
R_{work} / R_{free}	0.2102/0.2477	0.1700/0.1999
No. atoms		
Protein	1932	2117
Ligand/ion	4	4
Water	0	214
B-factors	110.0	41.0
R.m.s. deviations		
Bond lengths (Å)	0.002	0.008
Bond angles (°)	0.559	0.912

*Values in parentheses are for highest-resolution shell.
All were data were collected from a single crystal.

Table S6.
Data collection and refinement statistics for the crystal structures of the BRD2-BD1:**3.2B** (PDB ID 6U8H) and BRD4-BD2:**3.2B** (PDB ID 6U8I) complexes solved by molecular replacement.

	BRD2-BD1:**3.2B**	BRD4-BD2:**3.1B**
**Data collection**		
Space group	P 41 21 2	P 4
Cell dimensions		
a, *b*, *c* (Å)	89.59, 89.59, 71.59	69.78, 69.78, 32.15
α, β, γ (°)	90.00, 90.00, 90.00	90.00, 90.00, 90.00
Resolution (Å)	2.07 (2.07-2.13)	2.50 (2.50-2.70)
R_{merge}	0.062 (0.322)	0.165 (0.593)
$I / \sigma I$	25.0 (7.9)	6.4 (2.5)
Completeness (%)	99.7 (97.0)	100.00 (100.0)
Redundancy	12.2 (12.0)	4.9 (4.9)
**Refinement**		
Resolution (Å)	2.07	2.50
No. reflections	18148	5552
R_{work} / R_{free}	0.1819/0.2232	0.1892/0.2395
No. atoms		
Protein	1169	1004
Ligand/ion	65	4
Water	129	59
B-factors	34.0	28.0
R.m.s. deviations		
Bond lengths (Å)	0.002	0.002
Bond angles (°)	0.465	0.439

*Values in parentheses are for highest-resolution shell.
All were data were collected from a single crystal.

Table S7.
Data collection and refinement statistics for the crystal structures of the BRD4-BD1:**4.2A** (PDB ID 6ULV) complex solved by molecular replacement.

	BRD4-BD1:**4.2A**
**Data collection**	
Space group	P 65 2 2
Cell dimensions	
a, *b*, *c* (Å)	112.29, 112.29, 235.58
α, β, γ (°)	90.00, 90.00, 120.00
Resolution (Å)	2.20 (2.20-2.27)
R_{merge}	0.474 (7.386)
$I / \sigma I$	9.2 (1.4)
Completeness (%)	100.0 (100.0)
Redundancy	33.3 (34.0)
**Refinement**	
Resolution (Å)	2.20
No. reflections	45317
R_{work} / R_{free}	0.1984 (0.2377)
No. atoms	
Protein	3957
Ligand/ion	46
Water	240
B-factors	45.0
R.m.s. deviations	
Bond lengths (Å)	0.004
Bond angles (°)	0.682

*Values in parentheses are for highest-resolution shell.
All were data were collected from a single crystal.

Table S8.
Data collection and refinement statistics for the crystal structures of the BRD2-BD1:**4.2C** (PDB ID 6ULQ) and BRD2-BD2:**4.2C** (PDB ID 6ULT) complexes solved by molecular replacement.

	BRD2-BD1:**4.2C**	BRD2-BD2:**4.2C**
**Data collection**		
Space group	I 1 2 1	P 1 21 1
Cell dimensions		
a, *b*, *c* (Å)	80.48, 76.14, 109.66	77.08, 63.63, 120.34
α, β, γ (°)	90.00, 109.28, 90.00	90.00, 108.39, 90.00
Resolution (Å)	2.70 (2.70-2.83)	2.80 (2.80-2.95)
R_{merge}	0.277 (1.454)	0.165 (0.686)
$I / \sigma I$	9.5 (2.9)	6.4 (1.8)
Completeness (%)	99.6 (97.6)	98.8 (94.2)
Redundancy	6.7 (6.9)	3.7 (3.8)
**Refinement**		
Resolution (Å)	2.70	2.80
No. reflections	17421	27196
R_{work} / R_{free}	0.2215/0.2516	0.2814/0.3379
No. atoms		
Protein	3550	7462
Ligand/ion	9	12
Water	36	81
B-factors	34.0	55.0
R.m.s. deviations		
Bond lengths (Å)	0.002	0.003
Bond angles (°)	0.571	0.690

*Values in parentheses are for highest-resolution shell.
All were data were collected from a single crystal.

Table S9.
NMR and refinement statistics for the solution structure of cyclic peptide **3.1B** (PDB ID: 6UXS).

	Protein
**NMR distance and dihedral constraints**	
Distance constraints	
Total NOE	261
Intra-residue	91
Inter-residue	170
Sequential ($\|i - j\| = 1$)	66
Medium-range ($\|i - j\| < 4$)	28
Long-range ($\|i - j\| > 5$)	76
Intermolecular	0
Hydrogen bonds	0
Total dihedral angle restraints	
ϕ	0
ψ	0
**Structure statistics**	
Violations (mean and s.d.)	
Distance constraints (Å)	0
Dihedral angle constraints (º)	N/A
Max. dihedral angle violation (º)	N/A
Max. distance constraint violation (Å)	0
Deviations from idealized geometry	
Bond lengths (Å)	0.001
Bond angles (º)	0.2
Impropers (º)	N/A
Average pairwise r.m.s. deviation** (Å)	
Heavy	0.75
Backbone	0.02

*Pairwise r.m.s. deviation was calculated among 20 refined structures.

SI References

1. Murakami, H., Ohta, A., Ashigai, H. & Suga, H. A highly flexible tRNA acylation method for non-natural polypeptide synthesis. *Nat. Methods* **3**, 357–359 (2006).
2. Goto, Y., Goto, Y., Katoh, T. & Suga, H. Preparation of materials for flexizyme reactions and genetic code reprogramming. *Protoc. Exch.* (2011). doi:10.1038/protex.2011.209
3. Kawamura, A. *et al.* Highly selective inhibition of histone demethylases by de novo macrocyclic peptides. *Nat. Commun.* **8**, 14773 (2017).
4. Cowieson, N. P. *et al.* MX1: A bending-magnet crystallography beamline serving both chemical and macromolecular crystallography communities at the Australian Synchrotron. *J. Synchrotron Radiat.* **22**, 187–190 (2015).
5. Aragão, D. *et al.* MX2: a high-flux undulator microfocus beamline serving both the chemical and macromolecular crystallography communities at the Australian Synchrotron. *J. Synchrotron Radiat.* **25**, 885–891 (2018).
6. Winn, M. D. *et al.* Overview of the {\it CCP}4 suite and current developments. *Acta Crystallogr. Sect. D* **67**, 235–242 (2011).
7. Potterton, E., Briggs, P., Turkenburg, M. & Dodson, E. A graphical user interface to the {\it CCP}4 program suite. *Acta Crystallogr. Sect. D* **59**, 1131–1137 (2003).
8. Adams, P. D. *et al.* PHENIX: a comprehensive Python-based system for macromolecular structure solution. *Acta Crystallogr. Sect. D* **66**, 213–221 (2010).
9. Filippakopoulos, P. *et al.* Selective inhibition of BET bromodomains. *Nature* **468**, 1067–1073 (2010).
10. Lucas, X. *et al.* 4-Acyl pyrroles: Mimicking acetylated lysines in histone code reading. *Angew. Chemie - Int. Ed.* **52**, 14055–14059 (2013).
11. Morimoto, J., Hayashi, Y. & Suga, H. Discovery of macrocyclic peptides armed with a mechanism-based warhead: Isoform-selective inhibition of human deacetylase SIRT2. *Angew. Chemie - Int. Ed.* **51**, 3423–3427 (2012).
12. Gosmini, R. *et al.* The discovery of I-BET726 (GSK1324726A), a potent tetrahydroquinoline ApoA1 up-regulator and selective BET bromodomain inhibitor. *J. Med. Chem.* **57**, 8111–8131 (2014).
13. Emsley, P., Lohkamp, B., Scott, W. G. & Cowtan, K. Features and development of {\it Coot}. *Acta Crystallogr. Sect. D* **66**, 486–501 (2010).
14. Lee, W., Tonelli, M. & Markley, J. L. NMRFAM-SPARKY: enhanced software for biomolecular NMR spectroscopy. *Bioinformatics* **31**, 1325–1327 (2014).
15. Güntert, P. & Buchner, L. Combined automated NOE assignment and structure calculation with CYANA. *J. Biomol. NMR* **62**, 453–71 (2015).
16. Vranken, W. F. *et al.* The CCPN data model for NMR spectroscopy: Development of a software pipeline. *Proteins Struct. Funct. Genet.* **59**, 687–696 (2005).
17. Gamsjaeger, R. *et al.* Structural Basis and Specificity of Acetylated Transcription Factor GATA1 Recognition by BET Family Bromodomain Protein Brd3. *Mol. Cell. Biol.* **31**, 2632–2640 (2011).
18. Umehara, T. *et al.* Structural basis for acetylated histone H4 recognition by the human BRD2 bromodomain. *J. Biol. Chem.* **285**, 7610–7618 (2010).
19. Cheung, K. L. *et al.* Distinct Roles of Brd2 and Brd4 in Potentiating the Transcriptional Program for Th17 Cell Differentiation. *Mol. Cell* 1–13 (2017). doi:10.1016/j.molcel.2016.12.022
20. Li, Y. *et al.* Molecular Coupling of Histone Crotonylation and Active Transcription by AF9 YEATS Domain. *Mol. Cell* **62**, 181–193 (2016).
21. Vollmuth, F., Blankenfeldt, W. & Geyer, M. Structures of the dual bromodomains of the P-TEFb-activating protein Brd4 at atomic resolution. *J. Biol. Chem.* **284**, 36547–36556 (2009).
22. Lambert, J.-P. *et al.* Interactome Rewiring Following Pharmacological Targeting of BET Bromodomains. *Mol. Cell* **73**, 621–638 (2019).
23. Morinière, J. *et al.* Cooperative binding of two acetylation marks on a histone tail by a single bromodomain. *Nature* **461**, 664–668 (2009).
24. Shi, J. *et al.* Disrupting the Interaction of BRD4 with Diacetylated Twist Suppresses Tumorigenesis in Basal-like Breast Cancer. *Cancer Cell* **25**, 210–225 (2014).

25. Filippakopoulos, P. *et al.* Histone recognition and large-scale structural analysis of the human bromodomain family. *Cell* **149**, 214–231 (2012).
26. Liu, J. *et al.* Targeting the BRD4/FOXO3a/CDK6 axis sensitizes AKT inhibition in luminal breast cancer. *Nat. Commun.* **9**, 1–17 (2018).
27. Zou, Z. *et al.* Brd4 maintains constitutively active NF-κB in cancer cells by binding to acetylated RelA. *Oncogene* **33**, 2395–2404 (2014).
