# Supplementary material for: Cyclic peptides can engage a single binding pocket through highly divergent modes
Source: Proc Natl Acad Sci U S A. 2020 Oct 12;117(43):26728–38. doi: 10.1073/pnas.2003086117 (PMC7604503; doi:10.1073/pnas.2003086117)
Supplement: Supplementary File [file pnas.2003086117.sd02.pdf]

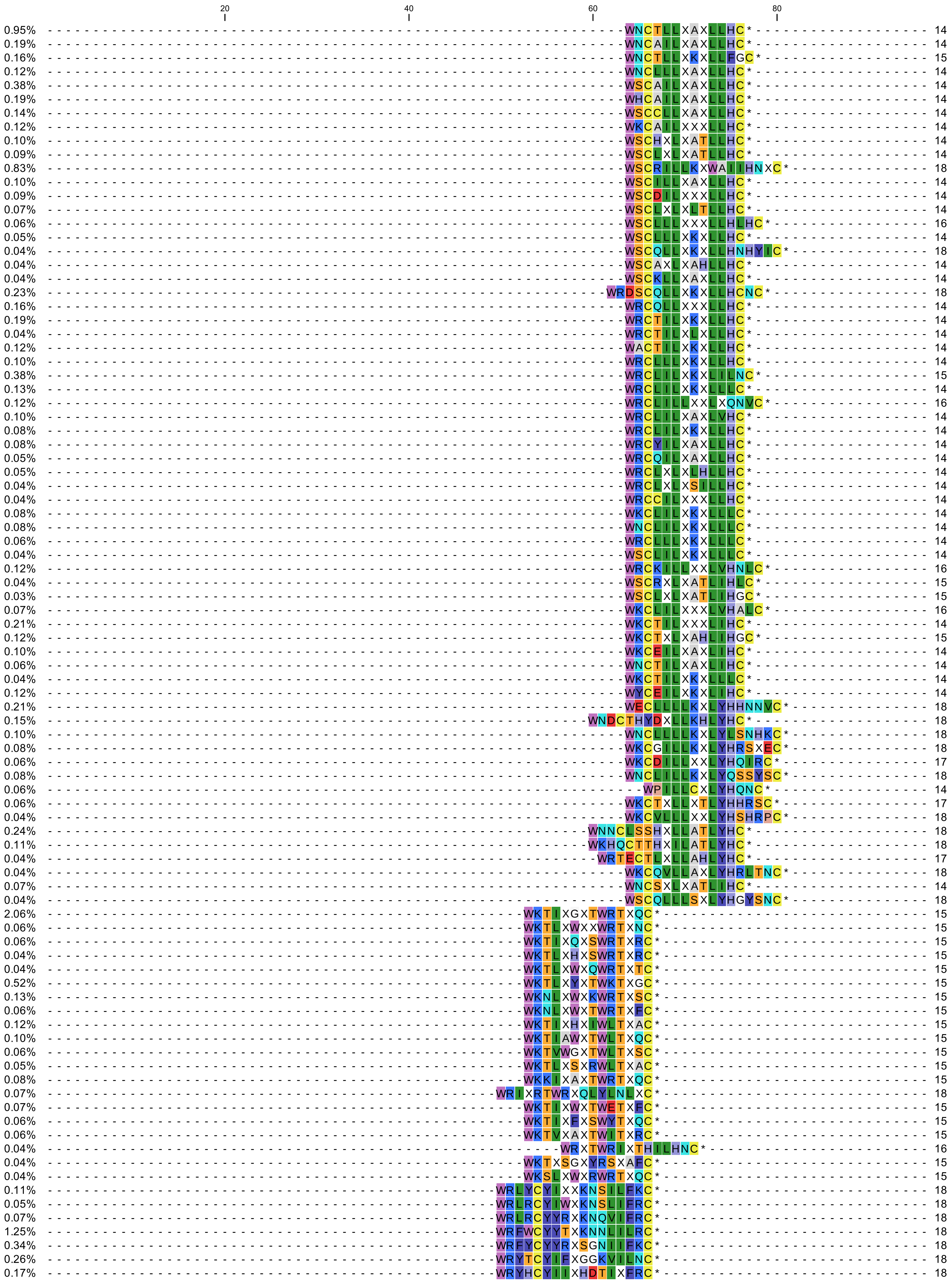
20
40
60
80

0.04%	WRYICYIWXKNRLLWNC*	18
0.12%	WRLACYIVXQQNIVWNC*	18
0.04%	WRLYCYITXNYQLIWNC*	18
0.17%	WRLTCYIWXGSKWLINC*	18
0.05%	WRLSCFIIXKNQFICRC*	18
0.05%	WRLACFYVXKSQIVFRC*	18
1.70%	WKFVCWIHXRNQLIFKC*	18
0.29%	WRLKCWILXKGVYYSRC*	18
0.09%	WRLTCWINXRGVLIRNC*	18
0.16%	WQRWXVPXSKIFKGXC*	17
0.11%	WRXICWIKXGPYLIWNC*	18
0.05%	WRFVCWIRXRTEVIWNC*	18
0.10%	WRXYCWIQXKSNTLYRC*	18
0.08%	WECWILXAXLLHWC*	15
0.08%	WYRHLLYSXKNALTFKC*	18
0.08%	WRYTCWYIXNXNLIYRC*	18
0.07%	WRYTCWIYXQGRYIYAC*	18
0.04%	WRYLQYRHXNQWTLIYC*	18
0.06%	WRXQLILRXYIYGRWHC*	18
0.05%	WXARFSEXLVRLIFC*	16
0.04%	WRXFCWIVXCDRFLHRC*	18
0.37%	WRCNILXAXLHNNWRFC*	18
0.25%	WIIPXVXLNWLRC*	14
0.11%	WIIPXKXLNWVLC*	14
0.08%	WWLFPXVRXLHSFXLRC*	18
0.07%	WKYECYYRXKGQTIYKC*	18
"2.62%	WTYLRWTXLTRIWISC*	17
0.13%	WTYLLXKAXNQYTRIWC*	18
0.24%	WKHWQAXPWVTISC*	15
0.06%	WKNWQRXPWVTISC*	15
0.12%	WIYWKYSYXNIWTRITC*	18
0.10%	WDAXRISCXHWNLAIRC*	18
0.07%	WTYVKYYWXXYVKILRC*	18
0.07%	WSRYAKSIXLTRXVILC*	18
0.06%	WLHTWPLXRIWYLXYC*	17
0.05%	WTYVXFRYXLRSLVVRC*	18
0.04%	WIYRKWISXLSYKIIVC*	18
0.04%	WTYFIWXLQRRIFC*	15
0.04%	WRWTCNSAXLLATLXHC*	18
0.04%	WTYLLWXSKKRLYC*	15
0.07%	WYGLACRXAGAILRC*	16
1.66%	WYSXKYAXWWTVYPC*	16
0.04%	WYSXEHAXWWTRYPC*	16
0.07%	WYSXKYAXXWKNDPC*	16
0.05%	WPFHLWYSXARALSYPC*	18
0.04%	WYSXKYAXVWTELPDC*	17
0.10%	WYSXTHAXSWTRLPRC*	17
0.06%	WYSXIWTLXWTRLPRDC*	18
0.05%	WYSXRRAXLWQEDPC*	16
0.04%	WNRYSWYSXRRALLWSC*	18
0.04%	WYSXARAXLWQSFPYC*	17
0.57%	WYSXTLAXHKSRXLC*	16
0.35%	WFEWYSXXYALQYAC*	16
0.20%	WYSXRHAXSWLQYPKC*	17
0.07%	WPLNAWYSXSNALQYTC*	18
0.11%	WYSXKHAXLWASAPKC*	17
0.06%	WYSXKHALXYYRAWLQC*	18
0.15%	WYSXKRAXLWTHNPC*	16
0.10%	WYSXKHAXIYRQNPC*	16
0.05%	WYSXXKAXLWTSHPC*	16
0.05%	WYSXRWAXSLPLWASC*	17
0.04%	WYSXQHAXNWSRHPC*	16
0.26%	WYSXRSPLXYYKYWXTC*	18
0.19%	WYSXRHTLXSYNLWLLC*	18
0.12%	WYSXKHTIXTFWAYLLC*	18
0.16%	WYSXQYAXHWKALPC*	16
0.10%	WYSXQYAXRWKQAPAC*	17
0.04%	WYDXSTAXRWKRYPAC*	17
0.09%	WWTIPXVKXGC*	12
0.08%	WWTIPXVKAGC*	12
0.07%	WYQHYYSXKYALRYCC*	17
0.05%	WSWYYSXXYALRYSC*	16
0.04%	WANEYYSXKYALQYQC*	17
0.06%	WYSXRYTLXFTSLTHWC*	18
0.11%	WWVIPXKIAHHC*	13
0.08%	WWIIPXKIAHHC*	13
0.05%	WWIIPXKIAYPC*	13
0.04%	WWIIPXKIAFHC*	13
0.08%	WWIIPXVKVFHC*	13
0.05%	WYTXKYAXREGYXPC*	16
0.16%	WWKIPXKKLGCVGLGLGLR*	20
0.05%	WKIPXKXVYPRCVGLGLGLR*	21
0.07%	WSKXYRXXKITCVGLGLGLR*	21
0.05%	WIIPXRXVNAKCVGLGLGLR*	21
0.06%	WWDWLIPXVRLRCSVGLGLGLR*	23
0.06%	WXGLRGRXFTKTNLIC*	17
0.05%	WWRFLQAQXYTRXILLC*	18
0.04%	WRFVLTVXVRNRXRLLC*	18
0.05%	WRFTCYIIXHNSYLVNC*	18
0.54%	WWIIPXKKAGC*	12
0.17%	WWIIPXVIAGC*	12
0.13%	WWIVPXKTAGC*	12
0.12%	WWIIPXKKAALC*	13

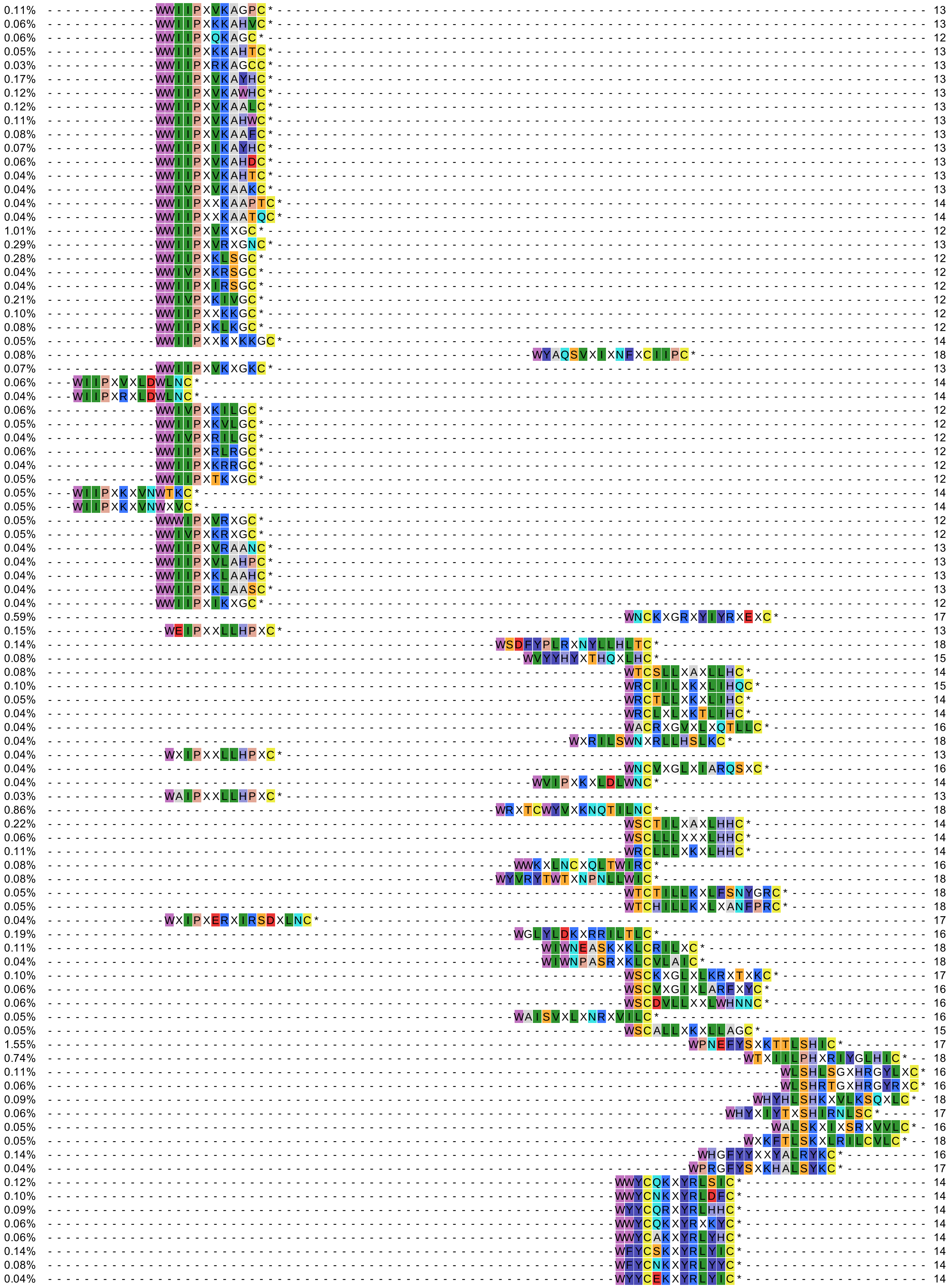

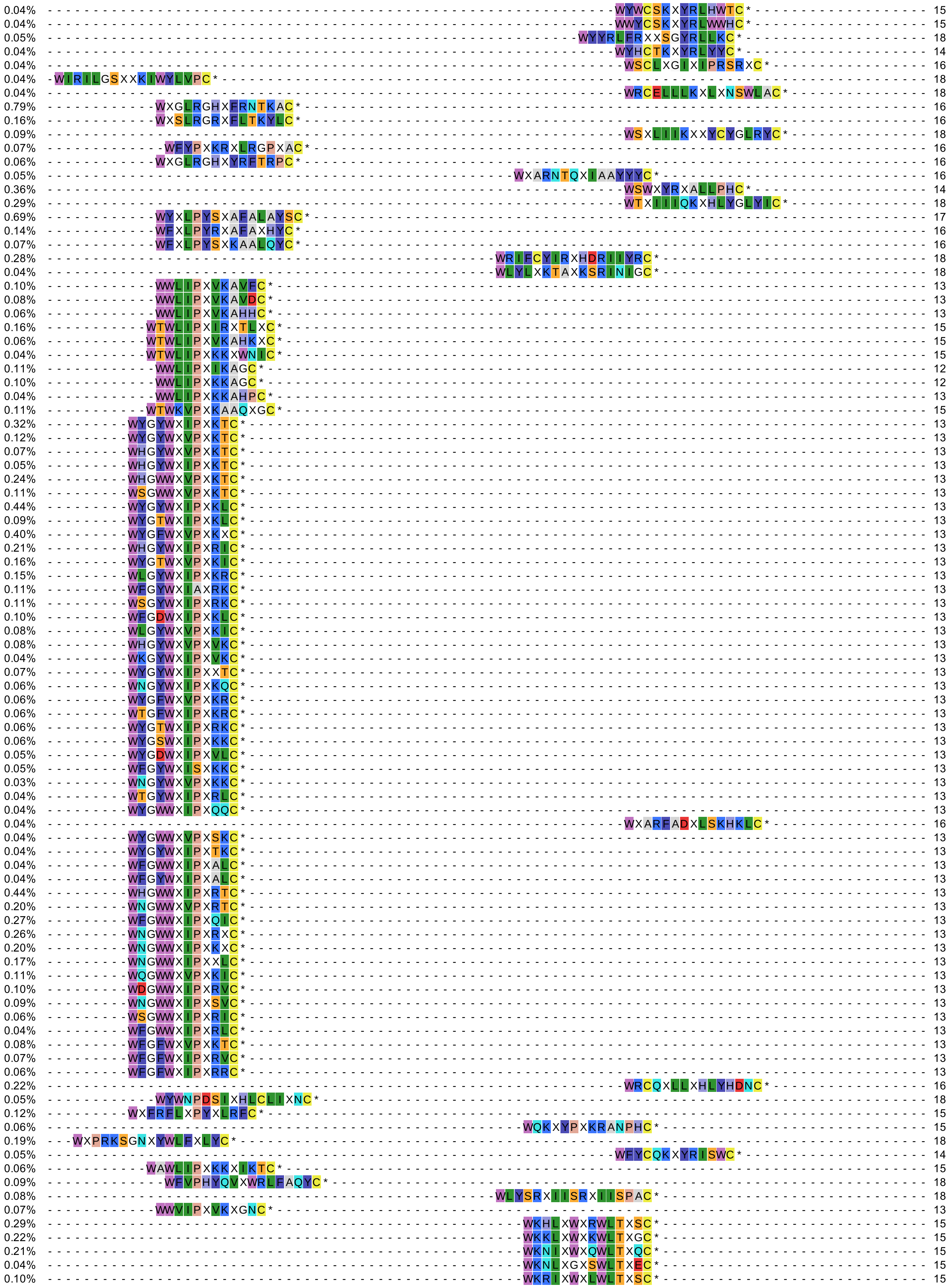

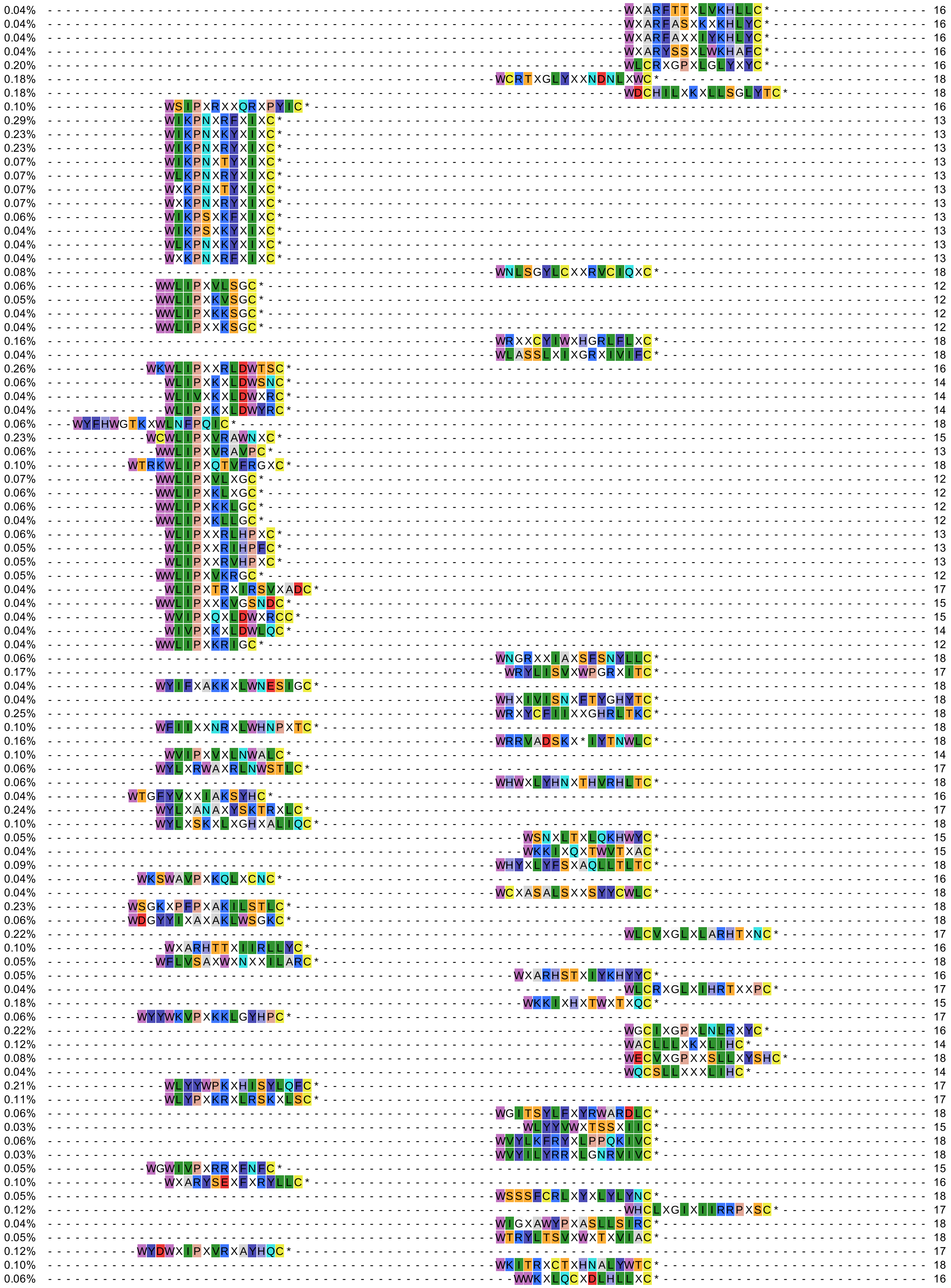

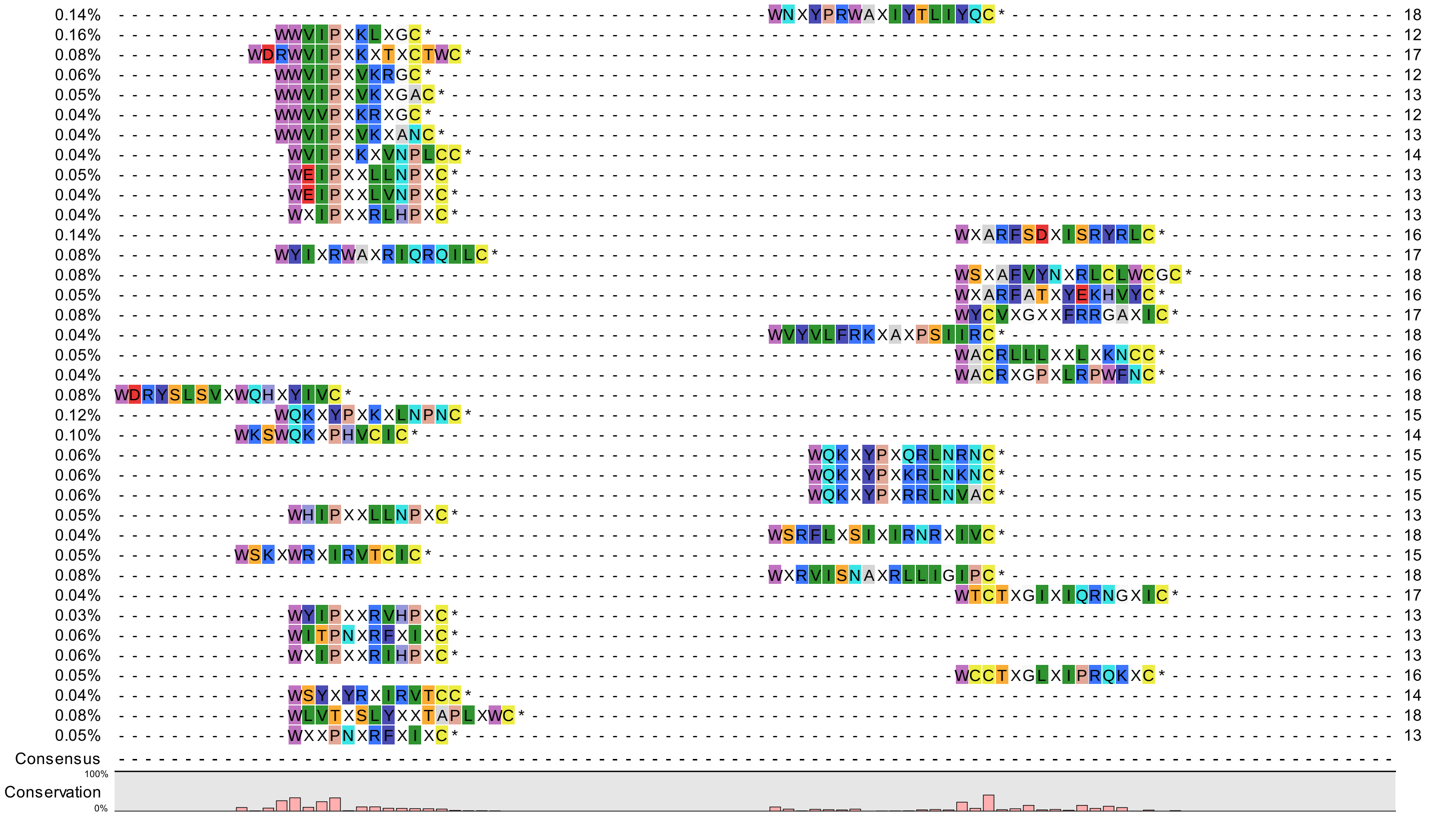
0.14% WNXYPRWAXIYTLIYQC* 18
0.16% WWVIPXKLXGC* 12
0.08% WDRWVIPXKXTXCTWC* 17
0.06% WWVIPXVKRGC* 12
0.05% WWVIPXVKXGAC* 13
0.04% WWVVPXKRXGC* 12
0.04% WWVIPXVKXANC* 13
0.04% WVIPXKXVNPLCC* 14
0.05% WEIPXXLLNPXC* 13
0.04% WEIPXXLVNPXC* 13
0.04% WXIPXXRLHPXC* 13
0.14% WXARFSDXISRYRLC* 16
0.08% WYIXRWAXRIQRQILC* 17
0.08% WSXAFVYNXRLCLWCGC* 18
0.05% WXARFATXYEKHVYC* 16
0.08% WYCVXGXXFRRGAXIC* 17
0.04% WVYVLFRKXAXPSIIRC* 18
0.05% WACRLLLXXLXKNCC* 16
0.04% WACRXGPXLRPWFNC* 16
0.08% WDRYSLSVXWQHXYIVC* 18
0.12% WQKXYPXKXLNPNC* 15
0.10% WKSWQKXPHVCIC* 14
0.06% WQKXYPXQRLNRNC* 15
0.06% WQKXYPXKRLNKNC* 15
0.06% WQKXYPXRRLNVAC* 15
0.05% WHIPXXLLNPXC* 13
0.04% WSRFLXSIXIRNRXIVC* 18
0.05% WSKXWRXIRVTCIC* 15
0.08% WXRVISNAXRLLIGIPC* 18
0.04% WTCTXGIXIQRNGXIC* 17
0.03% WYIPXXRVHPXC* 13
0.06% WITPNXRFXIXC* 13
0.06% WXIPXXRIHPXC* 13
0.05% WCCTXGLXIPRQKXC* 16
0.04% WSYXYRXIRVTCC* 14
0.08% WLVTXSLYXXTAPLXWC* 18
0.05% WXXPNXRFXIXC* 13
Consensus
Conservation
100%
0%
