# Supplementary material for: Cyclic peptides can engage a single binding pocket through highly divergent modes
Source: Proc Natl Acad Sci U S A. 2020 Oct 12;117(43):26728–38. doi: 10.1073/pnas.2003086117 (PMC7604503; doi:10.1073/pnas.2003086117)
Supplement: Supplementary File [file pnas.2003086117.sd04.pdf]

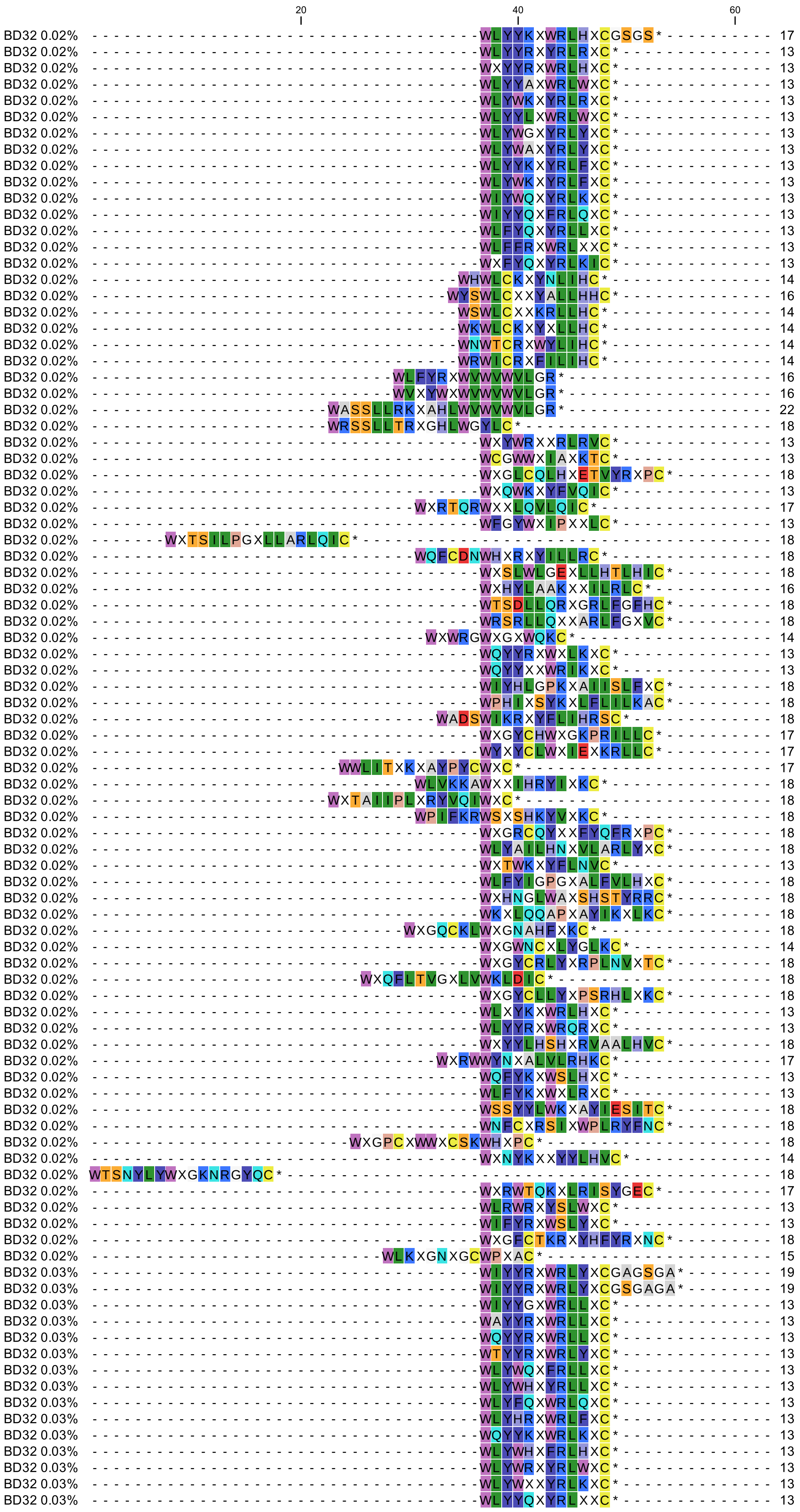
20
40
60

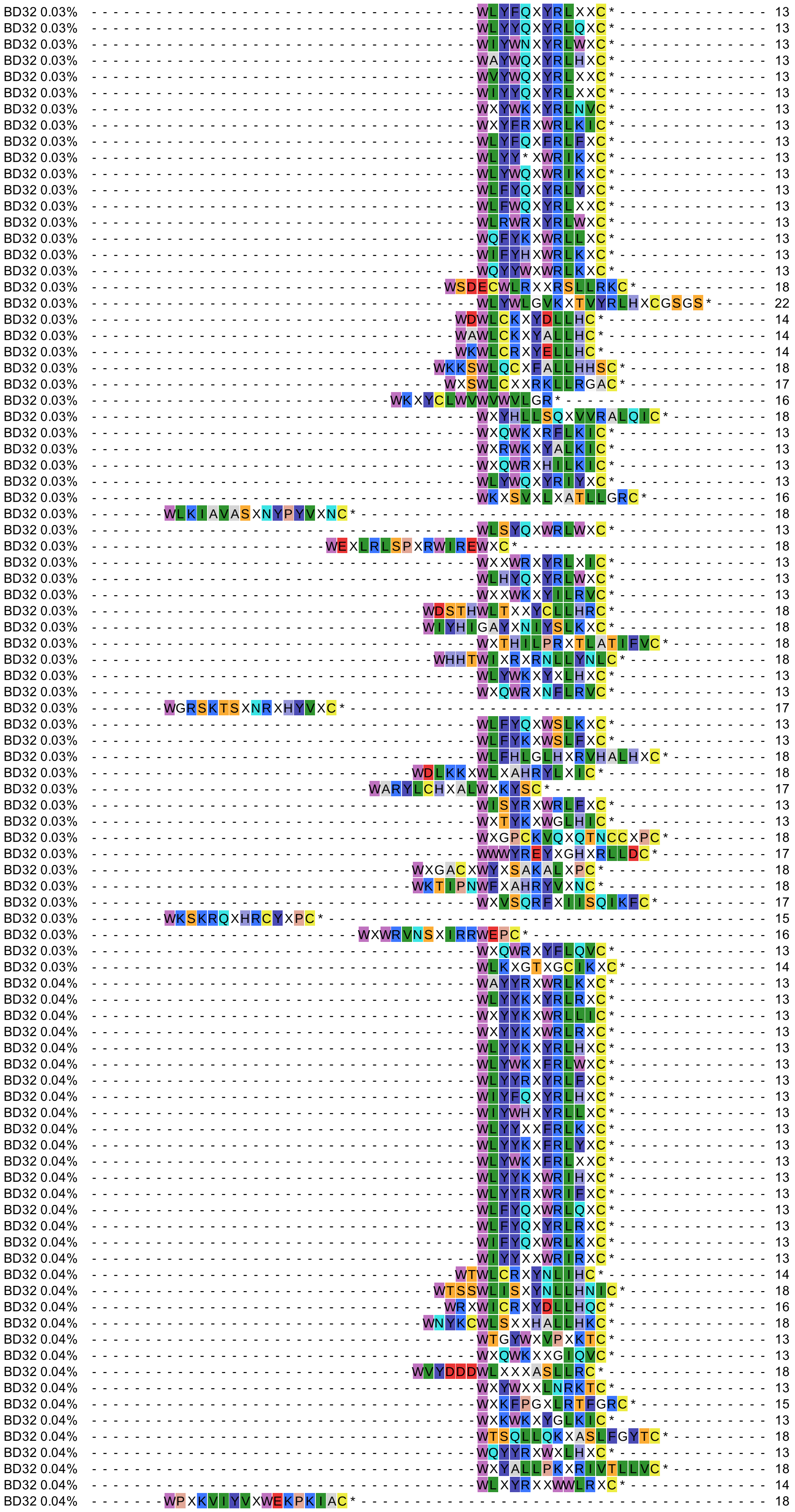

BD32 0.04%	WKKWAWNIXRPYRGQYC*	18
BD32 0.04%	WISWKXYRLWXC*	13
BD32 0.04%	WXXWQXYRLXIC*	13
BD32 0.04%	WITNQKWXVVATLYXC*	17
BD32 0.04%	WXRCSWQFXXKLLHLSC*	18
BD32 0.04%	WIYCPFNXAIRVRXC*	16
BD32 0.04%	WIHYQXWRLHXC*	13
BD32 0.04%	WXGPCTIFXQAHCRXRC*	18
BD32 0.04%	WLYYRXWSLHXC*	13
BD32 0.04%	WLYYKXWXLRXC*	13
BD32 0.04%	WLYWQXYXLFXC*	13
BD32 0.04%	WXQWKXNFLRVC*	13
BD32 0.04%	WXGLCXYRXTHPHLXSC*	18
BD32 0.04%	WLXHLGPKXALWRLKXC*	18
BD32 0.04%	WXXWIDKGXIYYSLXVC*	18
BD32 0.04%	WRRESNISXAWKQLNXC*	18
BD32 0.04%	WXTYILPRXIVAKVKVC*	18
BD32 0.04%	WXYWXXFRLKVC*	13
BD32 0.04%	WXGYCHVWXKPSGRXPC*	18
BD32 0.04%	WIXWKXYFLRXC*	13
BD32 0.04%	WXGKXCCXGWGQYQXC*	17
BD32 0.05%	WIYYRXWRLYXCGSGSGA*	19
BD32 0.05%	WXYYRXWRLXIC*	13
BD32 0.05%	WIYWNXYRLRXC*	13
BD32 0.05%	WIYFNXWRLKXC*	13
BD32 0.05%	WTYYRXWRLRXC*	13
BD32 0.05%	WLYYXXYRLRXC*	13
BD32 0.05%	WLYYLXYRLHXC*	13
BD32 0.05%	WLYYXXYRLYXC*	13
BD32 0.05%	WLYYRXYRLXXC*	13
BD32 0.05%	WQYFRXWRLHXC*	13
BD32 0.05%	WLFYRXYRLHXC*	13
BD32 0.05%	WXFWRXYRLQIC*	13
BD32 0.05%	WIYYKXWVWVWVLGR*	16
BD32 0.05%	WLYYQXWVWVWVLGR*	16
BD32 0.05%	WSGWWXIPXRIC*	13
BD32 0.05%	WNVXYWXXYNYPC*	14
BD32 0.05%	WLLYRXWRLWXC*	13
BD32 0.05%	WXQWKXYILKIC*	13
BD32 0.05%	WDXSYLSRXWYLIHQGC*	18
BD32 0.05%	WISWQXYRLRXC*	13
BD32 0.05%	WLHYQXWRLKXC*	13
BD32 0.05%	WIRVFKLYXGDWRLLTC*	18
BD32 0.05%	WXHYIAAGXRLYTLHVC*	18
BD32 0.05%	WLYYQXWSLWXC*	13
BD32 0.05%	WRSYWCWXXKFLXTC*	16
BD32 0.05%	WXGPCTNNXGRYLXHLC*	18
BD32 0.05%	WNIXYWXKLNLSC*	14
BD32 0.05%	WIYYRXWXLNXC*	13
BD32 0.05%	WXQWQXFFLRIC*	13
BD32 0.06%	WLYYXXYRLLXC*	13
BD32 0.06%	WLYFQXYRLRXC*	13
BD32 0.06%	WLYWQXWRLRXC*	13
BD32 0.06%	WLYYNXWRLXXC*	13
BD32 0.06%	WLYWKXYRLHXC*	13
BD32 0.06%	WXYYQXWRLRXC*	13
BD32 0.06%	WLYWGXYRLQXC*	13
BD32 0.06%	WLYYQXYRLKXC*	13
BD32 0.06%	WIYWNXYRLKXC*	13
BD32 0.06%	WIYYRXYRLYXC*	13
BD32 0.06%	WIYYQXYRLLXC*	13
BD32 0.06%	WLYYQXFRLYXC*	13
BD32 0.06%	WLYWKXFRLYXC*	13
BD32 0.06%	WXYWKXFRLQVC*	13
BD32 0.06%	WLYYRXWRIQXC*	13
BD32 0.06%	WLFYKXWRLLXC*	13
BD32 0.06%	WLFWKXYRLQXC*	13
BD32 0.06%	WHTWSCXXYYLHHRC*	16
BD32 0.06%	WSWLCRXYNLHHC*	14
BD32 0.06%	WLSWICAXYRLLHGC*	16
BD32 0.06%	WYACWLKKXYXLLHNRC*	18
BD32 0.06%	WFDDWCVRXYILLHHSC*	18
BD32 0.06%	WTGYWXIPXKTC*	13
BD32 0.06%	WNVXYWXKYKLRC*	14
BD32 0.06%	WLYYLSFHXTLHKLWXC*	18
BD32 0.06%	WSDSYNFWXGWTKGYSC*	18
BD32 0.06%	WXQWKXYFTKIC*	13
BD32 0.06%	WXQWKXXGLNIC*	13
BD32 0.06%	WXFHLGPAXVLWSVHIC*	18
BD32 0.06%	WXFYRXWGLKIC*	13
BD32 0.06%	WXSWKXYFLKIC*	13
BD32 0.06%	WQYHLGSQXVLFKLFXC*	18
BD32 0.06%	WISWQXYRLKXC*	13
BD32 0.06%	WLXWRXYRLYXC*	13
BD32 0.06%	WXYYILRRXQLVSLNIC*	18
BD32 0.06%	WXTYIAPGXIYHXLNVC*	18
BD32 0.06%	WXTWKXYFLNIC*	13
BD32 0.06%	WXGSCTYRXSYPHAXPC*	18
BD32 0.06%	WLXYQXWRLHXC*	13
BD32 0.06%	WLFGLLTRXQVVRLQXC*	18
BD32 0.06%	WLYYQXWRVRXC*	13
BD32 0.06%	WXGLCHFNXNAKYYXPC*	18
BD32 0.06%	WXTFVTYKXRYLALRIC*	18

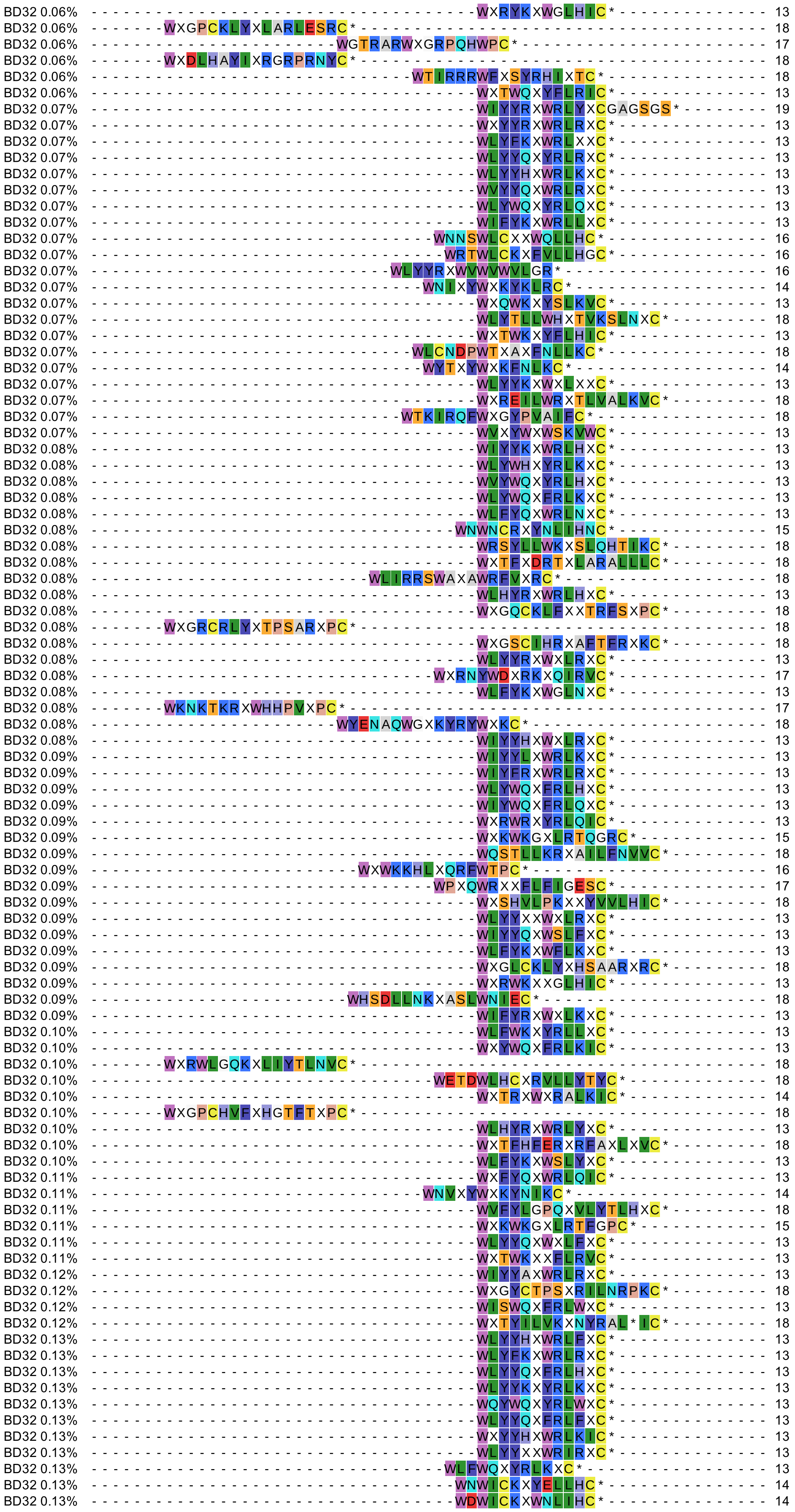
BD32 0.06% WXRYKXWGLHIC* 13
BD32 0.06% WXGPCKLYXLARLESRC* 18
BD32 0.06% WGTRARWXGRPQHWPC* 17
BD32 0.06% WXDLHAYIXRGRPRNYC* 18
BD32 0.06% WTIRRRWFXSYRHIXTC* 18
BD32 0.06% WXTWQXYFLRIC* 13
BD32 0.07% WIYYRXWRLYXCGAGSGS* 19
BD32 0.07% WXYYRXWRLRXC* 13
BD32 0.07% WLYFKXWRLXXC* 13
BD32 0.07% WLYYQXYRLRXC* 13
BD32 0.07% WLYYHXWRLKXC* 13
BD32 0.07% WVYYQXWRLRXC* 13
BD32 0.07% WLYWQXYRLQXC* 13
BD32 0.07% WIFYKXWRLLXC* 13
BD32 0.07% WNNSWLCXXWQLLHC* 16
BD32 0.07% WRTWLCKXFVLLHGC* 16
BD32 0.07% WLYYRXWVWVWVLGR* 16
BD32 0.07% WNIXYWXKYKLRC* 14
BD32 0.07% WXQWKXYSLKVC* 13
BD32 0.07% WLYTLLWHXTVKSLNXC* 18
BD32 0.07% WXTWKXYFLHIC* 13
BD32 0.07% WLCNDPWTXAXFNLLKC* 18
BD32 0.07% WYTXYWXKFNLKC* 14
BD32 0.07% WLYYKXWXLXXC* 13
BD32 0.07% WXREILWRXTLVALKVC* 18
BD32 0.07% WTKIRQFWXGYPVAIFC* 18
BD32 0.07% WVXYWXWSKVWC* 13
BD32 0.08% WIYYKXWRLHXC* 13
BD32 0.08% WLYWHXYRLKXC* 13
BD32 0.08% WVYWQXYRLHXC* 13
BD32 0.08% WLYWQXFRLKXC* 13
BD32 0.08% WLFYQXWRLNXC* 13
BD32 0.08% WNWNCRXYNLIHNC* 15
BD32 0.08% WRSYLLWKXSLQHTIKC* 18
BD32 0.08% WXTFXDRTXLARALLLC* 18
BD32 0.08% WLIRRSWAXAWRFVXRC* 18
BD32 0.08% WLHYRXWRLHXC* 13
BD32 0.08% WXGQCKLFXXTRFSXPC* 18
BD32 0.08% WXGRCRLYXTPSARXPC* 18
BD32 0.08% WXGSCIHRXAFTFRXKC* 18
BD32 0.08% WLYYRXWXLRXC* 13
BD32 0.08% WXRNYWDXRKXQIRVC* 17
BD32 0.08% WLFYKXWGLNXC* 13
BD32 0.08% WKNKTKRXWHHPVXPC* 17
BD32 0.08% WYENAQWGXKYRYWXKC* 18
BD32 0.08% WIYYHXWXLRXC* 13
BD32 0.09% WIYYLXWRLKXC* 13
BD32 0.09% WIYFRXWRLRXC* 13
BD32 0.09% WLYWQXFRLHXC* 13
BD32 0.09% WIYWQXFRLQXC* 13
BD32 0.09% WXRWRXYRLQIC* 13
BD32 0.09% WXKWKGXLRTQGRC* 15
BD32 0.09% WQSTLLKRXAILFNVVC* 18
BD32 0.09% WXWKKHLXQRFWTPC* 16
BD32 0.09% WPXQWRXXFLFIGESC* 17
BD32 0.09% WXSHVLPKXXYVVLHIC* 18
BD32 0.09% WLYYXXWXLRXC* 13
BD32 0.09% WIYYQXWSLFXC* 13
BD32 0.09% WLFYKXWFLKXC* 13
BD32 0.09% WXGLCKLYXHSAARXRC* 18
BD32 0.09% WXRWKXXGLHIC* 13
BD32 0.09% WHSDLLNKXASLWNIEC* 18
BD32 0.09% WIFYRXWXLKXC* 13
BD32 0.10% WLFWKXYRLLXC* 13
BD32 0.10% WXYWQXFRLKIC* 13
BD32 0.10% WXRWLGQKXLIYTLNVC* 18
BD32 0.10% WETDWLHCXRVLLYTYC* 18
BD32 0.10% WXTRXWXRALKIC* 14
BD32 0.10% WXGPCHVFXHGTFTXPC* 18
BD32 0.10% WLHYRXWRLYXC* 13
BD32 0.10% WXTFHFERXRFAXLXVC* 18
BD32 0.10% WLFYKXWSLYXC* 13
BD32 0.11% WXFYQXWRLQIC* 13
BD32 0.11% WNVXYWXKYNIKC* 14
BD32 0.11% WVFYLGPQXVLYTLHXC* 18
BD32 0.11% WXKWKGXLRTFGPC* 15
BD32 0.11% WLYYQXWXLFXC* 13
BD32 0.11% WXTWKXXFLRVC* 13
BD32 0.12% WIYYAXWRLRXC* 13
BD32 0.12% WXGYCTPSXRILNRPKC* 18
BD32 0.12% WISWQXFRLWXC* 13
BD32 0.12% WXTYILVKXNYRAL*IC* 18
BD32 0.13% WLYYHXWRLFXC* 13
BD32 0.13% WLYFKXWRLRXC* 13
BD32 0.13% WLYYQXFRLHXC* 13
BD32 0.13% WLYYKXYRLKXC* 13
BD32 0.13% WQYWQXYRLWXC* 13
BD32 0.13% WLYYQXFRLFXC* 13
BD32 0.13% WXYYHXWRLKIC* 13
BD32 0.13% WLYYXXWRIRXC* 13
BD32 0.13% WLFWQXYRLKXC* 13
BD32 0.13% WNWICKXYELLHC* 14
BD32 0.13% WDWICKXWNLIHC* 14

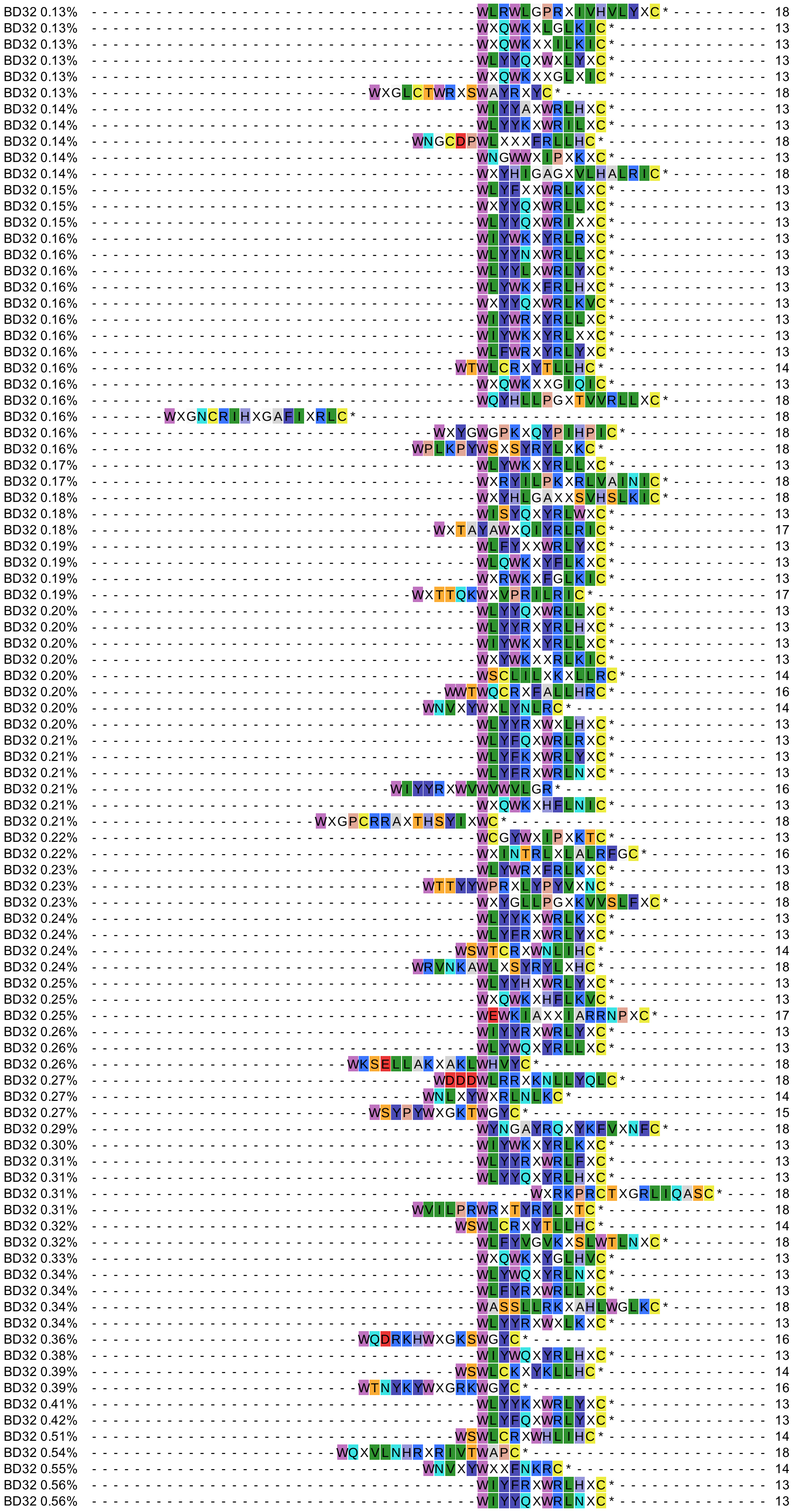
BD32 0.13% WLRWLGPRXIVHVLYXC* 18
BD32 0.13% WXQWKXLGLKIC* 13
BD32 0.13% WXQWKXXILKIC* 13
BD32 0.13% WLYYQXWXLYXC* 13
BD32 0.13% WXQWKXXGLXIC* 13
BD32 0.13% WXGLCTWRXSWAYRXYC* 18
BD32 0.14% WIYYAXWRLHXC* 13
BD32 0.14% WLYYKXWRILXC* 13
BD32 0.14% WNGCDPWLXXXFRLLHC* 18
BD32 0.14% WNGWWXIPXKXC* 13
BD32 0.14% WXYHIGAGXVLHALRIC* 18
BD32 0.15% WLYFXXWRLKXC* 13
BD32 0.15% WXYYQXWRLLXC* 13
BD32 0.15% WLYYQXWRIXXC* 13
BD32 0.16% WIYWKXYRLRXC* 13
BD32 0.16% WLYYNXWRLLXC* 13
BD32 0.16% WLYYLXWRLYXC* 13
BD32 0.16% WLYWKXFRLHXC* 13
BD32 0.16% WXYYQXWRLKVC* 13
BD32 0.16% WIYWRXYRLLXC* 13
BD32 0.16% WIYWKXYRLXXC* 13
BD32 0.16% WLFWRXYRLYXC* 13
BD32 0.16% WTWLCRXYTLLHC* 14
BD32 0.16% WXQWKXXGIQIC* 13
BD32 0.16% WQYHLLPGXTVVRLLXC* 18
BD32 0.16% WXGNCRIHXGAFIXRLC* 18
BD32 0.16% WXYGWGPKXQYPIHPIC* 18
BD32 0.16% WPLKPYWSXSYRYLXKC* 18
BD32 0.17% WLYWKXYRLLXC* 13
BD32 0.17% WXRYILPKXRLVAINIC* 18
BD32 0.18% WXYHLGAXXSVHSLKIC* 18
BD32 0.18% WISYQXYRLWXC* 13
BD32 0.18% WXTAYAWXQIYRLRIC* 17
BD32 0.19% WLFYXXWRLYXC* 13
BD32 0.19% WLQWKXYFLKXC* 13
BD32 0.19% WXRWKXFGLKIC* 13
BD32 0.19% WXTTQKWXVPRILRIC* 17
BD32 0.20% WLYYQXWRLLXC* 13
BD32 0.20% WLYYRXYRLHXC* 13
BD32 0.20% WIYWKXYRLLXC* 13
BD32 0.20% WXYWKXXRLKIC* 13
BD32 0.20% WSCLILXKXLLRC* 14
BD32 0.20% WWTWQCRXFALLHRC* 16
BD32 0.20% WNVXYWXLYNLRC* 14
BD32 0.20% WLYYRXWXLHXC* 13
BD32 0.21% WLYFQXWRLRXC* 13
BD32 0.21% WLYFKXWRLYXC* 13
BD32 0.21% WLYFRXWRLNXC* 13
BD32 0.21% WIYYRXWVWVWVLGR* 16
BD32 0.21% WXQWKXHFLNIC* 13
BD32 0.21% WXGPCRRAXTHSYIXWC* 18
BD32 0.22% WCGYWXIPXKTC* 13
BD32 0.22% WXINTRLXLALRFGC* 16
BD32 0.23% WLYWRXFRLKXC* 13
BD32 0.23% WTTYYWPRXLYPYVXNC* 18
BD32 0.23% WXYGLLPGXKVVSLFXC* 18
BD32 0.24% WLYYKXWRLKXC* 13
BD32 0.24% WLYFRXWRLYXC* 13
BD32 0.24% WSWTCRXWNLIHC* 14
BD32 0.24% WRVNKAWLXSYRYLXHC* 18
BD32 0.25% WLYYHXWRLYXC* 13
BD32 0.25% WXQWKXHFLKVC* 13
BD32 0.25% WEWKIAXXIARRNPXC* 17
BD32 0.26% WIYYRXWRLYXC* 13
BD32 0.26% WLYWQXYRLLXC* 13
BD32 0.26% WKSELLAKXAKLWHVYC* 18
BD32 0.27% WDDDWLRRXKNLLYQLC* 18
BD32 0.27% WNLXYWXRLNLKC* 14
BD32 0.27% WSYPYWXGKTWGYC* 15
BD32 0.29% WYNGAYRQXYKFVXNFC* 18
BD32 0.30% WIYWKXYRLKXC* 13
BD32 0.31% WLYYRXWRLFXC* 13
BD32 0.31% WLYYQXYRLHXC* 13
BD32 0.31% WXRKPRCTXGRLIQASC* 18
BD32 0.31% WVILPRWRXTYRYLXTC* 18
BD32 0.32% WSWLCRXYTLLHC* 14
BD32 0.32% WLFYVGVKXSLWTLNXC* 18
BD32 0.33% WXQWKXYGLHVC* 13
BD32 0.34% WLYWQXYRLNXC* 13
BD32 0.34% WLFYRXWRLLXC* 13
BD32 0.34% WASSLLRKXAHLWGLKC* 18
BD32 0.34% WLYYRXWXLKXC* 13
BD32 0.36% WQDRKHWXGKSWGYC* 16
BD32 0.38% WIYWQXYRLHXC* 13
BD32 0.39% WSWLCKXYKLLHC* 14
BD32 0.39% WTNYKYWXGRKWGYC* 16
BD32 0.41% WLYYKXWRLYXC* 13
BD32 0.42% WLYFQXWRLYXC* 13
BD32 0.51% WSWLCRXWHLIHC* 14
BD32 0.54% WQXVLNHRXRIVTWAPC* 18
BD32 0.55% WNVXYWXXFNKRC* 14
BD32 0.56% WIYFRXWRLHXC* 13
BD32 0.56% WIYYQXWRLNXC* 13

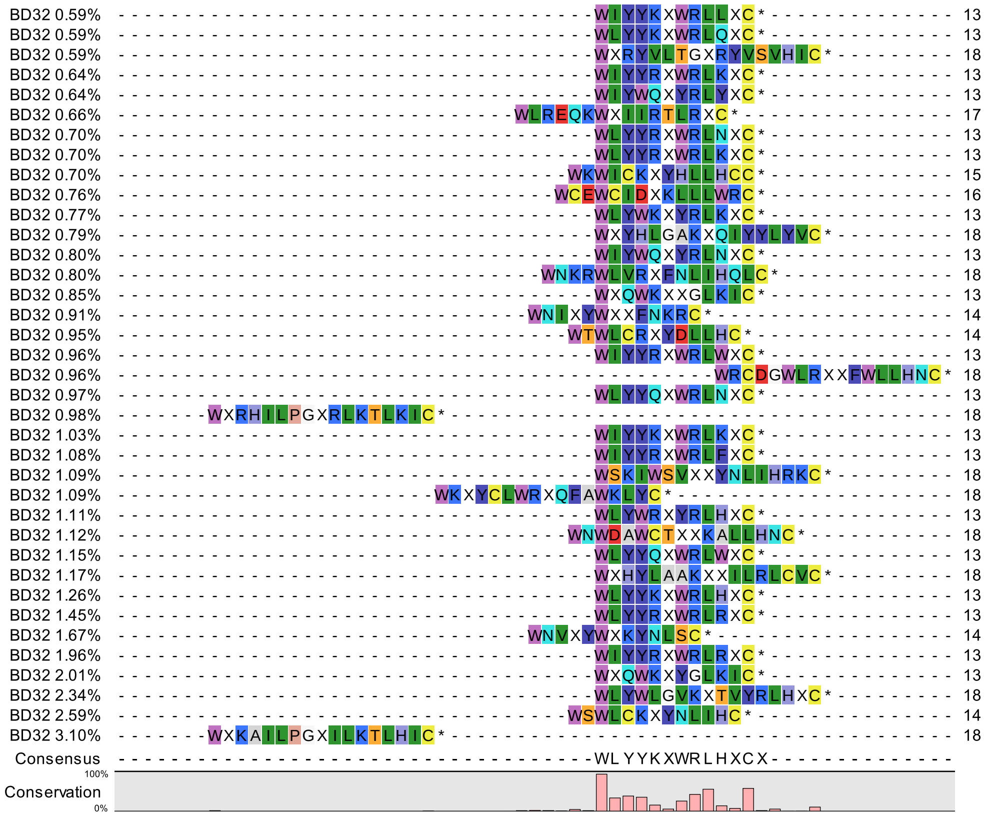
BD32 0.59% WIYYKXWRLLXC* 13
BD32 0.59% WLYYKXWRLQXC* 13
BD32 0.59% WXRYVLTGXRYVSVHIC* 18
BD32 0.64% WIYYRXWRLKXC* 13
BD32 0.64% WIYWQXYRLYXC* 13
BD32 0.66% WLREQKWXIIRTLRXC* 17
BD32 0.70% WLYYRXWRLNXC* 13
BD32 0.70% WLYYRXWRLKXC* 13
BD32 0.70% WKWICKXYHLLHCC* 15
BD32 0.76% WCEWCIDXKLLLWRC* 16
BD32 0.77% WLYWKXYRLKXC* 13
BD32 0.79% WXYHLGAKXQIYYLYVC* 18
BD32 0.80% WIYWQXYRLNXC* 13
BD32 0.80% WNKRWLVRXFNLIHQLC* 18
BD32 0.85% WXQWKXXGLKIC* 13
BD32 0.91% WNIXYWXXFNKRC* 14
BD32 0.95% WTWLCRXYDLLHC* 14
BD32 0.96% WIYYRXWRLWXC* 13
BD32 0.96% WRCDGWLRXXFWLLHNC* 18
BD32 0.97% WLYYQXWRLNXC* 13
BD32 0.98% WXRHILPGXRLKTLKIC* 18
BD32 1.03% WIYYKXWRLKXC* 13
BD32 1.08% WIYYRXWRLFXC* 13
BD32 1.09% WSKIWSVXXYNLIHRKC* 18
BD32 1.09% WKXYCLWRXQFAWKLYC* 18
BD32 1.11% WLYWRXYRLHXC* 13
BD32 1.12% WNWDAWCTXXKALLHNC* 18
BD32 1.15% WLYYQXWRLWXC* 13
BD32 1.17% WXHYLAAKXXILRLCVC* 18
BD32 1.26% WLYYKXWRLHXC* 13
BD32 1.45% WLYYRXWRLRXC* 13
BD32 1.67% WNVXYWXKYNLSC* 14
BD32 1.96% WIYYRXWRLRXC* 13
BD32 2.01% WXQWKXYGLKIC* 13
BD32 2.34% WLYWLGVKXTVYRLHXC* 18
BD32 2.59% WSWLCKXYNLIHC* 14
BD32 3.10% WXKAILPGXILKTLHIC* 18
Consensus WLYYKXWRLHXCX
Conservation
100%
0%
